# Mono- and dinuclear zirconocene(iv) amide complexes for the catalytic dehydropolymerisation of phenylsilane†

**DOI:** 10.1039/d2ra04955d

**Published:** 2022-09-15

**Authors:** Kevin Lindenau, Anke Spannenberg, Fabian Reiß, Torsten Beweries

**Affiliations:** Leibniz-Institut für Katalyse e.V. Albert-Einstein-Str. 29a 18059 Rostock Germany fabian.reiss@catalysis.de torsten.beweries@catalysis.de

## Abstract

The dehydropolymerisation of phenylsilane is investigated using group 4 metallocene amide complexes as catalysts. The dinuclear zirconocene amide complex Cp_2_Zr(NMe_2_)(μ-Me_3_SiC_3_SiMe_3_)Zr(NMe_2_)Cp_2_ (2) (Cp = η^5^-cyclopentadienyl) shows high activity in dehydrocoupling reactions, producing polyphenylsilanes with molecular weights ranging from 200 to 3000 g mol^−1^ and linear-to-cyclic product ratios of up to 80 : 20. Likewise, different ratios of oligomers and polymers with different tacticities could be described. *Ansa*-zirconocene amide complexes possessing the ebthi (ebthi = 1,2-ethylene-1,1′-bis(η^5^-tetrahydroindenyl)) ligand systems were prepared and evaluated for catalytic dehydropolymerisation in comparison to the dinuclear catalyst system.

## Abstract 2

Mono- and dinuclear zirconocene amide complexes were tested as catalysts for the dehydropolymerisation of phenylsilane. The dinuclear complex is surprisingly stable, producing mixtures of polysilanes and cyclic oligomers.

## Graphical abstract

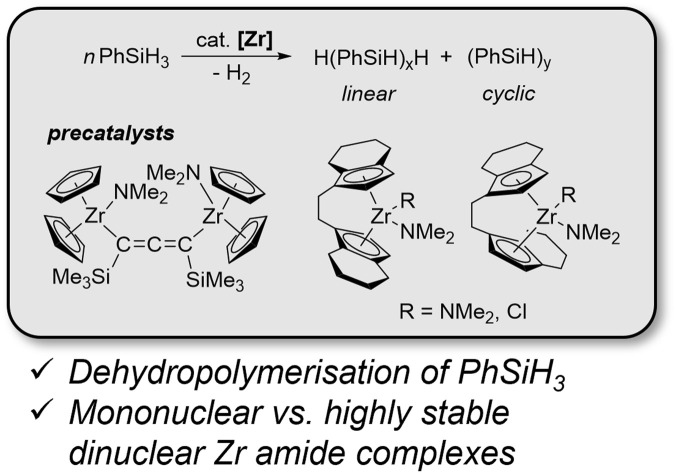

## Introduction

**Scheme 1:**
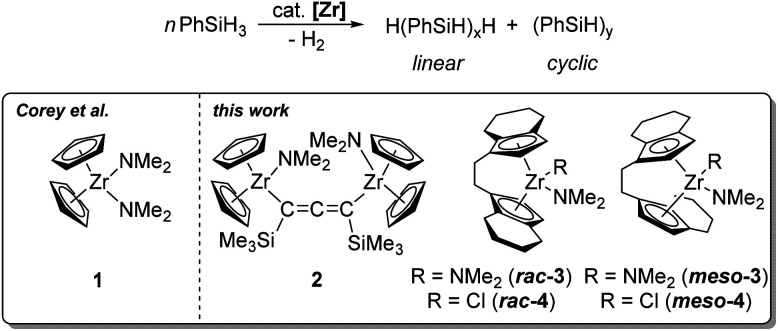
Catalytic dehydropolymerisation of PhSiH_3_ using different zirconocene amide complexes.

The catalytic formation of Si–Si bonds to produce inorganic–organic polymers and hybrid materials has been intensively studied since the 1980s.^1–5^ The special properties of polysilanes such as *σ* electron delocalisation have led to the application of these materials for the development of new ceramics, semiconductors and nonlinear optical materials.^4^ For converting silane-based monomers several different approaches are feasible.^6–9^ Catalytic dehydrocoupling of hydrosilanes represents an atom-efficient method compared to the Wurtz coupling that produces stoichiometric amounts of salts as waste products and shows a poor functional-group-tolerance.^10,11^ Early investigations focused on group 4 metallocene-based catalysts, although complexes of later transition metals were also studied and showed comparable catalytic performance in some cases.^12–18^ The main advantages are generally good yields (>90%), the possibility for ligand variation and post-polymerisation functionalisation.^1,19,20^ It should however be noted that typically low molecular weight polymers with ill-defined tacticity and poor linear-to-cyclic ratios are obtained.^1^ However, the utility of this approach for the construction of Si rich polymers containing poly(cyclosilane) units was recently demonstrated by Klausen and co-workers.^21,22^ For group 4 metallocene catalysis, experimental parameters like concentration effects, catalyst deactivation pathways, optimal metallocene substitution patterns, and the pressure dependence on molecular weight, were studied in great detail in the past, mainly by the groups of Corey, Harrod and Tilley.^12–14^ The dehydropolymerisation of the primary silane PhSiH_3_ using the system Cp_2_ZrCl_2_/*n*-BuLi was well-studied in the past, giving >99% polysilane with a mass distribution of *M*_n_/*M*_w_ = 3200/1600 g mol^−1^ and a ratio of linear/cyclic products of 65/35.^23–26^ These systems require the use of strong base for activation of the precatalyst. Formation of oligomers and polymers was described to occur *via σ* bond metathesis for group 4 metals.^27–29^ An alternative approach could be the use of well-defined highly reactive species such as metallocene alkyne complexes, silyl, alkyl, or amide complexes.^27,30–33^ In the area of dehydropolymerisation of PhSiH_3_, only a handful of zirconium amide complexes were described as catalysts.^34–36^ Corey *et al.* have reported the mononuclear complex Cp_2_Zr(NMe_2_)_2_ (1) as catalyst for the dehydropolymerisation of phenylsilanes (Scheme 1).^34^

Modification of the cyclopentadienyl scaffold is a common approach in group 4 metallocene chemistry, to synthesise optically active compounds or polymers with well-defined tacticity.^37–39^*Ansa*-zirconocene amide complexes have been reported by Jordan *et al.*, who demonstrated the selective synthesis of [*rac*/*meso*-(ebi)Zr(NMe_2_)_2_] (ebi = 1,2-ethylene-1,1′-bis(η^5^-indenyl)) by amine elimination.^40,41^ Likewise, this route furnishes other zirconocene amide complexes with an indenyl ligand system.^42,43^ Such complexes are used for a wide range of applications in asymmetric catalysis.^44^ Jordan *et al.* described the polymerisation of propene by using [*rac*-(ebi)Zr(NMe_2_)_2_] and [*rac*-(sbi)Zr(NMe_2_)_2_] (sbi = dimethylsilyl-1,1′-bis(η^5^-indenyl)) with additional MAO activation.^45^ The group showed that amide complexes are significantly less active compared to the chloride-related systems.^45^ Kim *et al.* described the formation of various cationic zirconocene species by using [*rac*-(ebi)Zr(NMe_2_)_2_] as catalyst for polymerisation of propene.^46,47^

Recently, our group synthesised a set of dinuclear zirconocene complexes, including the amide species Cp_2_Zr(NMe_2_)(μ-Me_3_SiC_3_SiMe_3_)Zr(NMe_2_)Cp_2_ (2) (Scheme 1) which exhibited high activity in the dehydrocoupling of H_3_B·NMe_2_H.^48–50^ Dinuclear cooperative reactivity of both metal centers was discussed as an important mechanistic feature. Intrigued by the high activity of this complex at room temperature, we aimed at the study of this species for related dehydrocoupling reactions such as the well-known dehydropolymerisation of PhSiH_3_. In this contribution, we describe the application of 2 as catalyst for the dehydropolymerisation of phenylsilane and discuss its performance in comparison to mononuclear complexes. Furthermore, the synthesis of a series of previously unknown *ansa*-zirconocene amide complexes is described, as well as the application of these complexes as single-component catalysts.

## Results and discussion

### Synthesis and characterisation of *ansa*-zirconocene amides

**Scheme 2:**
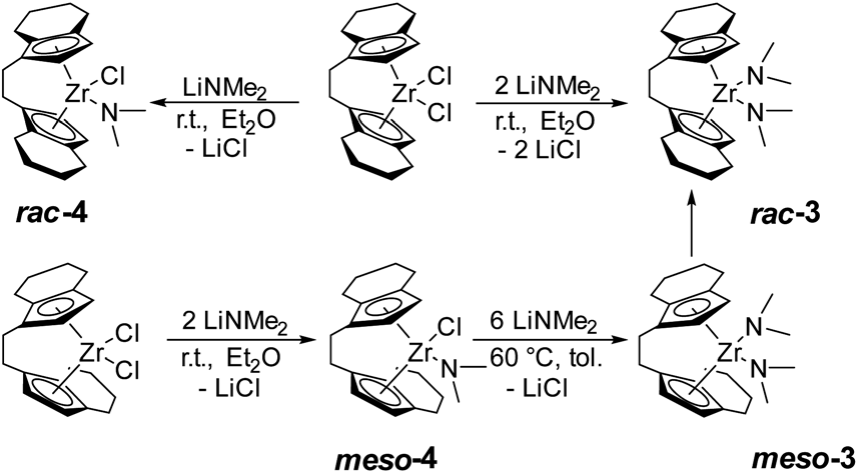
Synthesis of *ansa*-zirconocene amide complexes.

**Fig. 1:**
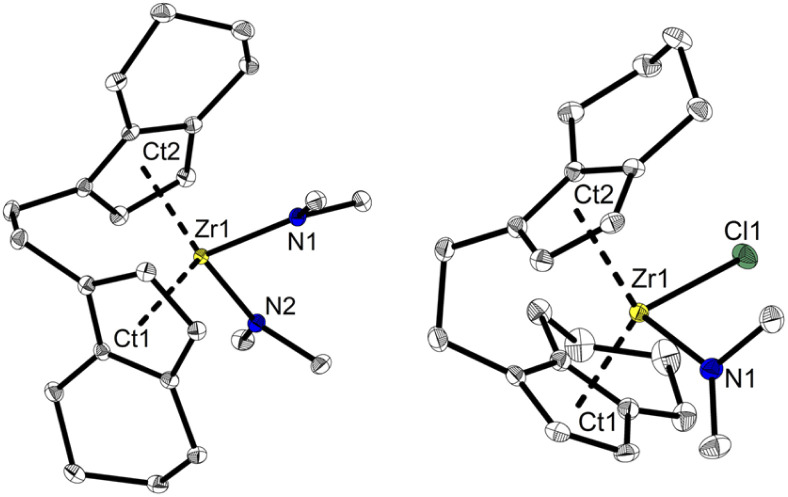
Molecular structures of complexes *rac*-3 and *meso*-4. Selected bond lengths and angles 3: Zr1–N1 2.1074(13), Zr1–N2 2.1065(13), Ct1–Zr1 2.284, Ct2–Zr1 2.286 [Å]; N1–Zr1–N2 95.09(5), Ct1–Zr1–Ct2 121.14, C_ind1_–C_et1_–C_et2_–C_ind2_–40.78(19)°. 4 Zr1–Cl1 2.4772(8), Zr1–N1 2.064(3), Ct1–Zr1 2.232, Ct2–Zr1 2.275 [Å]; N1–Zr1–Cl1 96.78(8), Ct1–Zr1–Ct2 123.55, C_ind1_–C_et1_–C_et2_–C_ind2_–46.2(4)°. Thermal ellipsoids correspond to 30% probability. Hydrogen atoms are omitted for clarity.^51^

Based on the previously reported reaction conditions for the preparation of the dinuclear amide complex 2,^48^ we carried out the reaction of [*rac*-(ebthi)ZrCl_2_] (ebthi = 1,2-ethylene-1,1′-bis(η^5^-tetrahydroindenyl)) with two equivalents of LiNMe_2_ in a similar fashion (Scheme 2). Formation of one main compound that shows three characteristic resonances in the ^1^H NMR spectrum in 2 : 2 : 12 ratio (*δ* 6.08, 5.31 and 2.97 ppm, Fig. S4†) was observed. A single crystal X-ray diffraction (SC-XRD) analysis using crystals that were obtained from toluene at −78 °C confirms the assignment as [*rac*-(ebthi)Zr(NMe_2_)_2_] (*rac*-3) (Fig. 1). ^13^C NMR analysis shows all typical resonances of the ebthi ligand system and a resonance at *δ* 48.5 ppm for the NMe_2_ ligand which is in line with the value of *δ* 51.6 ppm found for complex 2 (Fig. S5†).

We next investigated the reaction of the *meso* isomer [*meso*-(ebthi)ZrCl_2_] with two equivalents of LiNMe_2_ in Et_2_O at room temperature, aiming at the synthesis of a related bis(amide) complex. To our surprise, only one of the chloride ligands was replaced under these conditions, as evidenced by the ^1^H NMR analysis, which shows resonances at *δ* 6.06, 5.38 and 2.82 ppm in a ratio of 2 : 2 : 6 (Fig. S12†). 2D NMR analysis revealed that all signals show correlation, confirming the presence of a single new species (Fig. S14 and S15†). Crystals of the highly air and moisture sensitive compound suitable for an SC-XRD analysis were obtained from benzene solution at room temperature and confirm the proposed structure of [*meso*-(ebthi)Zr(Cl)(NMe_2_)] (*meso*-4) (Fig. 1).

We next varied the reaction conditions to investigate the direct synthesis of a *meso*-(ebthi) bis(amide) complex. By varying the temperature to 60 °C in Et_2_O formation of bis(amide) compound *meso*-3 could be observed along with three new resonances at *δ* 3.01, 2.97 and 2.93 ppm in a ratio of 6 : 12 : 6. As the resonance at *δ* 2.97 ppm resembles that of the *rac*-(ebthi) complex *rac*-3 isomerisation of the metallocene scaffold is possible. Addition of further four equivalents of LiNMe_2_ results in full conversion of *meso*-4 to furnish the desired bis(amide) complex [*meso*-(ebthi)Zr(NMe_2_)_2_] *meso*-3 in 80% NMR yield along with the *rac*-(ebthi) bis(amide) *rac*-3 (Fig. S7†). To optimise the synthesis conditions of *meso*-3 and to avoid the formation of *rac-*3, we next investigated the influence of different solvents. In aprotic non-coordinating solvents at −40 °C, the reaction was rather unselective and only mixtures containing both isomers could be obtained (Fig. S7†). In benzene, only a complex reaction mixture resulted, from which only a dark yellow oil could be isolated (Fig. S8†). Use of pentane for work up did not result in the formation of defined products (Fig. S9†). During work up in toluene with subsequent crystallisation from toluene/CH_2_Cl_2_ at −78 °C only the isomeric mixture could be isolated which converts to the racemic complex *rac-*3 as the main product at room temperature (Fig. S7†). Every route to separate the *meso* complex *meso*-3 only leads to the formation of the thermodynamically preferred *rac* complex *rac-*3 (Δ_R_*H*^*θ*^(*meso vs. rac*) = −18.5, Δ_R_*G*^*θ*^(*meso vs. rac*) = −14.8 kJ mol^−1^)^52^ probably *via* ring-slippage of an indenyl unit.^53^ To investigate the steric hindrance in the *meso* compared to the *rac* ligand system a buried volume analysis was performed.^54,55^ The spheric sterical hindrance map shows that the global sterical demand is identical for *meso*-3 (buried volume *V*_bur_ = 54.9%) as for *rac*-3 (*V*_bur_ = 55.0%, see ESI†). If the analysed space is decomposed into four equally sized quadrants (NE/SE/SW/NW), the main differences between the two ligand systems become apparent, as expected. The distribution of the space requirement values of *V*_bur.quad._ = 47.1/62.9/47.1/62.9 (*rac-*3) and 45.2/47.4/63.0/64.1 (*meso*-3) agree with *C*_2_ and *C*_S_ symmetries of the metallocene scaffolds. Theoretically, *meso*-4 could exist in two isomeric forms that differ in the position of the chloride and amide ligands relative to the metallocene unit (N_in_–Cl_out_ and N_out_–Cl_in_). As expected, the space requirement analysis of the two quadrants facing away from the metallocene pocket clearly shows the larger space requirement of the amide ligand which can be satisfied in the case of formation of the N_out_–Cl_in_ isomer [N_out_: V_bur(NE/SE)_ = 42.6/44.8%, Cl_out_: V_bur(NE/SE)_ 38.2/38.4%, see ESI†]. This was confirmed experimentally as only the thermodynamically preferred isomer N_out_–Cl_in_ (Δ_R_*H*^*θ*^(N_in_–Cl_out_*vs.* N_out_–Cl_in_) = −33.8, Δ_R_*G*^*θ*^(N_in_–Cl_out_*vs.* N_out_–Cl_in_) = −32.9 kJ mol^−1^) is present in the crystal. In consequence the chloride ligand in *meso*-4 is sterically protected in the *meso*-(ebthi) pocket which explains the necessity of higher temperatures and larger amounts of LiNMe_2_ for formation of complex *meso*-3. Notably, attempts to prepare a dinuclear zirconocene amide complex from [(*meso*-(ebthi)ZrCl)_2_(μ-Me_3_SiC_3_SiMe_3_)]^56^ also led to formation of the mononuclear complex *meso*-3 in low yield (Fig. S6†).

We next examined the selective formation of the mixed chloride/amide complex [*rac*-(ebthi)Zr(Cl)(NMe_2_)] *rac*-4 by addition of one equivalent of LiNMe_2_ to [*rac*-(ebthi)ZrCl_2_] in Et_2_O at room temperature. Selective formation of five new resonances at *δ* 6.16, 6.09, 5.54, 5.12 and 2.87 ppm in a 1 : 1 : 1 : 1 : 6 ratio suggests the formation of the desired mono(amide) species (Fig. S10†). ^13^C NMR analysis shows the characteristic amide resonance at *δ* 48.4 ppm (Fig. S11†). However, it was difficult to separate complex *rac*-4 from simultaneously formed bis(amide) *rac*-3 (9 : 1 ratio, Fig. S10†).

### Catalytic dehydropolymerisation of PhSiH_3_

#### Catalytic studies using complex 2

**Fig. 2:**
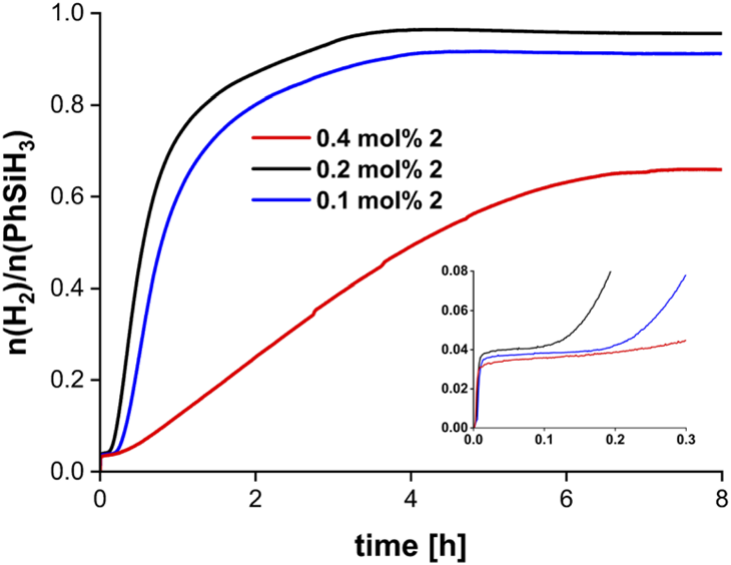
Volumetric curves of dehydropolymerisation of PhSiH_3_ using complex 2. Conditions: room temperature, no solvent, 1.85 mmol PhSiH_3_. The inset shows the induction period that was detected in all catalytic runs (units were removed for clarity and are identical to the full plot).

The dehydropolymerisation of PhSiH_3_ (1.85 mmol) was studied using 0.2 mol% of dinuclear complex 2 without solvent at room temperature in an open reaction vessel with pressure compensation and volumetric monitoring of the reaction system (Scheme 1).^57^ The colour of the reaction mixture changed from bright yellow to dark yellow after addition of PhSiH_3_ and a glassy yellow solid was obtained at maximum conversion of the substrate after 3 h (Fig. 2). According to the amount of evolved hydrogen no full conversion of PhSiH_3_ could be achieved (*n*(H_2_)/*n*_0_(PhSiH_3_) = 0.90). Variation of the catalyst concentration showed that a similar degree of conversion was observed at 0.1 mol%. To our surprise, catalytic runs with 0.4 mol% of 2 gave much lower conversion and the reactions were considerably slower. Reactions of 2 with four equivalents of PhSiH_3_ suggest a complete deactivation and no reaction could be observed under these conditions (Fig. S42†). In all cases an induction period was observed when using 2 (Fig. 2). This behaviour contrasts with dehydropolymerisation reactions of silanes using the mononuclear 1 where no induction period was observed and the conversion increased with higher catalyst concentrations (Fig. S52†). Differences in the activation and/or reaction mechanism must thus be assumed when going from the mononuclear (1) to the dinuclear precatalyst (2). Initial monomerisation of 2 could play a role, however, we have no experimental evidence for this activation step. In fact, cleavage of 2 should result in formation of the alkyne Me_3_SiCH_2_C_2_SiMe_3_, which was not observed by ^1^H NMR spectroscopy.

Control experiments showed that an open reaction setup with pressure compensation and continuous stirring of the reaction solution was required to perform the dehydropolymerisation (Fig. S33†). A significantly decreased activity was found when performing the reaction in solution (Fig. S34†).^58^ To ensure that the catalytic activity of 2 is based on activation and conversion of the silane at the Zr centre we performed NMR scale reactions of PhSiH_3_ with different amine bases. NMR analysis of PhSiH_3_ (1.85 mmol) in the presence of tetramethylethylenediamine (0.37 mmol) or LiNMe_2_ (0.06 mmol) shows no conversion of the substrate (Fig. S43–S48†), indicating that the presence of the metal centre is essential for the dehydropolymerisation of PhSiH_3_ and the basicity of the NMe_2_ group is not sufficient to activate the silane. In line with literature data of reactions using group 4 metal complexes, sterically and electronically different secondary aryl silanes or alkyl silanes such as Ph_2_SiH_2_, Et_2_SiH_2_, or *n*-BuSiH_3_ showed no conversion (Fig. S49, S50 and S51†).^59–61^ When *p*-tolSiH_3_ (*p*-tol = *p*-CH_3_-C_6_H_4_) was used the reaction time increased drastically and only low conversion could be found at room temperature. The formation of minor amounts of polymer was confirmed by ^1^H NMR spectroscopy (Fig. S32†).

#### Catalytic studies using *ansa*-zirconocene amides

We have next investigated the dehydropolymerisation of PhSiH_3_ using the *ansa*-zirconocene amide complexes *rac*/*meso*-3/4 under the same reaction conditions, albeit with a higher concentration of 0.4 mol%, thus providing the same amount of Zr centres per silane as for 2. With complex *rac*-3 H_2_ evolution could be detected by ^1^H NMR spectroscopy and was confirmed by gas chromatography (Fig. S35†). However, the formation of oligomeric or polymeric silanes was neglectable under these reaction conditions or at 60 °C (Fig. S35†). When increasing the catalyst concentration to 0.8 mol% of r*ac*-3 the formation of a mixture of oligomeric silanes could be observed by ^1^H and ^29^Si DEPT NMR spectroscopy at 60 °C after five days. (Fig. S36–S38†). The drastic conditions required as well as the poor reactivity can be rationalised by the increased steric protection of the two amide ligands, which is evident in the buried volume analysis of *rac*-3 with *V*_bur_ 62.9%. As already shown, only the basic properties of the amide ligands do not lead to the conversion of the monomer (*vide infra*). No gas evolution or variations in the viscosity could be observed when using *meso*-4 at room temperature (Fig. S39†), which is in line with a sterically protected chloride ligand placed in the *meso* pocket (*vide infra*).

In contrast immediate H_2_ evolution could be observed in the catalytic reaction using complex *rac*-4 (0.4 mol%) at room temperature with subsequent formation of a highly viscous brownish waxy solid. Volumetric measurement showed a reaction time of three hours with approximately 0.95 eq. of H_2_ evolved before the stirring bar was fixed in the highly viscous polymer (Fig. S53†). Full conversion of PhSiH_3_ could be determined by NMR spectroscopy (Fig. S40†). The significant difference in reactivity between isomeric *meso*-4 and *rac*-4 could also be attributed to the steric protection of the chloride ligand in *meso*-4 (*vide infra*). In consequence, this could result in more facile abstraction of the chloride ligand in *rac*-4, that could lead to highly reactive zirconocene(iii) fragments.^62^ Such species were discussed as an active species in dehydrocoupling of silanes for the less sterically demanding zirconocenes [Cp_2_Zr] and [CpCp*Zr].^25,63^ In summary, both the mono chloride functionality as well as the accessibility of this in *rac*-4 could contribute to the higher activity of this *ansa* zirconocene.

### Molecular weight of polysilanes

The molecular weights of the obtained polymers were determined by size exclusion chromatography (SEC) with calibration using polystyrene as standard. Polymer samples were isolated as glassy yellow solids, soluble in most organic solvents such as benzene or THF and analysed by SEC and ^29^Si NMR spectroscopy (*vide infra*). The molecular weights of polymers isolated from dehydropolymerisation reactions with 2 as catalyst ranged from *M*_n_ = 2100–3000 g mol^−1^, depending on the catalyst concentration (Table 1). SEC profiles of the polymers show minor tailing in the low molecular weight region (Fig. S63–S65†). Formation of low molecular weight cyclic byproducts in combination with linear polysilanes and the subsequent separation was described before for related systems and could serve as an explanation for this observation.^12–14,23,29,64^ The exact linear-to-cyclic rations (L/C) were determined by SEC and from direct integration of ^1^H NMR signals corresponding to Si–H fragments in linear (*δ* 4.3–4.9 ppm) and cyclic (*δ* 4.9–5.3 ppm) structures (Table 1).^23,65,66^

**Table 1:** Summary of SEC analysis of poly(phenylsilanes)

[Zr]	*c* [mol%]	*M*_n_ [g mol^−1^]	*M*_w_ [g mol^−1^]	PDI	L/C^a^
1	0.4	2650	3935	1.46	83 : 17
	0.8	2780	4690	1.68	88 : 12
2	0.1	2000–2200	3160	1.50	85 : 15
	0.2	2850–3000	5637	1.97	89 : 11
	0.4	2785–2910	5082	1.74	83 : 17
*rac-*4	0.4	2600	3200	1.22	85 : 15

a Linear-to-cyclic ratios (L/C) were determined by ^1^H NMR spectroscopy.

An increase in catalyst concentrations results in the formation of higher molecular weight polymers, which is in line with previously reported results using the mononuclear complex 1 (Table 1, Fig. S61 and S62†).^34,67^ Moreover, higher catalyst concentration leads to longer reaction times and a decrease in conversion (Fig. 2, *vide supra*). Thus, catalyst concentrations of 0.2 mol% were found to be optimal with respect to monomer conversion and molecular weight distribution. Polydispersity indices (PDI) are in the range of 1.22–1.97, suggesting the presence of relatively well-defined polysilanes with narrow mass distribution (Fig. S61–S65†).

We have next analysed the kinetics of polymer growth. Therefore, we quenched the dehydropolymerisation reactions (0.2 mol% 2) at different stages of conversion by addition of THF, which inhibits the reaction. SEC analysis of the reaction mixture after 60 minutes shows a broad low intensity peak corresponding to molecular weights in the range *M*_n_ = 150–400 g mol^−1^ (Table 2, Fig. S67†). After 90 minutes a significant increase in *M*_n_ = 1300–1500 g mol^−1^ was found (Table 2, Fig. S68†). Molecular weights determined at full conversion (*t* = 3 h) correspond to the values given in Table 2 (Fig. S69†). The presence of low molecular weight oligomers at early stages of the reaction and the significant increase in *M*_n_ after 60 min indicates that a chain growth mechanism is not likely for the dehydropolymerisation of PhSiH_3_ with 2. Instead, a step growth mechanism could operate. The same scenario could play a role for complex 1, which showed *M*_n_ = 200–400 g mol^−1^ when measured after 5 minutes (Table 2).

**Table 2:** Conversion *vs. M*_n_ analysis

[Zr]	*c* [mol%]	*M*_n_ [g mol^−1^]	*M*_w_ [g mol^−1^]	PDI	Time [min]
1	0.4	200	400	2.02	5
		2685	3935	1.46	20
	0.2	140–400	240–400	1.70	60
2		1340–1550	2850–3000	2.00	90
		2900–3000	5600	1.95	180

When performing the dehydropolymerisation with higher catalyst concentrations we could observe that the catalyst was still present in the polymeric mixture (Fig. S22†). We have therefore added a second portion of PhSiH_3_ (1.85 mmol) after a first catalytic run (0.4 mol% 2) and found that this leads to further conversion to glassy yellow polymer (Fig. S25†). ^1^H NMR analysis of the isolated polymer using 0.4 mol% 2 showed that the catalyst was still intact after further monomer addition (Fig. S25–S27†). The addition of a second portion of monomer does not lead to an increase in molecular weight (*M*_n_ = 2500–2600 g mol^−1^, Fig. S66†), indicating that although the Zr catalyst is still present, reactive polysilane chain ends are not available for further polymer growth.

SEC analysis of polymer fractions obtained with 0.8 mol% *rac*-3 at 60 °C shows only the formation of low molecular weight oligomers (*M*_n_ = 200–450 g mol^−1^) (Fig. S70†). Polymer isolated using 0.4 mol% *rac*-4 as catalyst showed two SEC signals (2100–2300 and 260–400 g mol^−1^, Fig. S71†). Compared to 2 low molecular weight fragments dominate, suggesting the formation of cyclic/linear oligomers (Fig. S71†).

### Microstructure of isolated polysilanes

**Fig. 3:**
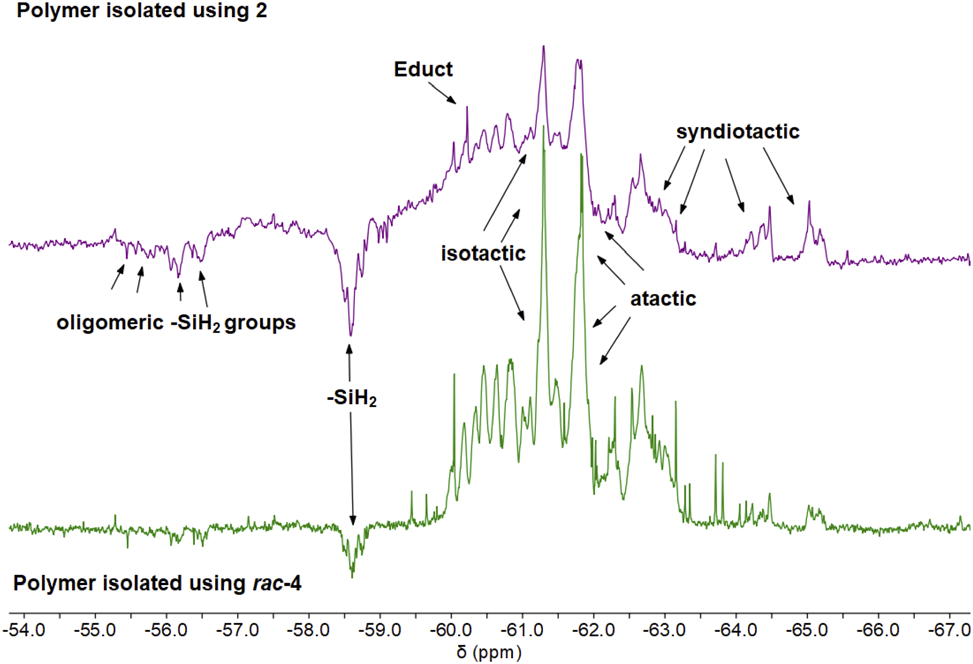
Stacked ^29^Si DEPT135 NMR spectra of poly(phenylsilane) obtained from catalytic dehydropolymerisation of PhSiH_3_ using 0.2 mol% 2 (top), 0.4 mol% *rac*-4 (bottom).

The microstructures of isolated polymers were analysed by ^29^Si DEPT45 and DEPT135 NMR spectroscopy. Polymers that were isolated from reactions using 0.1–0.4 mol% 2 show a complex pattern of resonances in the ^29^Si DEPT spectra (Fig. S16–S24†). Broad signals in the range of *δ* −55 to −65 ppm correspond to the typical, mainly atactic structure of the polymer mixture (Fig. 3).^29,68^^29^Si DEPT135 analysis shows that signals from *δ* −60 to −65 ppm correspond to tertiary Si_*n*_(Ph-SiH)Si_*n*_ units (Fig. 3).^23,29,65^ The DEPT135 experiment also confirmed the phase-shifted resonances at *δ* −56 to −58 ppm as –SiH_2_ end groups (Fig. 3).^29^ Polymer samples synthesised with 0.2 mol% of 2 showed the most well-defined resonance patterns, which can be divided into a high-intensity region from *δ* −60 to −63 ppm and a lower-intensity region at *δ* −63 to −65 ppm (Fig. S21†),^62,68^ the former corresponding to β silyl centres.^6^ Polymer fractions obtained from reactions using 0.1 and 0.4 mol% 2 show a significantly higher portion of linear/cyclic oligomers in the range *δ* −50 to −55 ppm (Fig. S17, S18, S23 and S24†).^62^ In the case of 0.1 mol% 2, this could be the explanation for the lower molecular weight (Table 1, Fig. S17and S18†).

Investigations of polymer microstructure have been done before using deconvolution processes.^6,29,68^ Tertiary Si_*n*_(Ph-SiH)Si_*n*_ units can be categorised into stereospecific triads, namely linear isotatic (mm), linear atactic (mr) and linear syndiotactic polysilane (rr) (Fig. 3).^29^ Although reasonably good separation of the ^29^Si DEPT resonances was observed for samples prepared with 0.2 mol% 2 (Fig. 3), the low resolution and the overlap of resonances made it impossible to analyse the tacticity of the polymer on the basis of these NMR spectra (Fig. S16–S24†).

^29^Si DEPT NMR analysis of polymers obtained from reactions using *ansa*-zirconocene amide complex *rac*-3 suggests the presence of an oligomeric mixture with a complex resonance pattern (Fig. S36–S38†). Formation of the products of PhSiH_3_ dimerisation (*δ* −61.7 ppm, SiH_2_), trimerisation (*δ* −59.1, SiH and −68.5 ppm, SiH_2_), and tetramerisation (two isomers, two sets of resonances at *δ* −59.0, SiH and −65.0 to −65.5 ppm, SiH_2_) could be detected (Fig. S36–S38†).^25^ The formation of higher oligomers can also be assumed, however the separation of the ^29^Si DEPT resonances is not sufficient for a detailed assignment (Fig. S37 and S38†). These results are consistent with the previously reported formation of two diastereomers of the tetramer, showing that the coupling of the Si–Si bonds proceeds without stereoselectivity.^25^^29^Si DEPT spectra of samples produced with 0.4 mol% *rac*-4 show the formation of isotatic and atactic polymer fractions with sharp resonances in the range of *δ* −61.0 ppm (Fig. 3, S40, S41†). Compared to materials prepared with 2, the distribution of oligomers and polymers was better resolved in the range upfield of *δ* −61.0 ppm (Fig. 3, S41†). This result is consistent with SEC data indicating a larger amount of oligomers isolated using *rac*-4 (Fig. S71,†*vide infra*). The *C*_2_ chirality of the ebthi ligand system has a significant effect on the tacticity and the formation of low molecular weight oligomers, as previously shown in the literature.^25^

## Conclusion

The dinuclear zirconocene complex 2 shows selectivity for the dehydropolymerisation of phenylsilane that is comparable to that of the parent mononuclear analog Cp_2_Zr(NMe_2_)_2_, producing a polymer that possesses isotactic, atactic and syndiotactic structures. The molecular weights of the isolated polyphenylsilane products are comparable to those prepared by previously studied related catalyst systems, which again shows the previously discussed limitations of this approach of silane dehydropolymerisation. SEC analysis of the polymers at varying conversion suggests step growth as a likely scenario for polymer formation. In contrast to previously reported highly active mononuclear complex Cp_2_Zr(NMe_2_)_2_, no decomposition of the dinuclear catalyst 2 was observed, and the catalyst retains its activity at high conversion of substrate. As a consequence, we could show that selective polymer formation with a well-defined mass distribution can be observed after addition of further monomer. *Ansa*-zirconocene amide complexes prepared in this study were tested as catalysts and the activity of these species was found to be strongly dependent on the symmetry of the metallocene fragment and the presence of amide/chloride ligands. Only the *C*_2_ symmetric mixed amide/chloride complex *rac*-4 showed activity that was comparable to that of dinuclear 2. Future studies will aim at evaluation of the potential of the herein reported and further dinuclear group 4 metallocene complexes for dehydrocoupling reactions.

## Author contributions

K. L., F. R. and T. B. conceived and conceptualised the project. K. L. performed the experiments and analysed the data. A. S. performed the SC-XRD and F. R. the DFT study. F. R. and T. B. supervised the project. T. B. acquired funding. K. L., F. R. and T. B. prepared and revised the manuscript.

## Conflicts of interest

There are no conflicts to declare.

## Supplementary Material

RA-012-D2RA04955D-s001

RA-012-D2RA04955D-s002
