# Supplementary material for: Mono- and dinuclear zirconocene(iv) amide complexes for the catalytic dehydropolymerisation of phenylsilane
Source: RSC Adv. 2022 Sep 15;12(40):26277–83. doi: 10.1039/d2ra04955d (PMC9477068; doi:10.1039/d2ra04955d)
Supplement: RA-012-D2RA04955D-s001 [file RA-012-D2RA04955D-s001.pdf]

Electronic Supplementary Material (ESI) for RSC Advances.
This journal is © The Royal Society of Chemistry 2022

SUPPORTING INFORMATION

Zirconocene amide complex catalysed dehydropolymerisation of phenylsilane: new catalysts and mechanistic insights

Kevin Lindenau, Anke Spannenberg, Fabian Reiß* and Torsten Beweries*

Leibniz-Institut für Katalyse e.V., Albert-Einstein-Str. 29a, 18059 Rostock, Germany.

Table of contents

1. Experimental Details....................2
2. NMR spectroscopy....................5
3. Volumetric studies....................32
4. Crystallographic details....................33
5. Vibrational spectroscopy....................35
6. SEC analysis....................39
7. Computational details....................45
8. References....................51

1. Experimental Details

1.1. General

All manipulations were carried out in an oxygen- and moisture-free argon atmosphere using standard Schlenk and drybox techniques. The solvents were purified with the Grubbs-type column system "Pure Solv MD-5" and dispensed into thick-walled glass Schlenk bombs equipped with Young-type Teflon valve stopcocks. $PhSiH_3$, Ph_2SiH_2, Et_2SiH_2 and *n*-$BuSiH_3$ were purchased from Sigma-Aldrich. $PhSiH_3$ and $Cp_2Zr(NMe_2)_2$ were synthesised according to literature procedures.[1 2]

NMR spectra were recorded on Bruker AV300 and AV400 spectrometers. ^{1}H and ^{13}C chemical shifts were referenced to the solvent signal: benzene-d_6 (δ_H = 7.16 ppm, δ_C = 128.06 ppm), toluene-d_8 (δ_H = 2.08 ppm, δ_C = 20.43 ppm), THF-d_8 (δ_H = 3.58 ppm, δ_C = 25.3 ppm).[3]

Raman spectra were recorded on a LabRAM HR 800 Raman Horiba spectrometer equipped with an Olympus BX41 microscope with variable lenses. The samples were excited by different laser sources: 633 nm (17 mW, air cooled), 784 nm Laser diode (100 mW, air-cooled) or 473 nm Ar+ Laser (20 mW, air-cooled). All measurements were carried out at ambient temperature.

IR spectra were recorded on a Bruker Alpha FT-IR, ATR Spectrometer, spectra are not corrected.

MS analysis was done using a Finnigan MAT 95-XP instrument (Thermo-Electron) in CI^+/CI^- mode (isobutene) and for the air stable compounds in EI mode.

CHN analysis was done using a Leco TruSpec elemental analyser. At this point it should be pointed out that in the case of the bimetallic zirconocene complexes we could not obtain satisfactory elemental analysis in most cases. Despite repeated recrystallisation, repeated measurements with and without oxidiser V_2O_5 and modified furnace temperature, we observed up to 20% less carbon content than calculated/expected. This behaviour might be explained by formation of mixed zircon-silicon-carbides (ceramics) in the furnace and therefore the carbon content dramatically decreases.[4]

Melting points are uncorrected and were determined in sealed capillaries under Ar atmosphere using a Mettler-Toledo MP 70.

Chloride analysis was done using a Radiometer Analytical SAS TitraLab 870 TIM 870. Titration was performed with a turning point titration using a MC6091Ag combination electrode in a range of ± 2000 mV and a resolution of 0.1 mV. The exploration were carried out by oxygen oxidation of Schöniger followed by Mohr titration.[56]

X-Ray diffraction data were collected on a Bruker Kappa APEX II Duo diffractometer. The structures were solved by direct methods (SHELXS-97)[7] and refined by full-matrix least-squares procedures on F^2 (SHELXL-2018).[8] Diamond[9] was used for graphical representations.

All DFT calculations were carried out with the Gaussian 16 package of molecular orbital programs.

1.2. General procedure of dehydropolymerisation experiments

A mixture of the catalyst (0.01 mmol, 0.2 mol%) and $PhSiH_3$ (0.23 mL, 6.47 mmol) in a Schlenk tube was stirred (magnetic stirring bar) at the reported temperatures in an open system with pressure compensation. The reaction mixture was stirred for one or more days at room temperature. To obtain an NMR sample, an aliquot of the reaction mixture was added to a dried, argon-filled NMR tube containing C_6D_6. For the GPC sample an aliquot was removed, added to THF and measured immediately.

1.3. Synthesis of 2

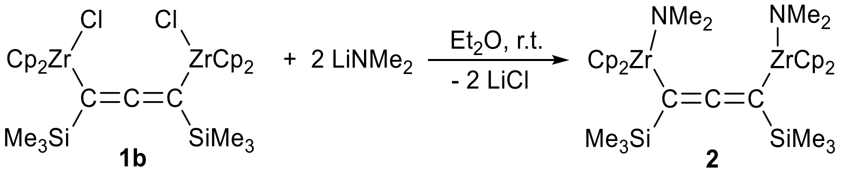

Compound **1b** (400 mg, 0.57 mmol) and $LiNMe_2$ (58.6 mg, 1.15 mmol) were dissolved in 15 mL of Et_2O and the mixture was stirred overnight. The colour of the reaction mixture changed from red to orange and Et_2O was removed in vacuo. The orange solid residue was dissolved in benzene, followed by filtration. After washing with benzene (3x2 mL) the solution was concentrated to obtain orange crystals of **4** (326 mg, 82%). Crystals suitable for X-ray analysis were obtained from a pentane solution.

^{1}H NMR (25 °C, benzene-d_6, 300 MHz): δ 6.05 ppm (s, 10H, *Cp*), 5.99 (s, 10H, *Cp*), 2.86 (s, 12H, *NMe$_2$*), 0.39 (s, $^2J(^1H, ^{29}Si) = 6.2$ Hz, 18H, (Si*CH$_3$*)$_3$. **^{13}C NMR** (25 °C, benzene-d_6, 100 MHz): δ193.6 (C=*C*=C), 111.6 (Cp), 111.3 (Cp), 85.5 (*C*=C=*C*), 51.6 (N(*C*H$_3$)$_2$), 3.2 (Si*C*H$_3$). **^{29}Si-inept NMR** (25 °C, benzene- d_6, 79.49 MHz): δ-3.78; (*Si*Me$_3$). **IR** (ATR, cm^{-1}): ν3095 (w), 2815 (vw) 2766 (vw) 1760 (s, C=C=C), 1230 (m), 937 (m), 787 (s). **Raman** (633nm, 10 sec, 10 scans): ν3116 (vw), 3048 (vw), 2954 (vw), 2899 (vw), 2821 (vw), 2771 (vw), 2158 (vw), 1768 (vw), 1443 (vw), 1367 (vw), 1229 (w), 1130 (s), 1069 (w), 943 (m), 845 (vw), 802 (vw), 674 (vw), 625 (vw), 544 (vw), 534 (vw), 352 (vw), 292 (w), 264 (w) cm^{-1}. **MS-CI$^+$** (*isobutene*): *m/z* (%): 640 (80) [$(Cp_2Zr)_2(Me_3SiC_3SiMe_3NMe_2)$]$^+$, 403 (7) [$Cp_2Zr(Me_3SiC_3SiMe_3)$]$^+$, 263 (15) [$Cp_2ZrNMe_2$]$^+$, 185 (44) [$Me_3SiC_3SiMe_3$]$^+$, 109 (100) [$C_3SiMe_3$]$^+$, 73 (15) [$Si(CH_3)_3$]$^+$. **Mp**: 159 °C (decomp.). **CHN analysis:** calc (%) for $C_{33}H_{50}N_2Si_2Zr_2$: C, 55.5; H, 7.1; N, 3.9. Found: C, 48.9; H, 6.4; N, 2.6 (best value of four measurements).

1.4. Synthesis of *rac*-3

[*rac*-(ebthi)ZrCl$_2$] (527 mg 1.24 mmol) and $LiNMe_2$ (126 mg, 2.47 mmol) were dissolved in 20 mL of Et_2O and the mixture was stirred overnight at room temperature. Et_2O was removed in vacuo. The orange solid residue was dissolved in 20 mL of toluene, followed by filtration. The orange solution was concentrated and stored overnight at -78 °C in toluene to obtain orange crystals of [*rac*-(ebthi)Zr(NMe$_2$)$_2$] (466 mg, 88 %).

^{1}H NMR (25 °C, benzene-d_6, 300 MHz): δ 6.08 (d, $^3J_{H,H} = 2.7$ Hz, 2H, C*H* ebthi), 5.31 (d, $^3J_{H,H} = 2.8$ Hz, 2H, C*H* ebthi), 2.97 (s, 12H, N(C*H*$_3$)$_2$), 2.86-2.77 (m, 2H, C*H*$_2$ ebthi), 2.72-2.64 (m, 2H, C*H*$_2$ ebthi), 2.64-2.55 (m, 2H, C*H*$_2$ ebthi), 2.40-2.24 (m, 3 x 6H, C*H*$_2$ ebthi), 1.82-1.33 ppm (m, 4 x 8H, C*H*$_2$ ebthi). **^{13}C NMR** (25 °C, benzene-d_6, 100 MHz): δ 130.6, 127.4, 116.8 (6 x *C* ebthi), 109.7, 106.0 (4 x *C*H ebthi), 48.5 (4 x *C* N(*C*H$_3$)$_2$), 28.5, 25.7, 24.7, 24.5, 23.2 ppm (10 x *C*H$_2$ ebthi). **IR** (ATR, cm^{-1}): ν 3075 (w), 2933 (w), 2844 (w), 2805 (m), 2756 (m), 1439 (w), 1418 (w), 1233 (w), 1130 (m), 1055 (w), 938 (s), 771 (s), 524 (m), 462 (w). **Raman** (632 nm, 20 sec, 15 scans): ν3096 (w), 2962 (w), 2914 (m), 2857 (m), 2808 (m), 2760

(m), 1456 (w), 1429 (m), 1396 (w), 1275 (vw), 1234 (w), 1208 (vw), 1029 (vw), 950 (w), 855(vw), 712 (w), 527 (vw), 398 (m), 315 (m), 268 (m), 168 (m), 151 (s) cm^{-1}. **MS-CI$^+$** (*isobutene*): *m/z* (%): 443 (100) $[M]^+$, 398 (66) $[C_2H_4(C_{18}H_{20})_2ZrN(CH_3)_2]^+$. **Mp**: 168 °C (decomp.). **CHN analysis:** calc (%) for $C_{24}H_{36}N_2Zr$: C, 65.0; H, 8.2; N, 6.3. Found: C, 64.9; H, 8.4; N, 6.3.

1.5. Synthesis of *meso*-3

[*meso*-(ebthi)$ZrCl_2$] (436 mg, 1.02 mmol) and $LiNMe_2$ (105 mg, 2.04 mmol) were dissolved in 15 mL of Et_2O and the mixture was stirred overnight at 60 °C. The colour of the reaction mixture changed from colourless to yellow and Et_2O was removed in vacuo. The yellow residue was dissolved in 15 mL of toluene and filtered off. After addition of 4 eq of $LiNMe_2$ the yellow solution was stirred over four days at 60 °C to obtain [*meso*-(ebthi)$Zr(NMe_2)_2$].

^{1}H NMR (25 °C, benzene-d_6, 300 MHz): δ 6.15 (d, $^3J_{H,H}$ = 2.9 Hz, 2H, C*H* ebthi), 5.31 (d, $^3J_{H,H}$ = 2.9 Hz, 2H, C*H* ebthi), 3.01 (s, 6H, $N(CH_3)_2$), 2.93 (s, 6H, $N(CH_3)_2$).

1.6. Synthesis of *rac*-4

[*rac*-(ebthi)$ZrCl_2$] (300 mg, 0.70 mmol) and $LiNMe_2$ (36 mg, 0.70 mmol) were dissolved in 10 mL of Et_2O and the mixture was stirred overnight at room temperature. Subsequently Et_2O was removed in vacuo and dried for 3 h. The orange foam was dissolved in 5 mL of benzene and stirred for 15 minutes. Benzene was filtered off and the orange oil was washed with benzene (3x 1 mL). The orange solution was removed in vacuo and the orange oil was dissolved in 3 mL Et_2O. The Et_2O was removed in vacuo to obtain [*rac*-(ebthi)Zr(Cl)(NMe_2)] as an orange solid (226 mg, 75 %).

^{1}H NMR (25 °C, benzene-d_6, 300 MHz): δ 6.16 (d, $^3J_{H,H}$ = 2.9 Hz, 1H, C*H* ebthi), 6.06 (d, $^3J_{H,H}$ = 3.0 Hz, 1H, C*H* ebthi), 5.54 (d, $^3J_{H,H}$ = 3.0 Hz, 1H, C*H* ebthi), 5.11 (d, $^3J_{H,H}$ = 3.0 Hz, 1H, C*H* ebthi), 3.25-3.15 (m, 2H, CH_2 ebthi), 2.87 (s, 6H, NMe_2), 2.80-2.68 (m. 2H, CH_2 ebthi), 2.57-2.45 (m, 4H, 2 x CH_2 ebthi), 2.44-2.30 (m, 4H, 2 x CH_2 ebthi), 2.27-2.14 (m, 4H, 2 x CH_2 ebthi), 1.54-1.39 ppm (m, 4H, 2 x CH_2 ebthi). **^{13}C NMR** (25 °C, benzene-d_6, 100 MHz): δ 133.5, 132.1, 131.0, 123.1, 114.9 (12 x *C* ebthi), 113.4, 111.1, 109.3, 106.4 (8 x *C*H ebthi), 48.4 (2 x *C* $N(CH_3)_2$), 28.8, 28.7, 25.5, 24.8, 24.7, 24.3, 23.3, 22.8 ppm (20 x CH_2 ebthi). **IR** (ATR, cm^{-1}): ν 3065 (m), 2923 (m), 2849 (m), 2811, (m), 2762 (m), 1490 (m), 1435 (m), 1237 (m), 1134 (m), 1059 (w), 940 (s), 802 (s), 783 (s), 690 (s), 530 (m). **Raman** (633 nm, 25 sec, 10 scans): ν3098 (w), 3082 (vw), 3068 (vw), 3048 (vw), 2929 (m), 2887 (w), 2854 (w), 2767 (w), 1457 (w), 1432 (w), 1334 (vw), 1277 (vw), 1238 (m), 944 (m), 681 (w), 535 (w), 314 (m), 148 (m), 91 (m) cm^{-1}. **MS-CI$^+$** (*isobutene*): *m/z* (%):434 (100) $[M]^+$, 398 (71) $[C_2H_4(C_{18}H_{20})_2ZrN(CH_3)_2]^+$. **Mp**: 141 °C (decomp.). **CHN analysis:** calc (%) for $C_{22}H_{30}NClZr$: C, 60.7, H, 7.0. Found: C, 60.6, H, 8.8.

1.7. Synthesis of *meso*-4

[*meso*-(ebthi)$ZrCl_2$] (328 mg 0.77 mmol) and $LiNMe_2$ (80 mg, 1.58 mmol) were dissolved in 15 mL of Et_2O and the mixture was stirred overnight at room temperature. Et_2O was removed in vacuo. The yellow solid residue was dissolved in 15 mL of benzene, followed by filtration. After washing with benzene (3x3 mL) the solution was removed to obtain [*meso*-(ebthi)Zr(Cl)(NMe_2)] as a yellow solid (294 mg, 89%). Crystals suitable for X-ray diffraction analysis were obtained from benzene solution.

^{1}H NMR (25 °C, benzene-d_6, 300 MHz): δ 6.06 (d, $^3J_{H,H}$ = 3.1 Hz, 2H, C*H* ebthi), 5.38 (d, $^3J_{H,H}$ = 3.0 Hz, 2H, C*H* ebthi), 3.12 (m, 2H, CH_2 ebthi), 2.82 (s, 6H, $N(CH_3)_2$) 2.68-2.60 (m, 2H, CH_2 ebthi), 2.53-2.44 (m, 4H, 2 x CH_2 ebthi), 2.40-2.21 (m, 4H, 2 x CH_2 ebthi), 2.19-1.93 (m, 4H, 2 x CH_2 ebthi), 1.54-1.37 ppm (m, 4H, 2 x CH_2 ebthi). **^{13}C NMR** (25 °C, benzene-d_6, 100 MHz): δ 128.8, 127.5, 123.4 (6 x *C* ebthi), 113.4, 102.4 (4 x *C*H ebthi), 49.6 (2 x *C* $N(CH_3)_2$), 27.6, 24.9, 23.3, 23.0, 22.9 ppm (10 x CH_2 ebthi). **IR** (ATR, cm^{-1}): ν 3075 (w), 2919 (m), 2851 (m), 2813 (m), 2766 (m), 1437 (m), 1424 (m), 1235 (m), 1134 (m), 1117 (m), 1057 (m), 942 (s), 791 (s), 653 (w), 532 (m). **Raman** (632 nm, 20 sec, 15 scans): ν 3108 (vw), 3077 (vw), 2932 (m), 2887 (w), 2860 (w), 2817 (vw), 2770 (vw), 1471 (w), 1440(w), 1400 (vw), 1338 (vw), 1295 (vw), 1239 (m), 945 (m), 807 (w), 693 (w), 538 (w), 346 (w), 323 (s), 286 (m), 160 (s), 115 cm^{-1} (m). **MS-CI$^+$** (*isobutene*): *m/z* (%): 434 (100) $[M]^+$, 398 (15) $[C_2H_4(C_{18}H_{20})_2ZrN(CH_3)_2]^+$. **Mp**: 163 °C (decomp.). **CHN analysis:** calc (%) for $C_{22}H_{30}NClZr$: C, 60.7; H, 7.0; N, 3.2. Found: C, 60.4; H, 6.8; N, 2.7 (best value of four measurements).

2. NMR spectroscopy

2.1. NMR spectra of 2

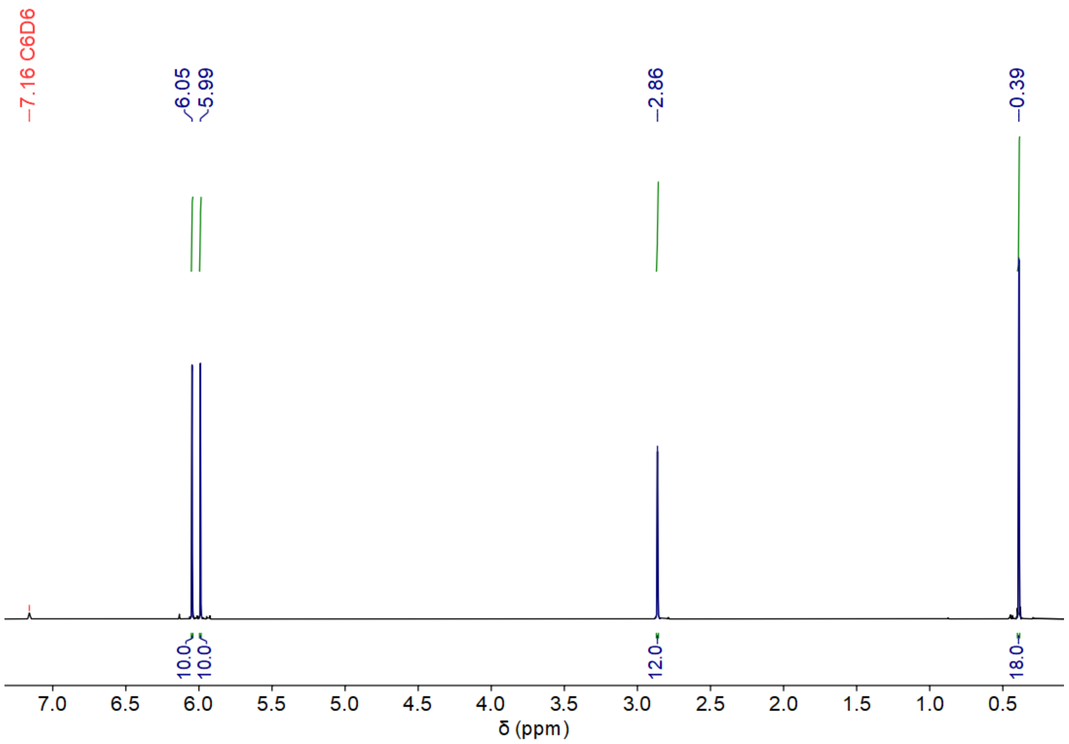

Figure S 1. ^{1}H NMR spectrum of **2** (25 °C, benzene-d_6, 300.13 MHz).

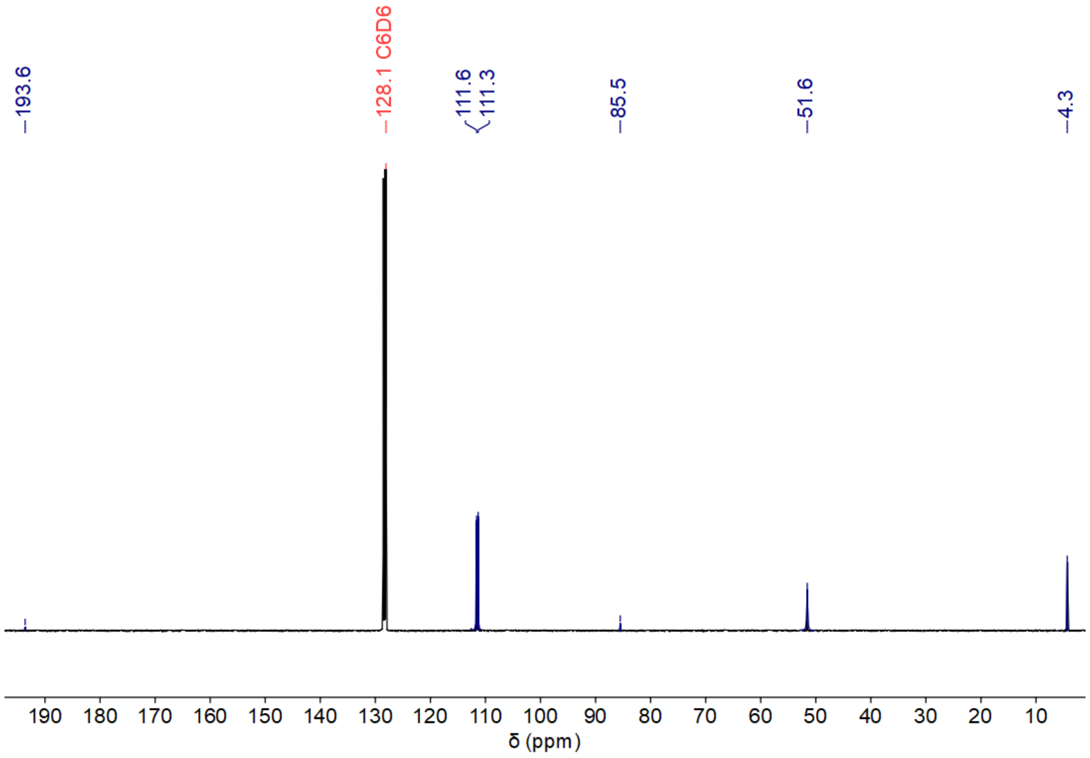

Figure S 2. ^{13}C NMR spectrum of **2** (25 °C, benzene-d_6, 100.63 MHz).

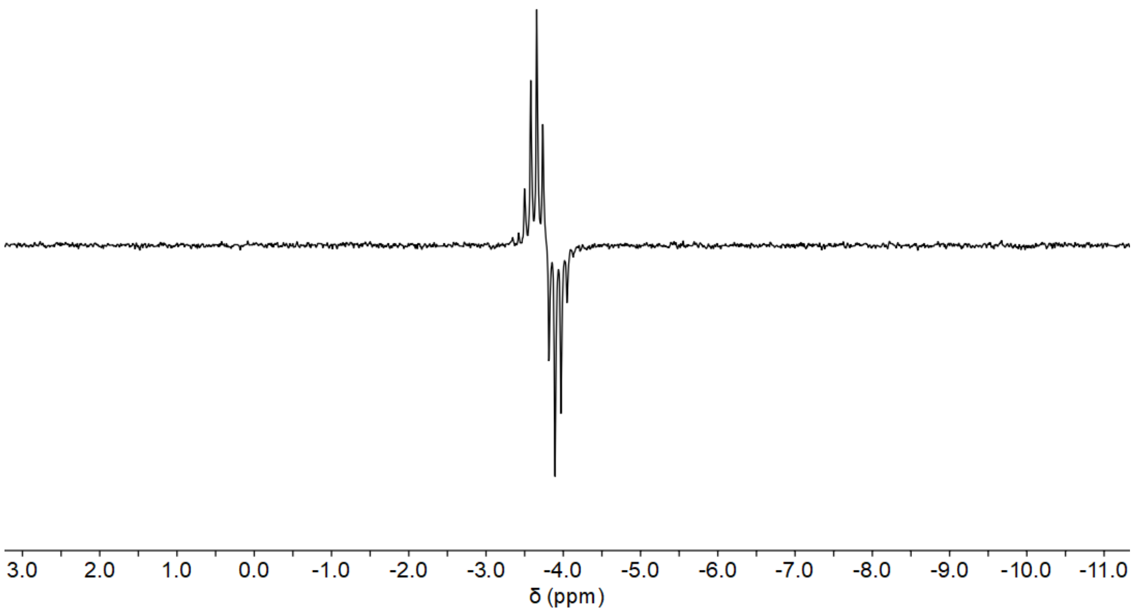

Figure S 3. ^{29}Si INEPT NMR spectrum of **2** (25 °C, benzene-d_6, 79.49 MHz).

2.2. NMR spectra of *rac*-3

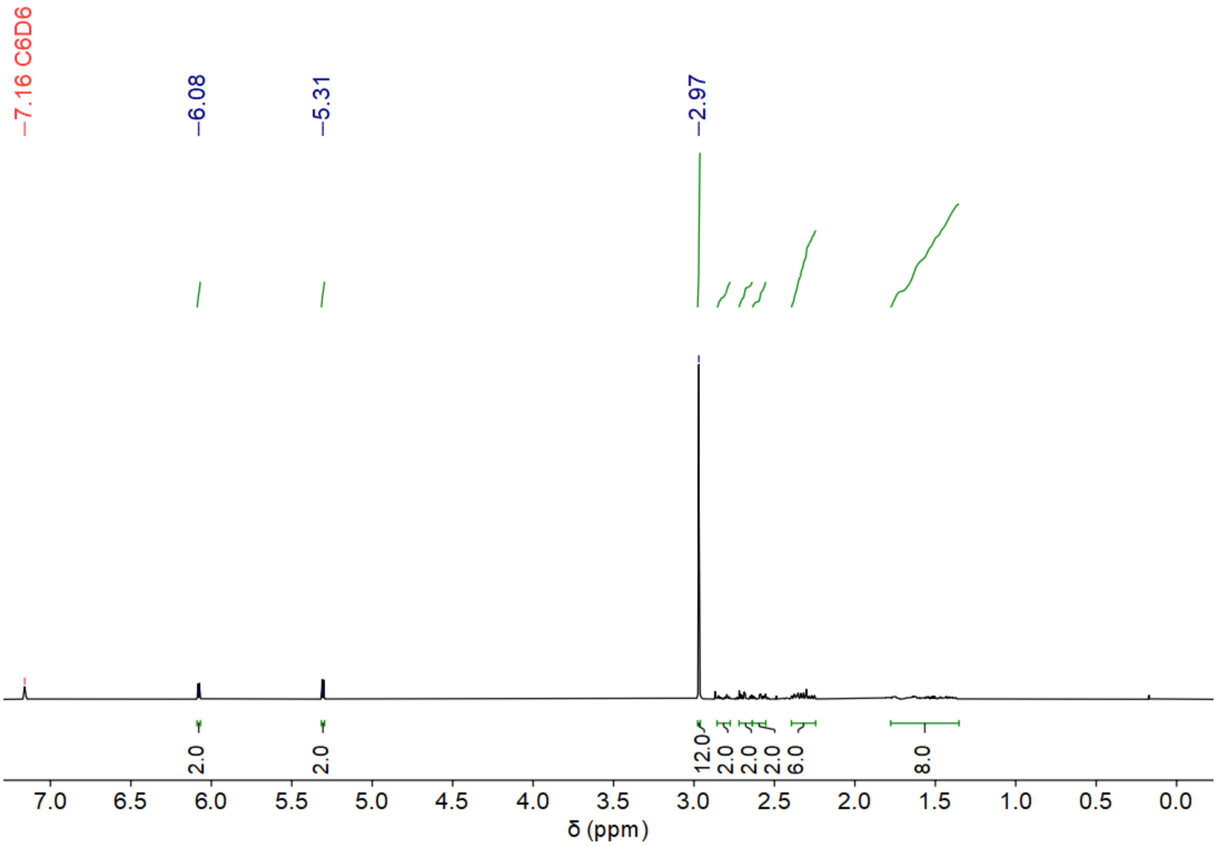

Figure S 4. ^{1}H NMR spectrum of ***rac*-3** (25 °C, benzene-d_6, 300.20 MHz).

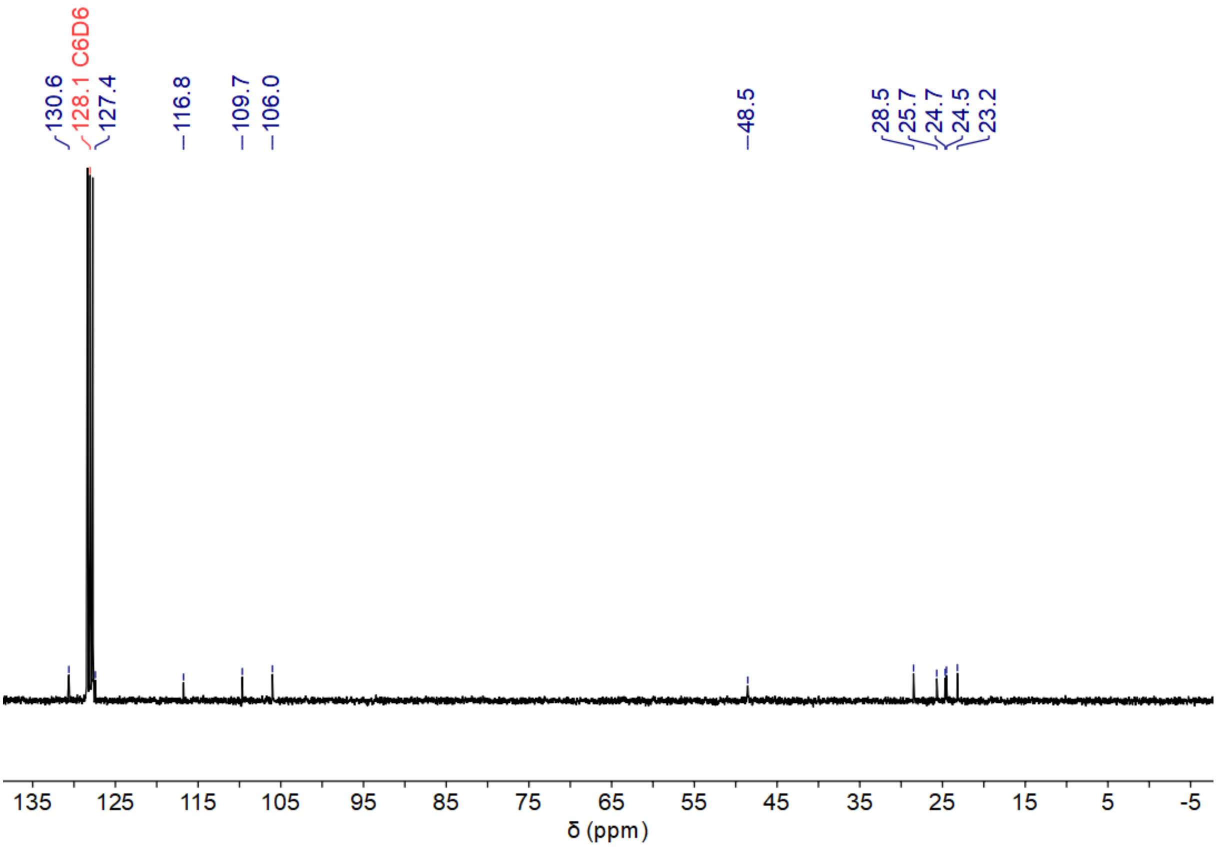

Figure S 5. ^{13}C NMR spectrum of ***rac*-3** (25 °C, benzene-d_6, 75.49 MHz).

2.3. NMR spectra of *meso*-3

2.3.1. NMR spectrum synthesised from [(*meso*-(ebthi)Zr(Cl_2)(μ-C_3)(NMe_2)$_2$]

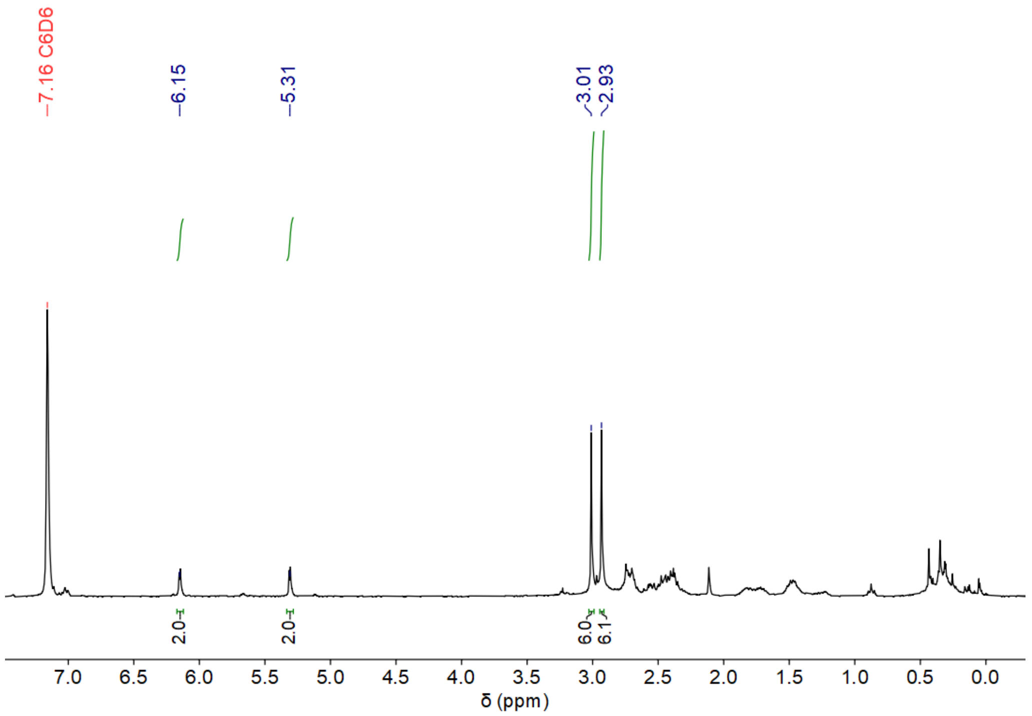

Figure S 6. ^{1}H NMR spectrum of ***meso*-3** (25 °C, benzene-d_6, 300.20 MHz).

2.3.2. NMR spectrum synthesized from [*meso*-(ebthi)ZrCl_2]

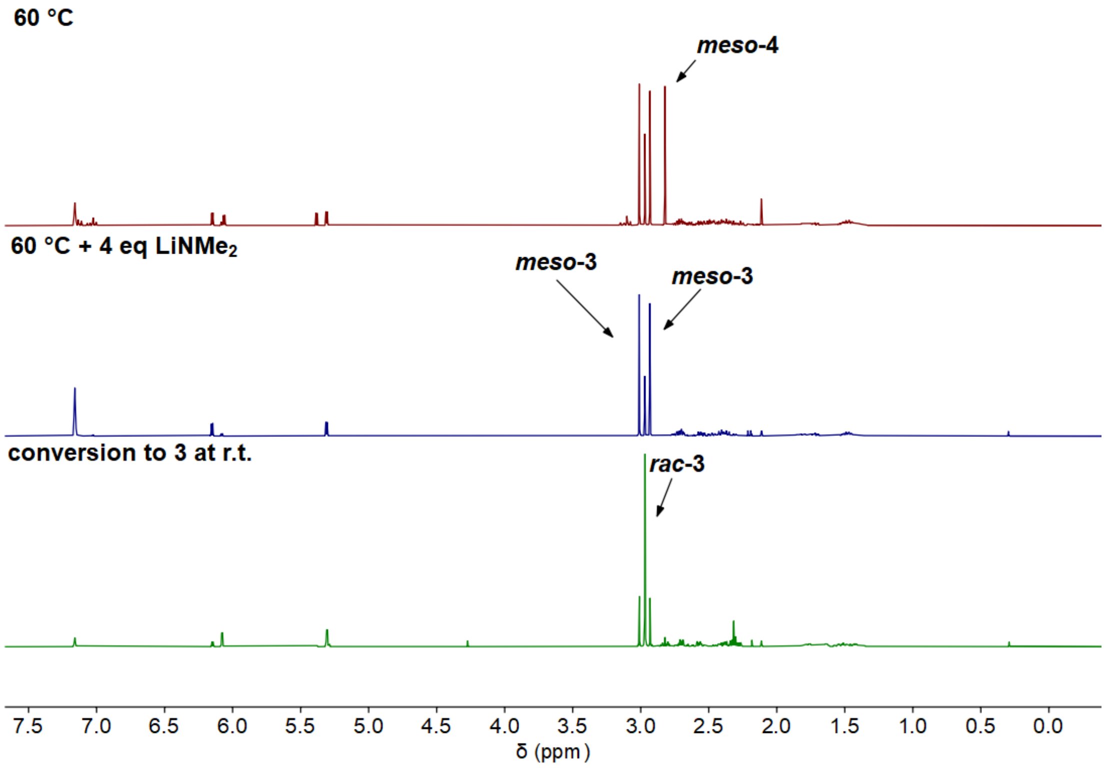

S

Figure S 7. ^{1}H NMR spectra (25 °C, benzene-d_6, 300.20 MHz) of the reaction of [*meso*-(ebthi)ZrCl_2] with LiNMe_2. Conditions: 60 °C, diethyl ether, 1.02 mmol [*meso*-(ebthi)ZrCl_2], 2.04 mmol LiNMe_2 (red: reaction at 60 °C overnight, blue: additional 4 eq LiNMe_2 and stirring 24h, green: conversion to ***rac*-3** in toluene/CH_2Cl_2 at room temperature).

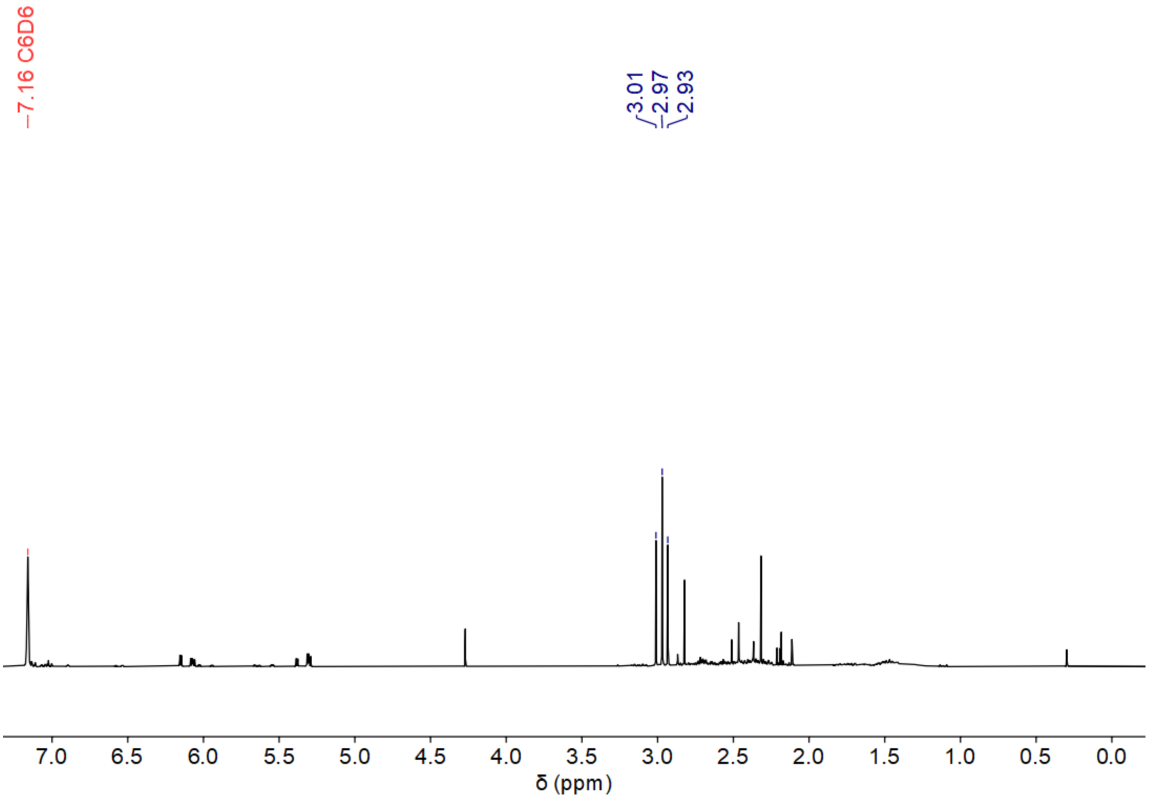

Figure S 8. ^{1}H NMR spectrum of the reaction of [*meso*-(ebthi)$ZrCl_2$] with $LiNMe_2$. Conditions: 60 °C, benzene, 0.70 mmol [*meso*-(ebthi)$ZrCl_2$], 2.80 mmol $LiNMe_2$ (25 °C, benzene-d_6, 300.20 MHz).

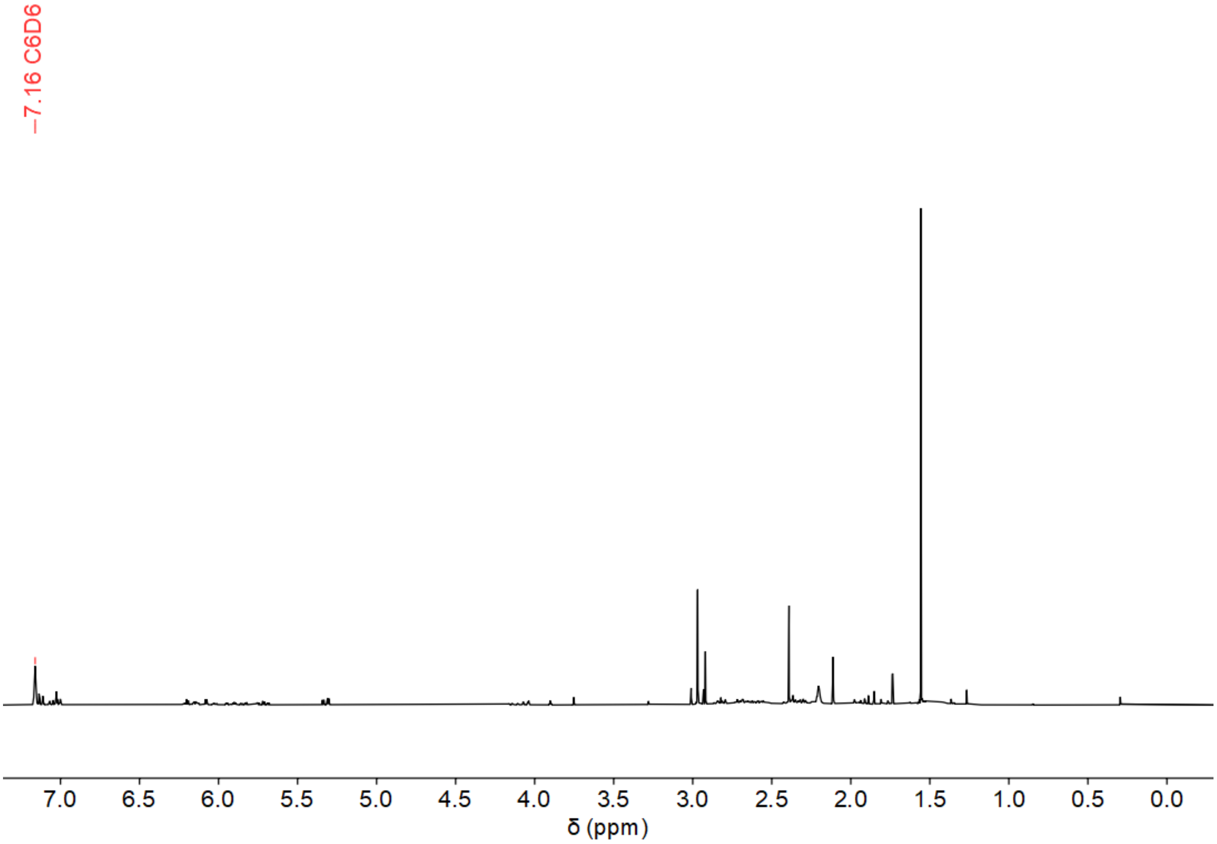

Figure S 9. ^{1}H NMR spectrum of the reaction of [*meso*-(ebthi)$ZrCl_2$] with $LiNMe_2$. Conditions: 60 °C, pentane, 0.70 mmol [*meso*-(ebthi)$ZrCl_2$], 2.80 mmol $LiNMe_2$ (25 °C, benzene-d_6, 300.20 MHz).

2.4. NMR spectra of rac-4

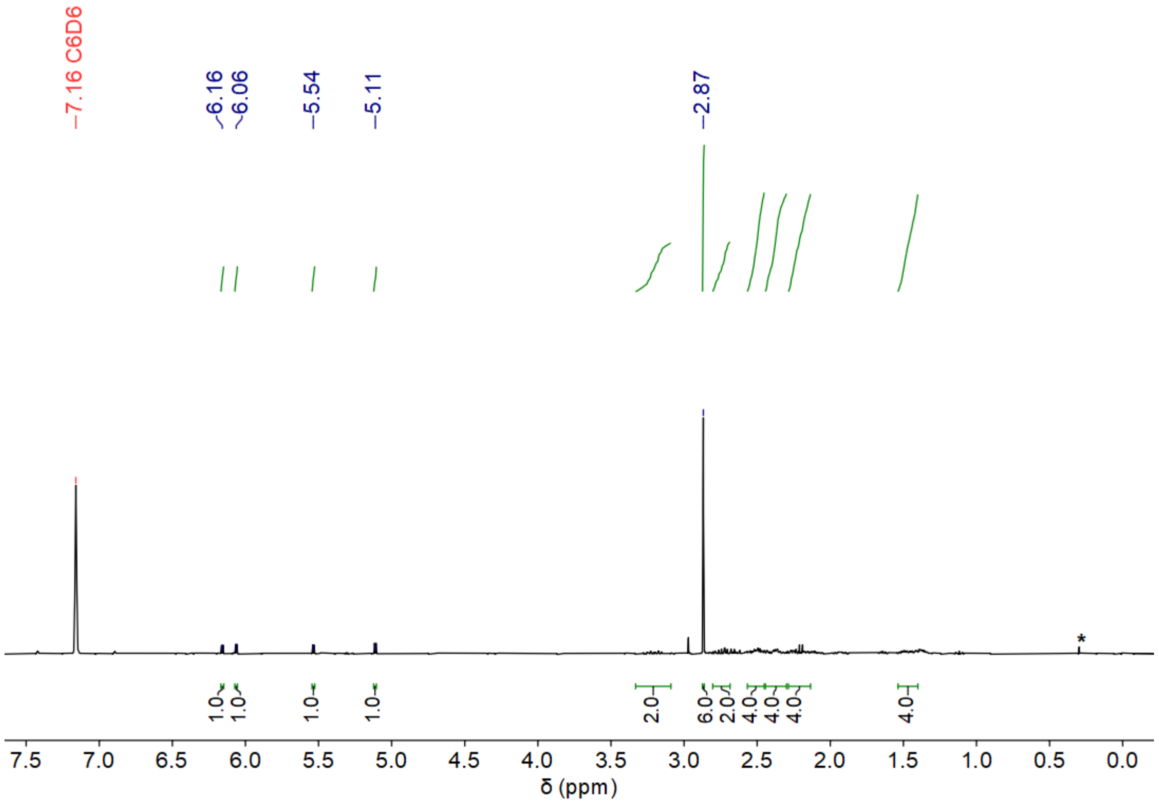

Figure S 10. ^{1}H NMR spectrum of ***rac*-4**, asterisk marks silicon grease (25 °C, benzene-d_6, 400.13 MHz).

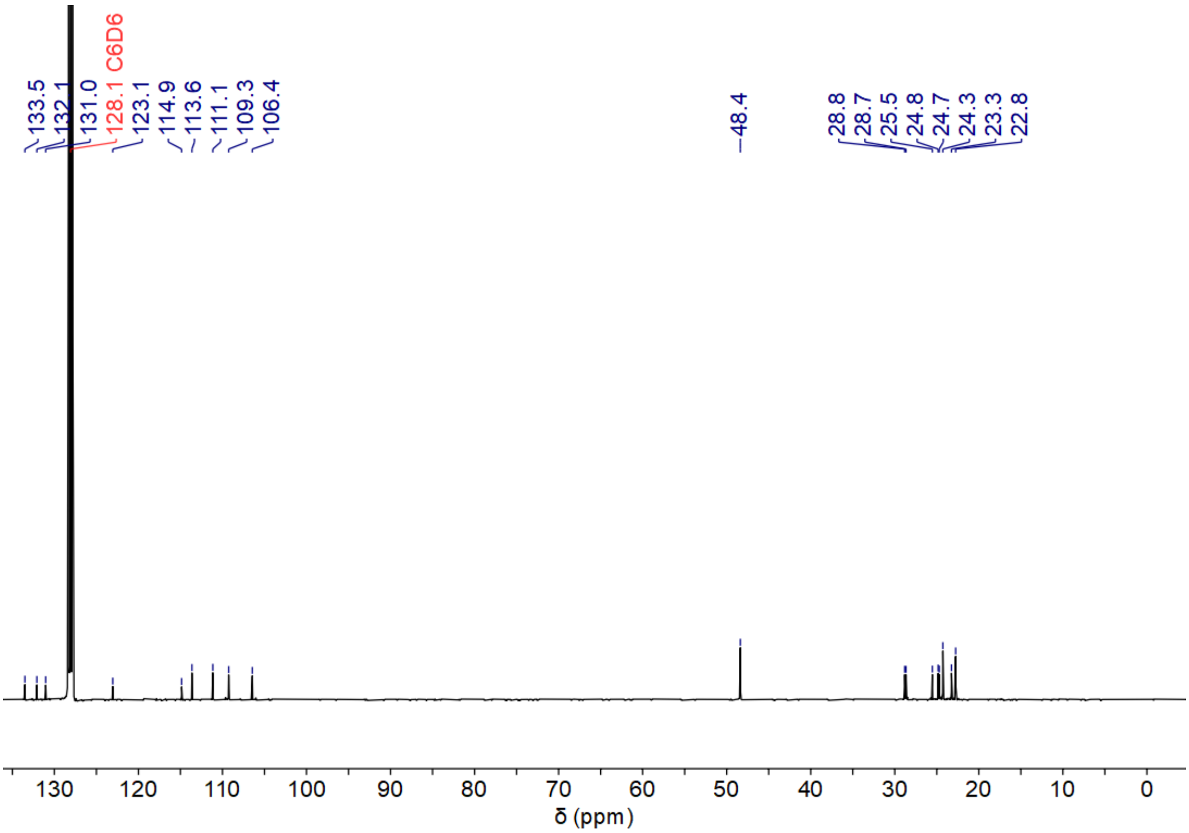

Figure S 11. ^{13}C NMR spectrum of ***rac*-4** (25 °C, benzene-d_6, 75.49 MHz).

2.5. NMR spectra of *meso*-4

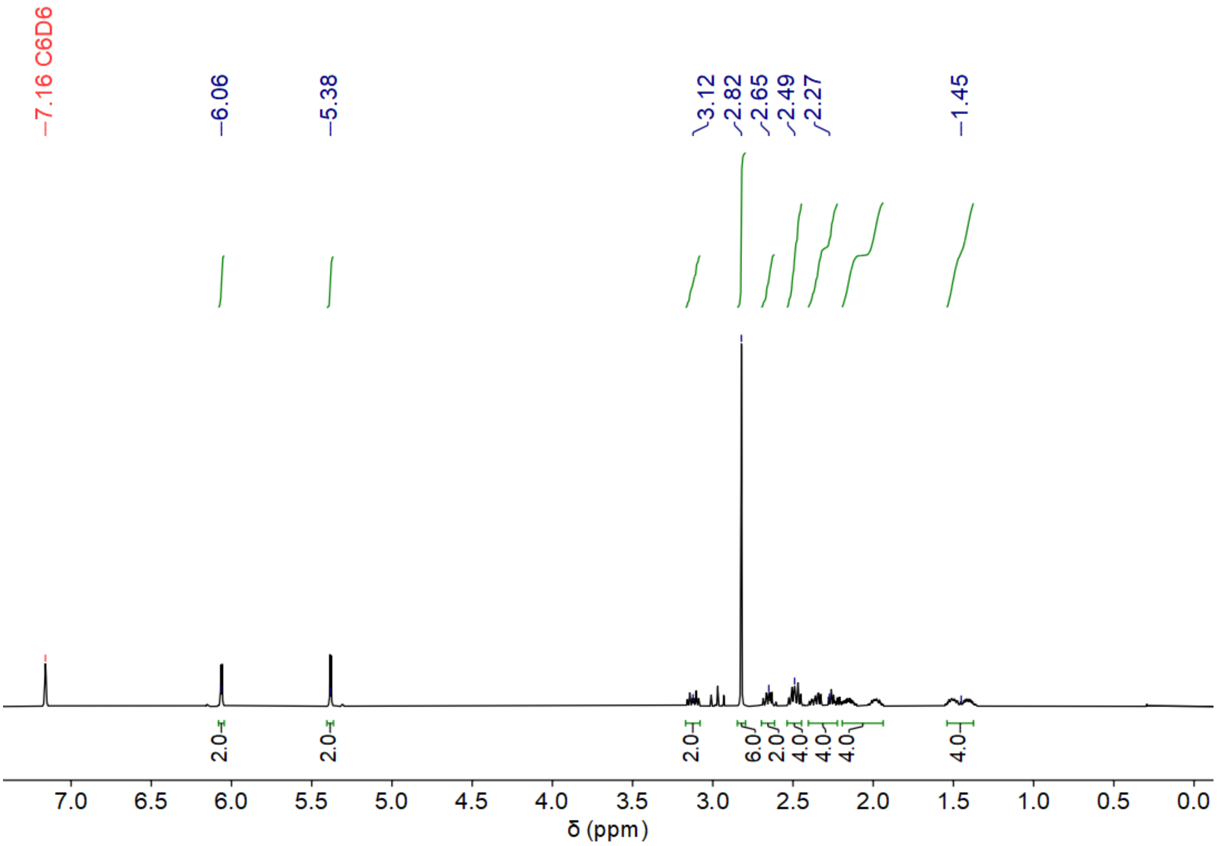

Figure S 12. ^{1}H NMR spectrum of ***meso*-4** (25 °C, benzene-d_6, 400.13 MHz).

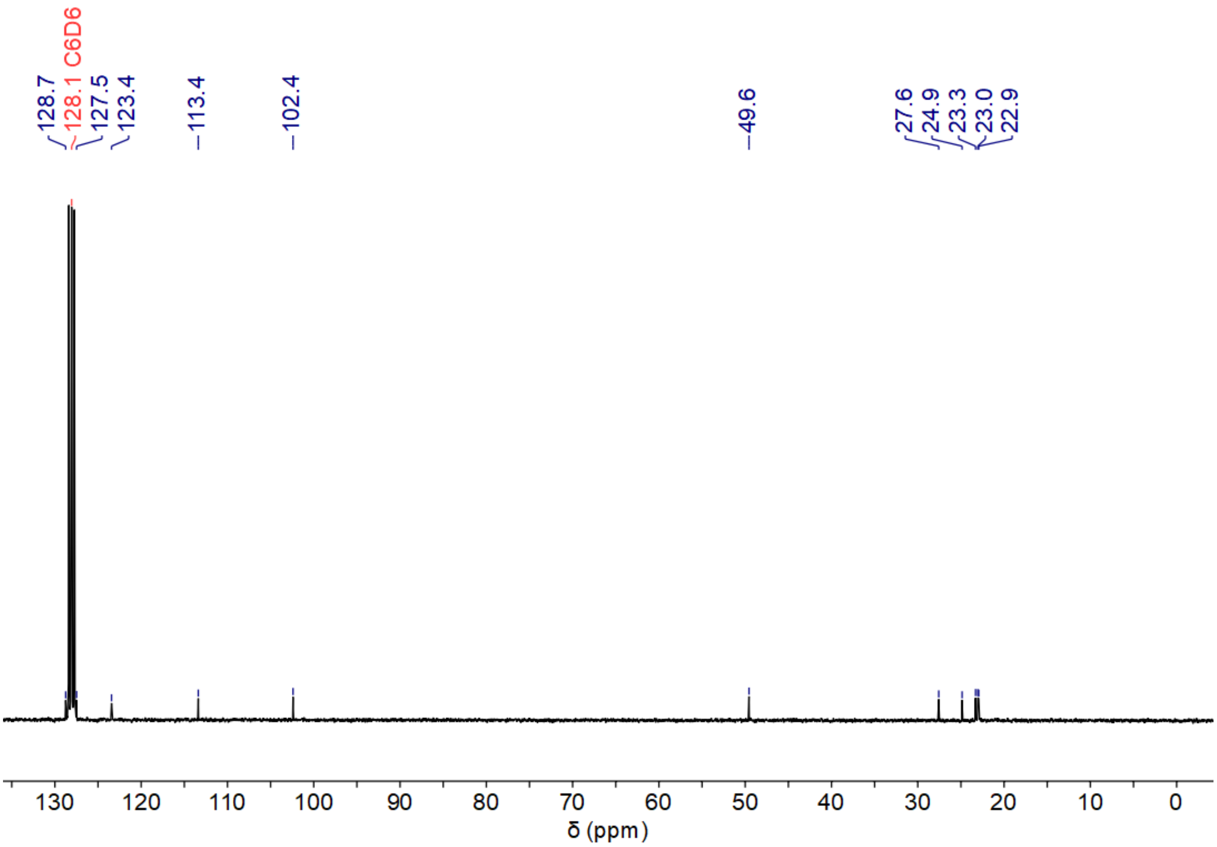

Figure S 13. ^{13}C NMR spectrum of ***meso*-4** (25 °C, benzene-d_6, 75.49 MHz).

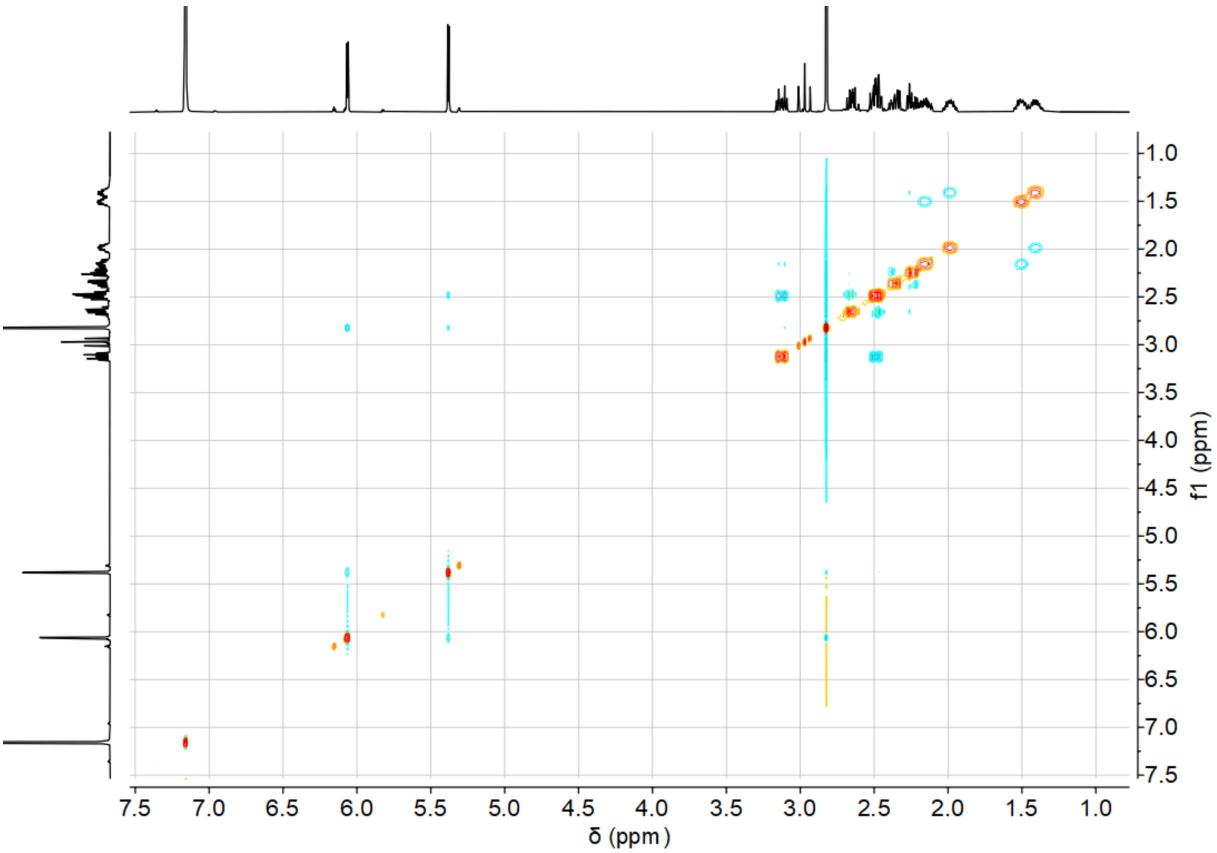

Figure S 14. ^1H-^1H NOESY NMR spectrum of ***meso*-4** (25 °C, benzene-d_6, 400.13 MHz).

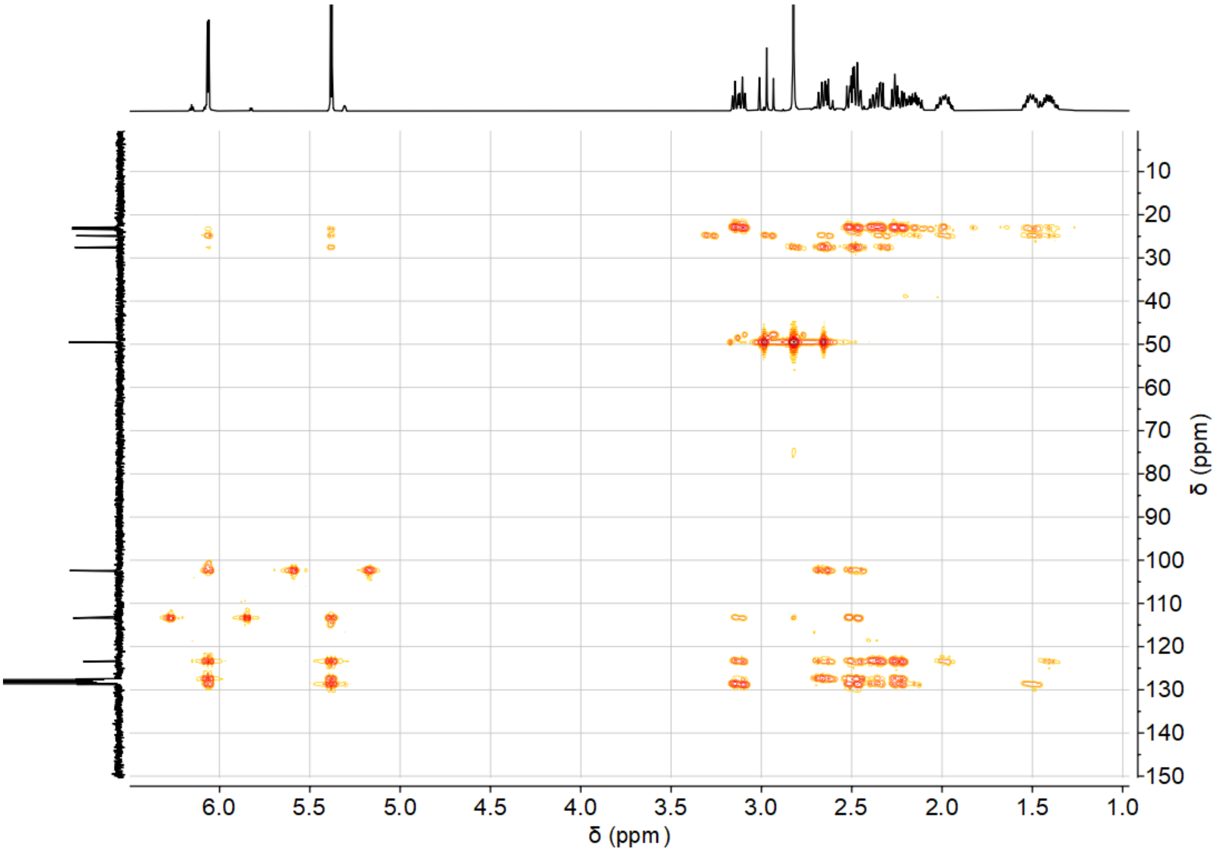

Figure S 15. ^1H-^{13}C HMBC NMR spectrum of ***meso*-4** (25 °C, benzene-d_6, 400.13 MHz, 100.62 MHz).

2.6. NMR spectra of catalytic dehydropolymerisation of $PhSiH_3$ using 0.1 mol% 2

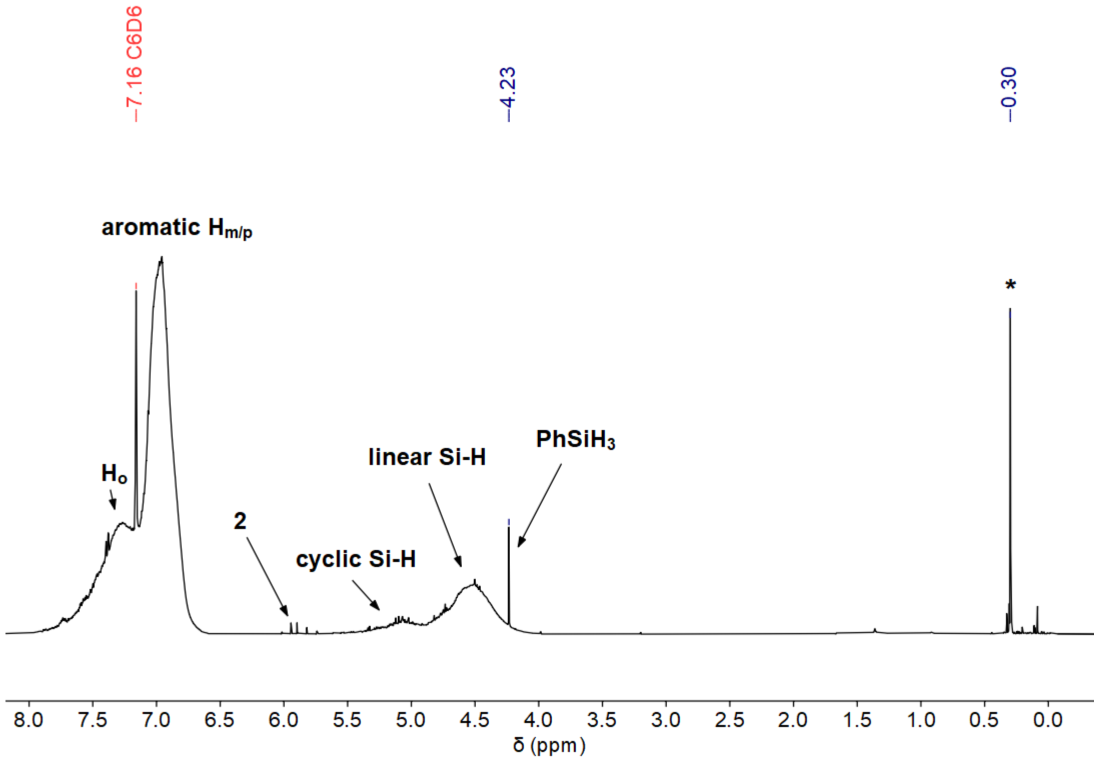

Figure S 16. ^{1}H NMR spectrum of catalytic dehydropolymerisation of $PhSiH_3$, asterisk marks silicon grease. Conditions: room temperature, open system with pressure compensation, 6.47 mmol $PhSiH_3$, 0.1 mol% **2** (25 °C, benzene-d_6, 400.13 MHz).

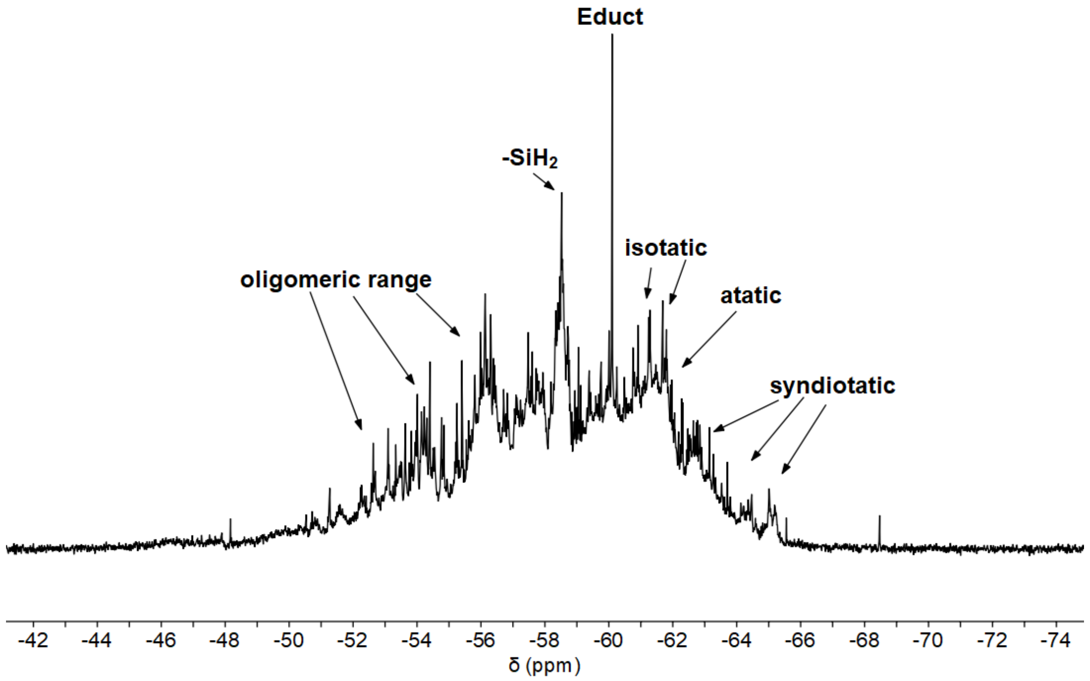

Figure S 17. ^{29}Si DEPT45 NMR spectrum of catalytic dehydropolymerisation of $PhSiH_3$. Conditions: room temperature, open system with pressure compensation, 6.47 mmol $PhSiH_3$, 0.1mol% **2** (25 °C, benzene-d_6, 79.49 MHz).

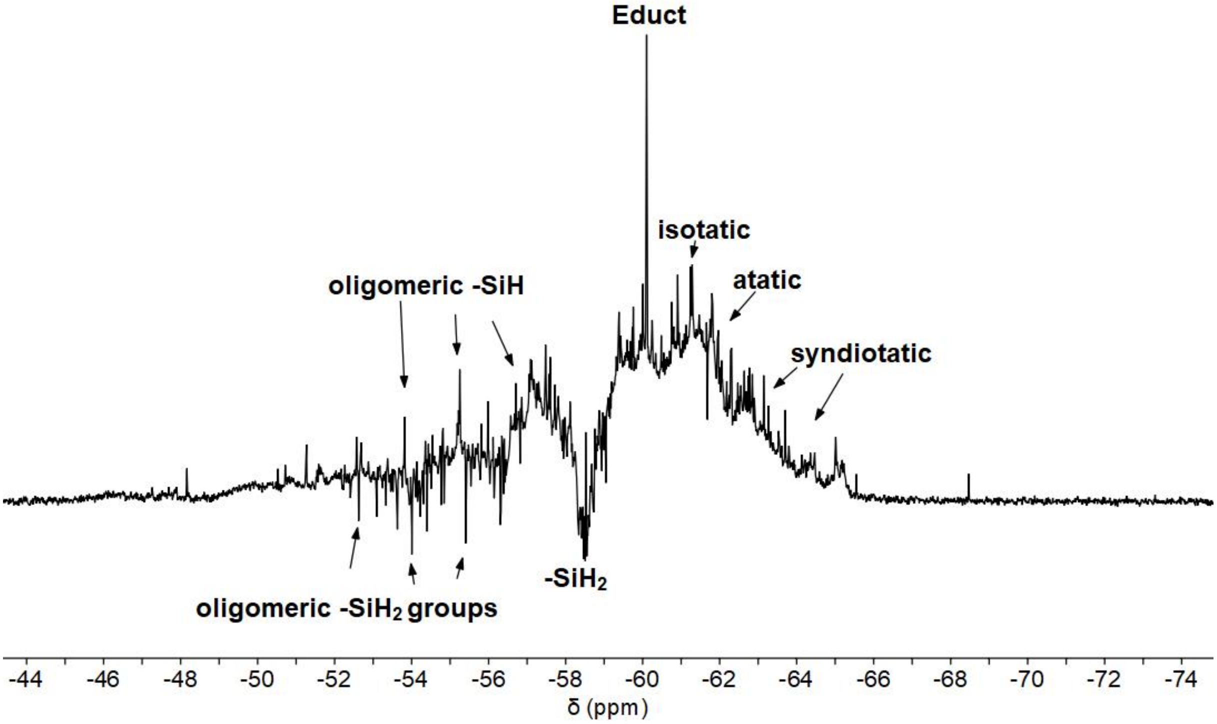

Figure S 18. ^{29}Si DEPT135 NMR spectrum of catalytic dehydropolymerisation of $PhSiH_3$. Conditions: room temperature, open system with pressure compensation, 6.47 mmol $PhSiH_3$, 0.1 mol% **2** (25 °C, benzene-d_6, 79.49 MHz).

2.7. NMR spectra of catalytic dehydropolymerisation of $PhSiH_3$ using 0.2 mol% of 2

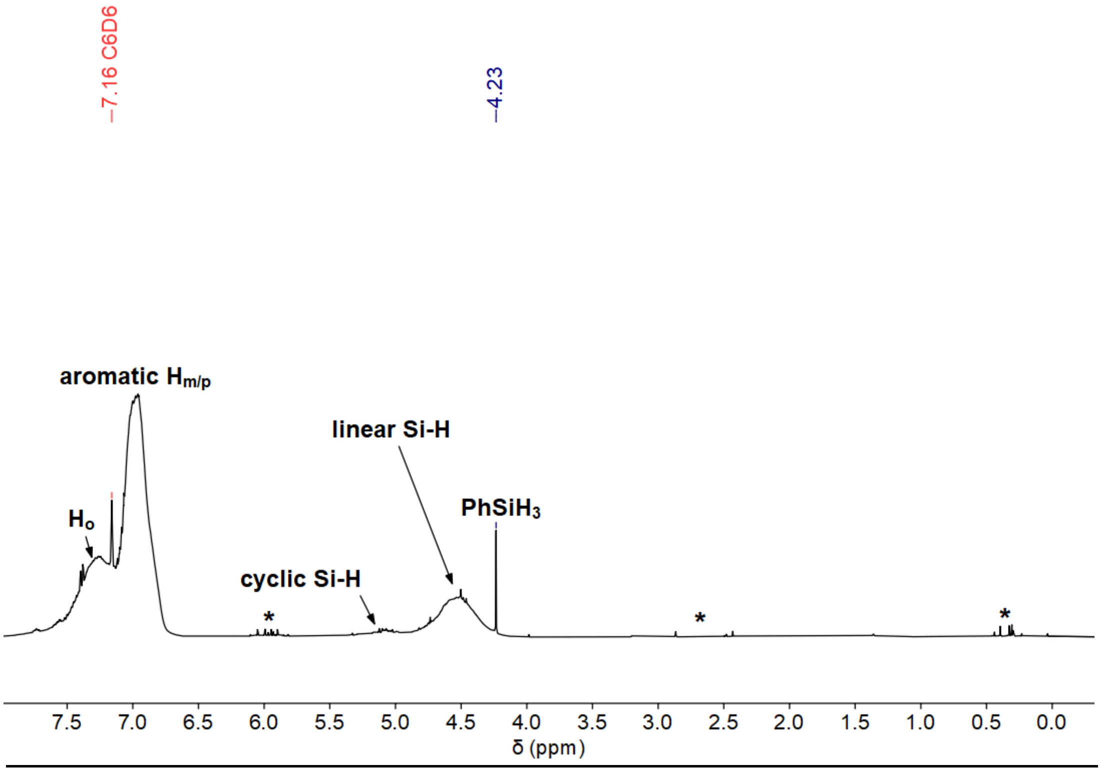

Figure S 19. ^{1}H NMR spectrum of catalytic dehydropolymerisation of $PhSiH_3$, asterisks mark decomposition products. Conditions: room temperature, open system with pressure compensation, 6.47 mmol $PhSiH_3$, 0.2 mol% **2** (25 °C, benzene-d_6, 400.13 MHz).

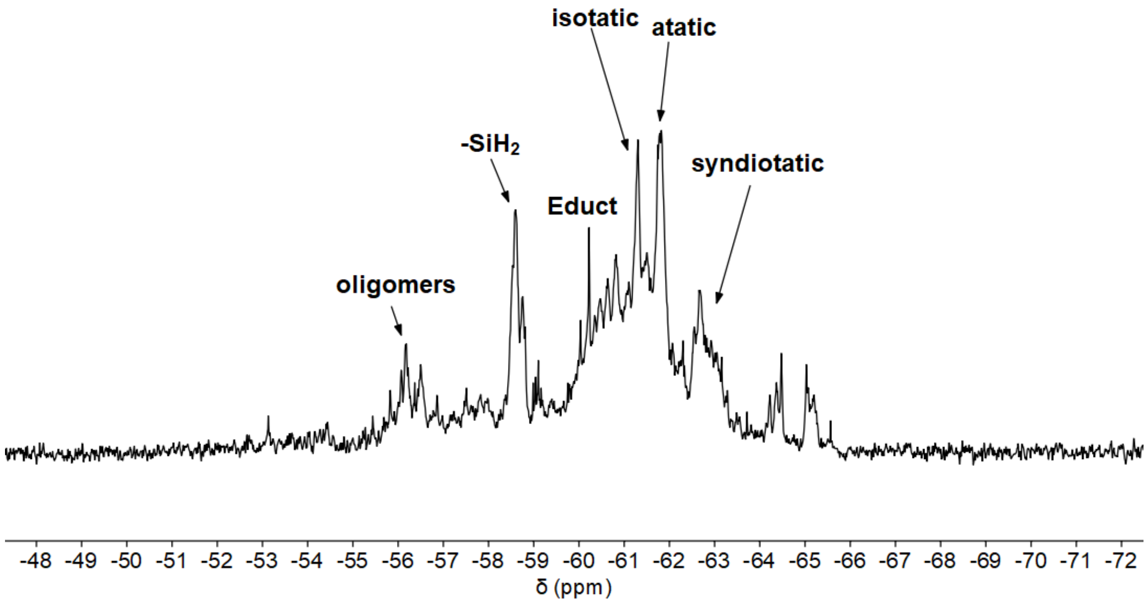

Figure S 20. ^{29}Si DEPT45 NMR spectrum of catalytic dehydropolymerisation of $PhSiH_3$. Conditions: room temperature, open system with pressure compensation, 6.47 mmol $PhSiH_3$, 0.2 mol% **2** (25 °C, benzene-d_6, 79.49 MHz).

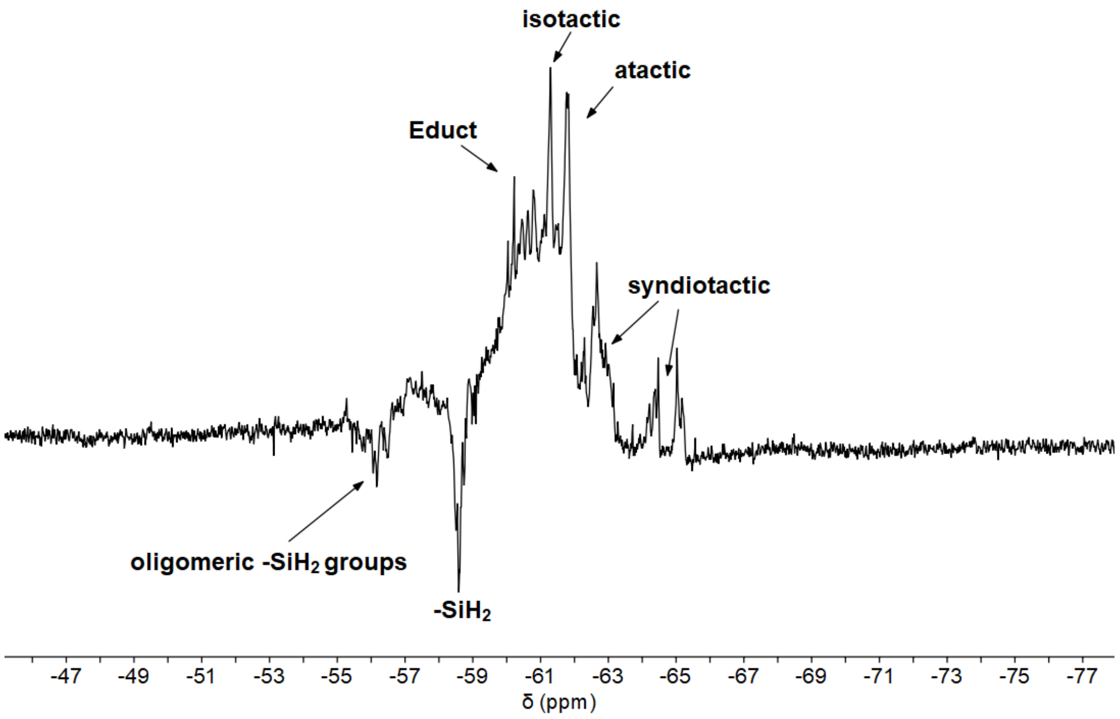

Figure S 21. ^{29}Si DEPT135 NMR spectrum of catalytic dehydropolymerisation of $PhSiH_3$. Conditions: room temperature, open system with pressure compensation, 6.47 mmol $PhSiH_3$, 0.2 mol% **2** (25 °C, benzene-d_6, 79.49 MHz).

2.8. NMR spectra of catalytic dehydropolymerisation of $PhSiH_3$ using 0.4 mol% 2

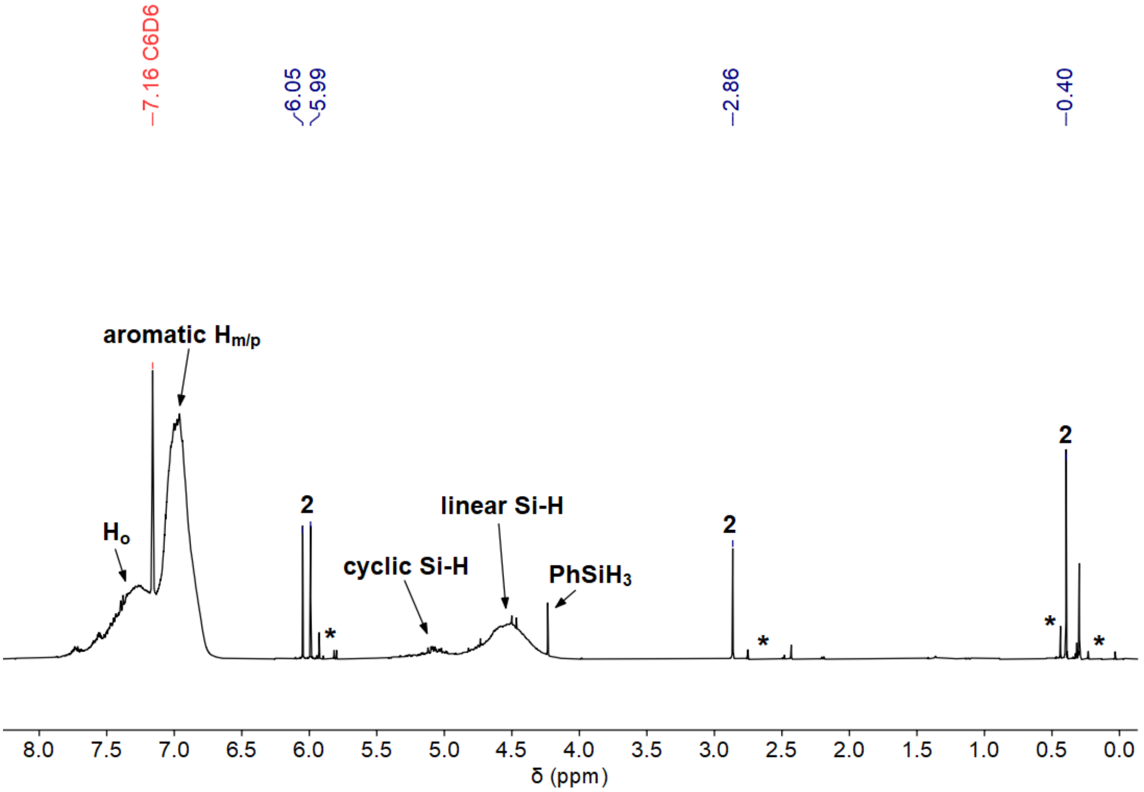

Figure S 22. ^{1}H NMR spectrum of catalytic dehydropolymerisation of $PhSiH_3$, asterisks mark decomposition products. Conditions: room temperature, open system with pressure compensation, 6.47 mmol $PhSiH_3$, 0.4 mol% **2** (25 °C, benzene-d_6, 400.13 MHz).

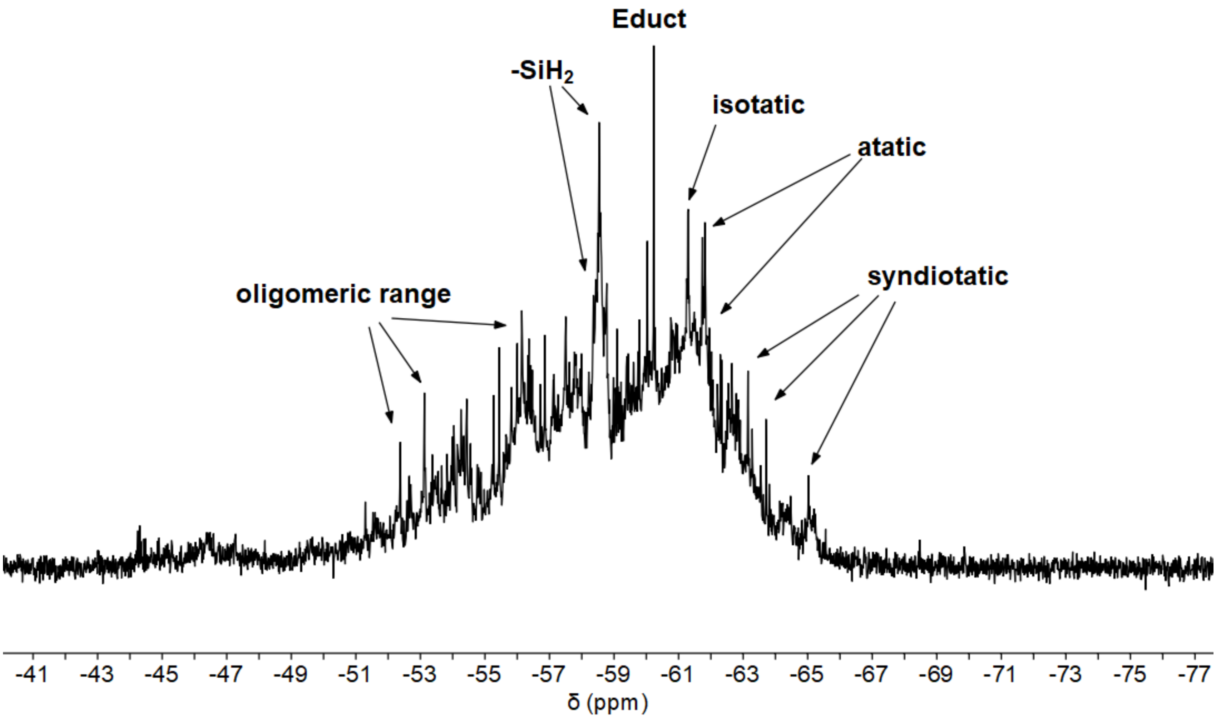

Figure S 23. ^{29}Si DEPT45 NMR spectrum of catalytic dehydropolymerisation of $PhSiH_3$. Conditions: room temperature, open system with pressure compensation, 6.47 mmol $PhSiH_3$, 0.4 mol% **2** (25 °C, benzene-d_6, 79.49 MHz).

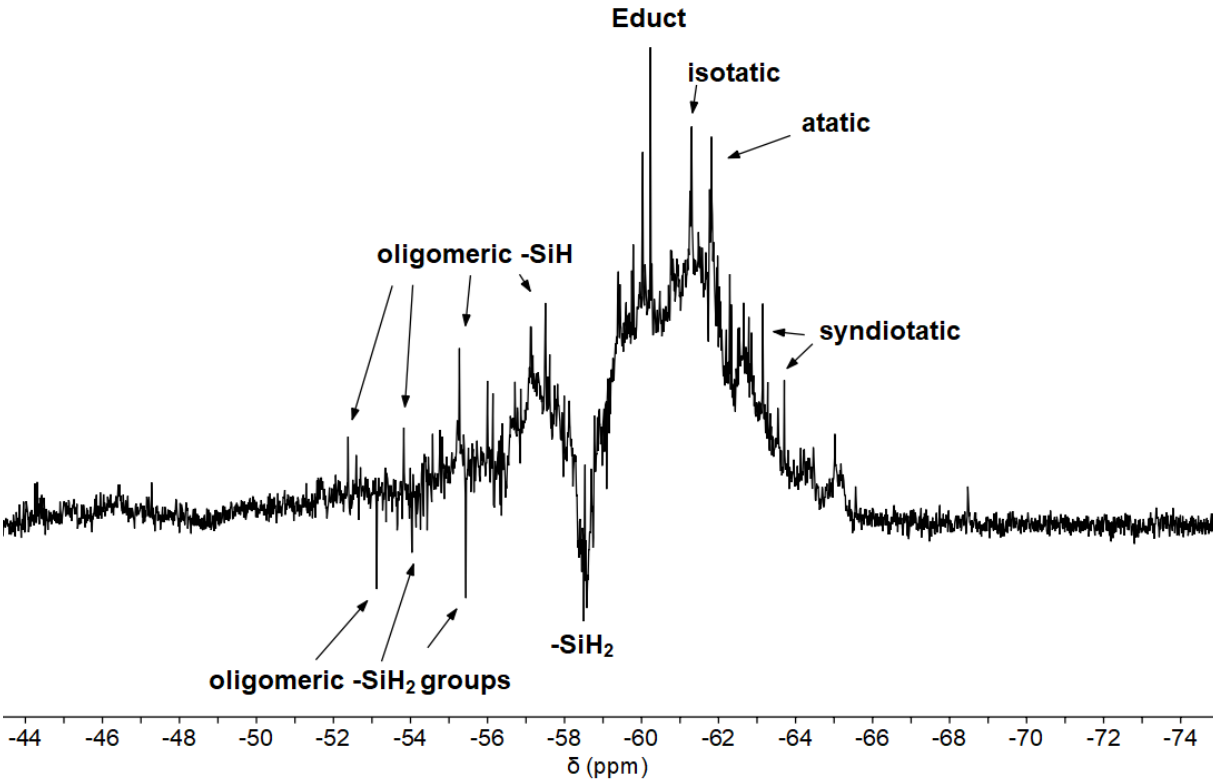

Figure S 24. ^{29}Si DEPT135 NMR spectrum of catalytic dehydropolymerisation of $PhSiH_3$. Conditions: room temperature, open system with pressure compensation, 6.47 mmol $PhSiH_3$, 0.4 mol% **2** (25 °C, benzene-d_6, 79.49 MHz).

2.9. NMR spectra of dehydropolymerisation experiment using 0.4 mol% of 2 with addition of a second portion of $PhSiH_3$

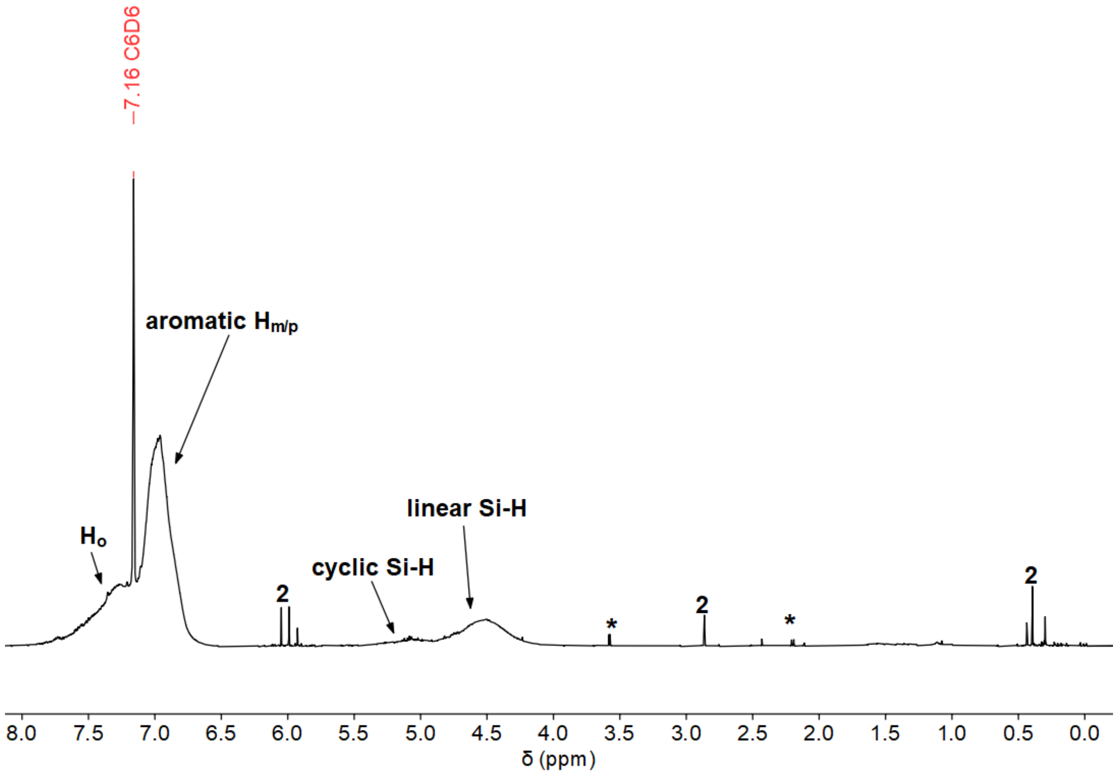

Figure S 25. ^{1}H NMR spectrum of catalytic dehydropolymerisation of $PhSiH_3$, asterisks mark decomposition products. Conditions: room temperature, open system with pressure compensation, 1.85 mmol $PhSiH_3$, 0.4 mol% **2** (25 °C, benzene-d_6, 400.13 MHz).

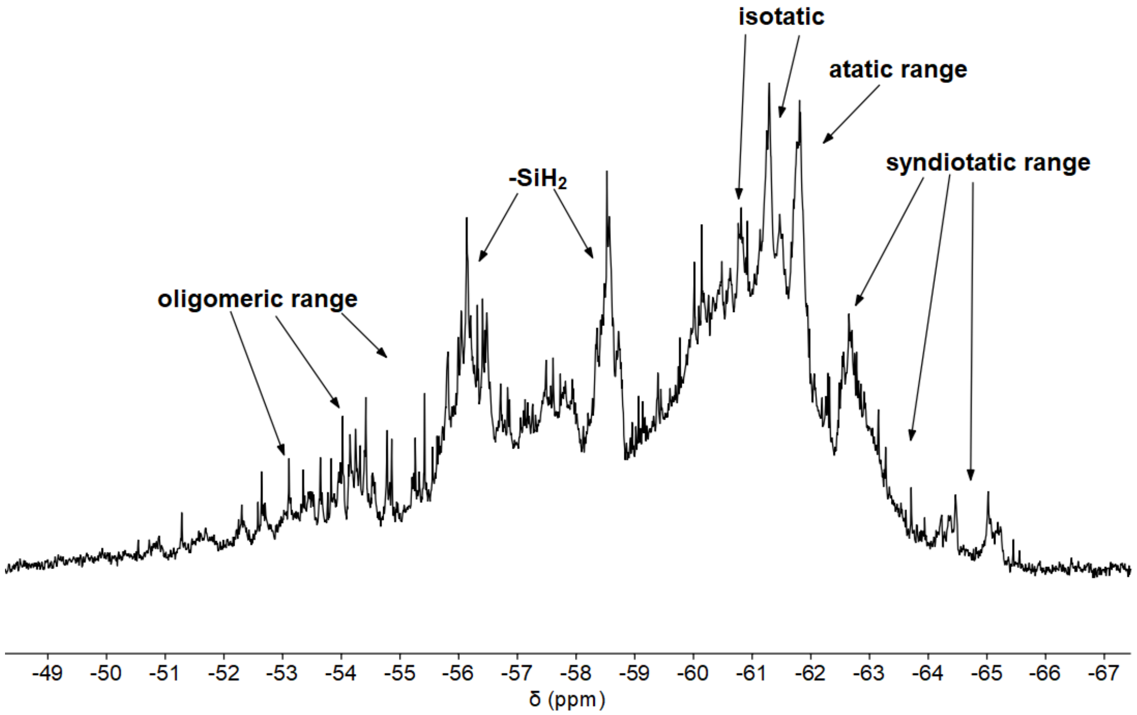

Figure S 26. ^{29}Si DEPT45 NMR spectrum of catalytic dehydropolymerisation of $PhSiH_3$. Conditions: room temperature, open system with pressure compensation, 1.85 mmol $PhSiH_3$, 0.4 mol% **2** (25 °C, benzene-d_6, 79.49 MHz).

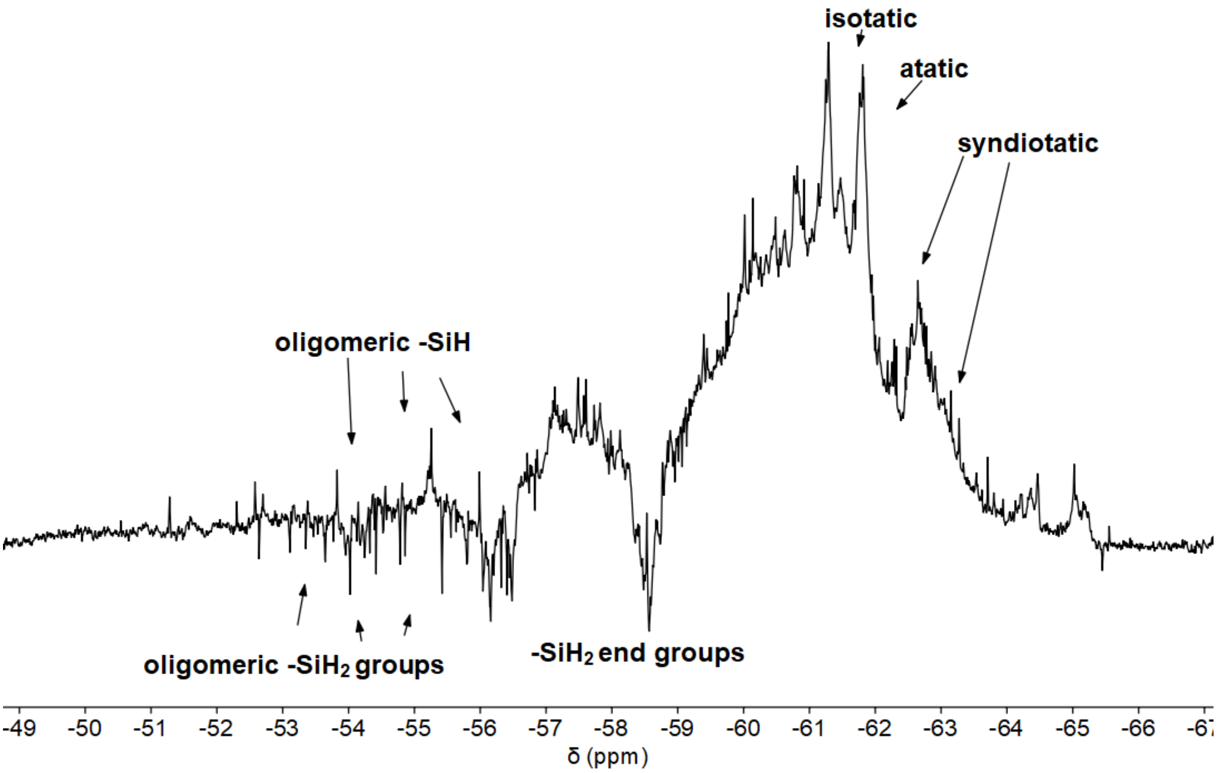

Figure S 27. ^{29}Si DEPT135 NMR spectrum of catalytic dehydropolymerisation of $PhSiH_3$. Conditions: room temperature, open system with pressure compensation, 0.4 mol% **2** (25 °C, benzene-d_6, 79.49 MHz).

2.10. NMR spectra of catalytic dehydropolymerisation of $PhSiH_3$ using 0.4 mol% of 1

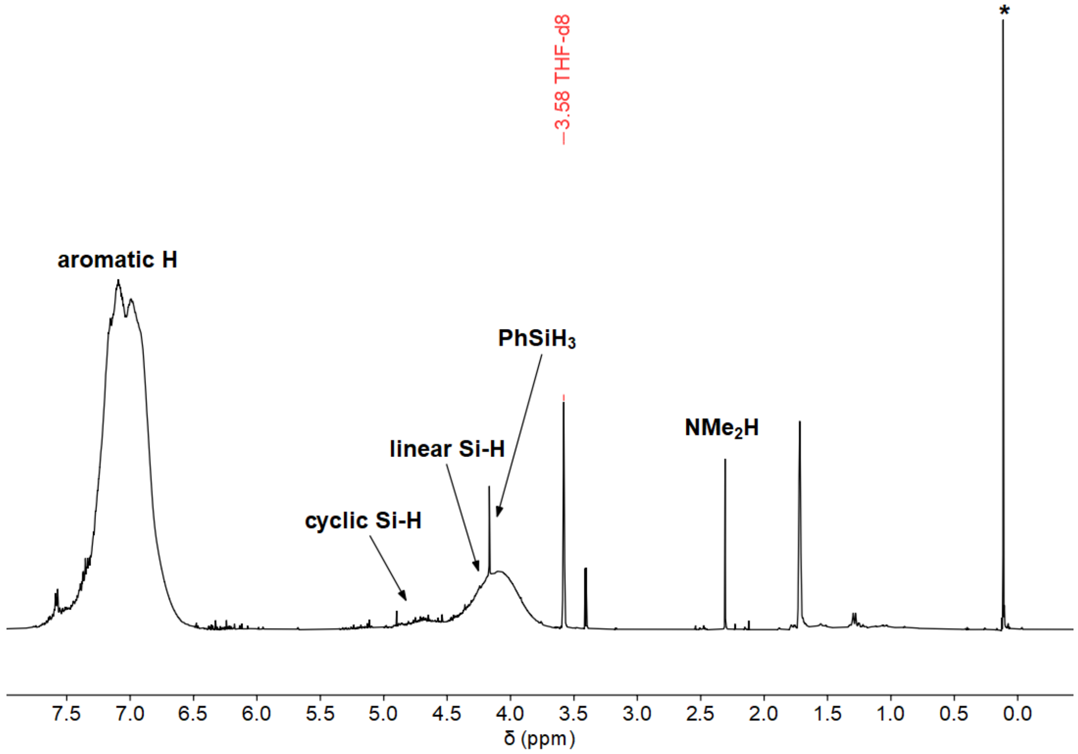

Figure S 28. ^{1}H NMR spectrum of catalytic dehydropolymerisation of $PhSiH_3$, asterisk marks silicon grease. Conditions: room temperature, open system with pressure compensation, 1.85 mmol $PhSiH_3$, 0.4 mol% **1** (25 °C, THF-d_8, 400.13 MHz).

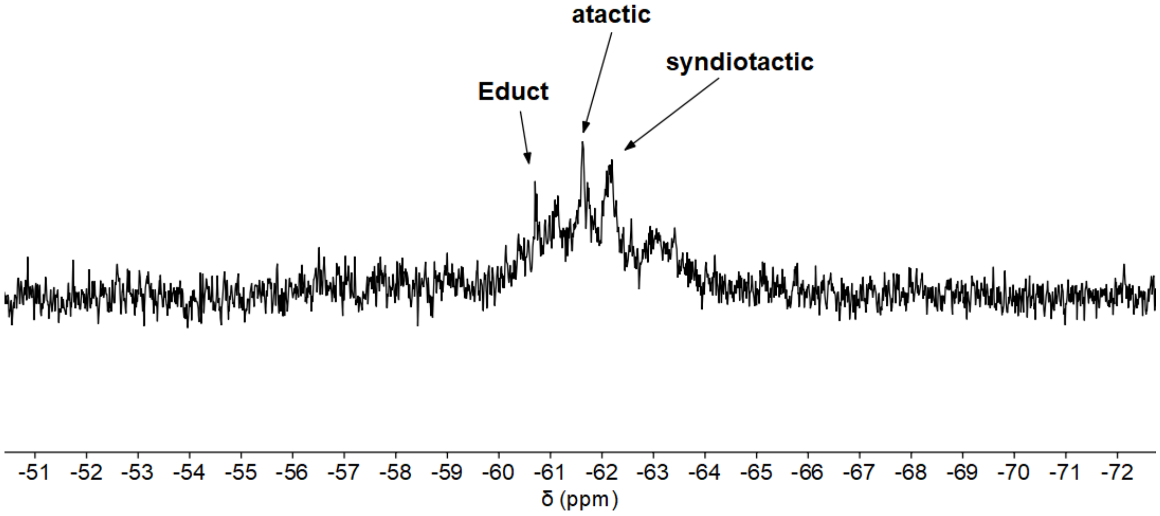

Figure S 29. ^{29}Si NMR spectrum of catalytic dehydropolymerisation of $PhSiH_3$. Conditions: room temperature, open system with pressure compensation, 1.85 mmol $PhSiH_3$, 0.4 mol% **1** (25 °C, THF-d_8, 79.49 MHz).

2.11. NMR spectra of catalytic dehydropolymerisation of $PhSiH_3$ using 0.8 mol% of 1

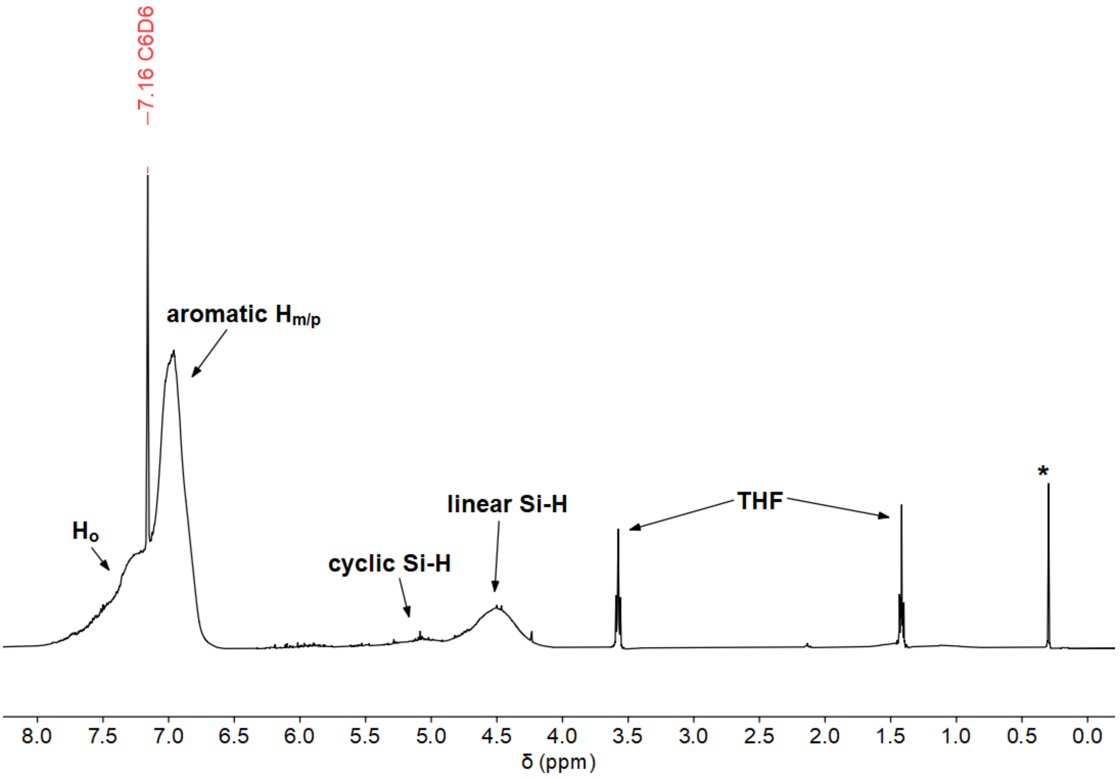

Figure S 30. ^{1}H NMR spectrum of catalytic dehydropolymerisation of $PhSiH_3$, asterisk marks silicon grease. Conditions: room temperature, open system with pressure compensation, 1.85 mmol $PhSiH_3$, 0.8 mol% **1** (25 °C, Benzene-d_6, 400.13 MHz).

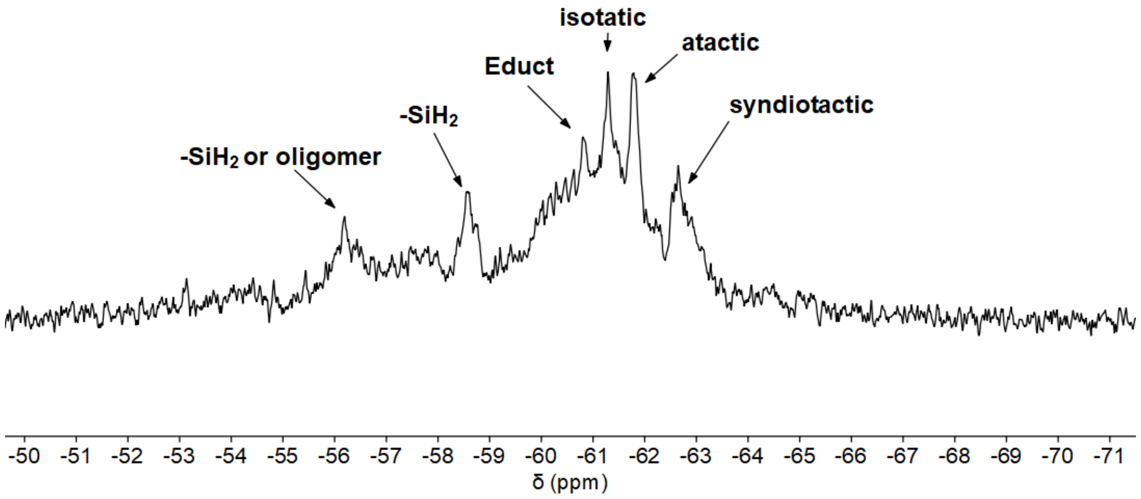

Figure S 31. ^{29}Si DEPT45 NMR spectrum of catalytic dehydropolymerisation of $PhSiH_3$. Conditions: room temperature, open system with pressure compensation, 1.85 mmol $PhSiH_3$, 0.8 mol% **1** (25 °C, Benzene-d_6, 79.49 MHz).

2.12. NMR spectrum of catalytic dehydropolymerisation of *p*-TolSiH$_3$ using 0.2 mol% of 2

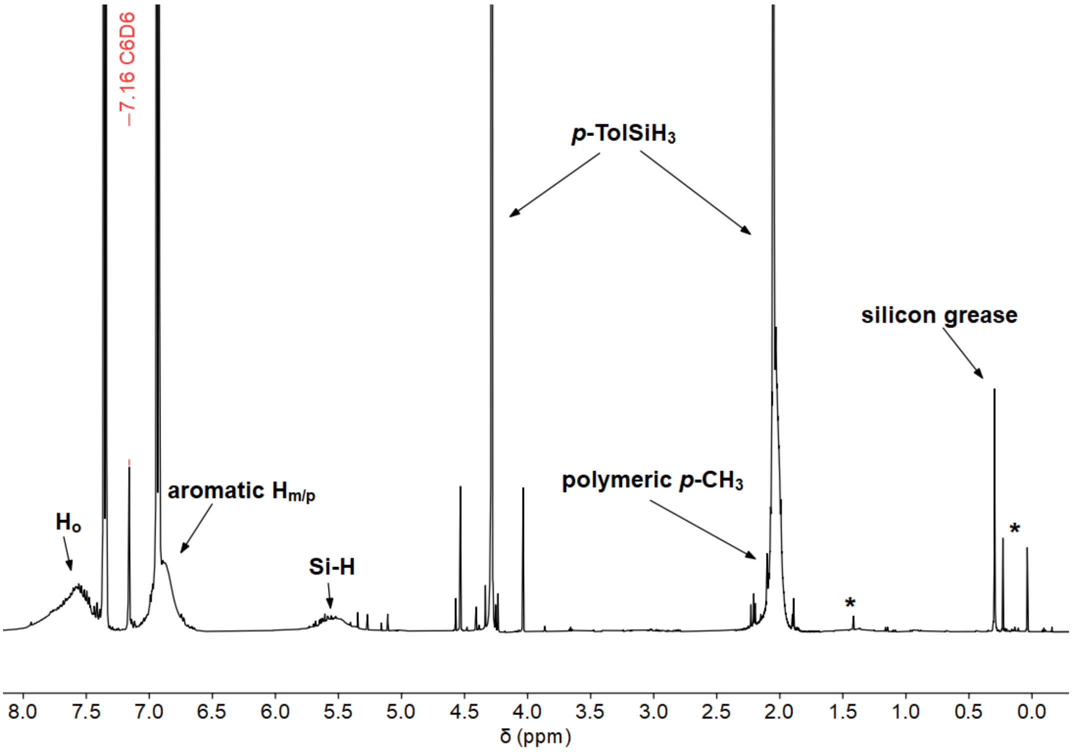

Figure S 32. ^{1}H NMR spectrum of catalytic dehydropolymerisation of *p*-tolSiH$_3$, asterisks mark formation of [1,3-bis-(trimethylsilyl)prop-1-yne]. Conditions: room temperature, open system with pressure compensation, 1.85 mmol PhSiH$_3$, 0.2 mol% **2** (25 °C, benzene-*d*$_6$, 400.13 MHz).

2.13. NMR spectra of catalytic dehydropolymerisation of PhSiH$_3$ using 0.2 mol% of 2 in a closed system

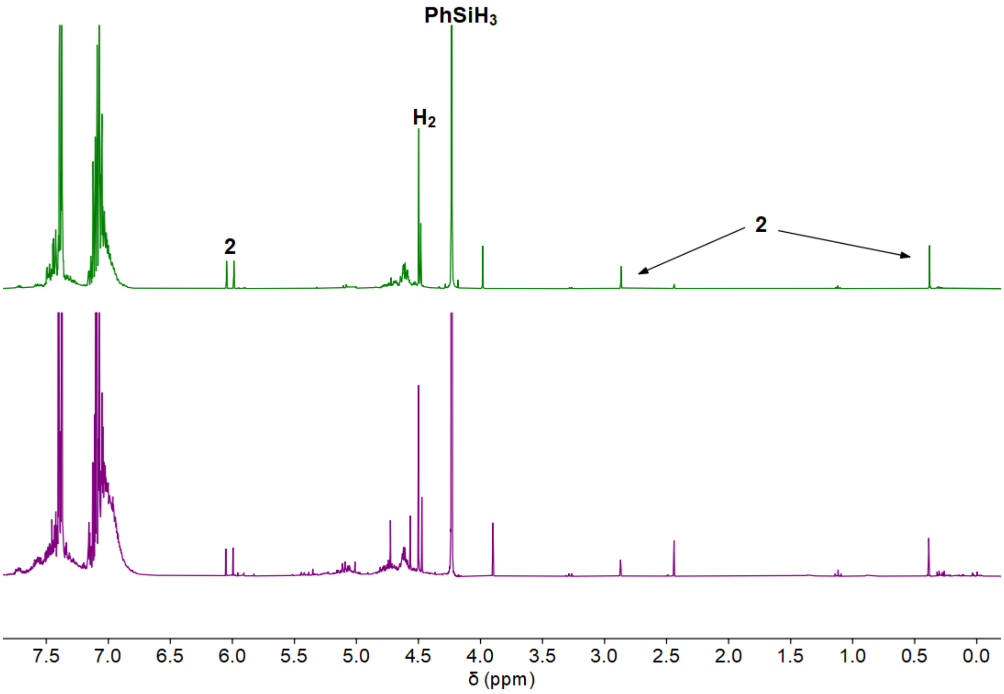

Figure S 33. ^{1}H NMR spectra of catalytic dehydropolymerisation of PhSiH$_3$. Conditions: room temperature, 1.85 mmol PhSiH$_3$, 0.2 mol% **2** (green: closed system stored overnight, purple: open system stored in a glove box for 2 days) (25 °C, benzene-*d*$_6$, 400.13 MHz).

2.14. NMR spectra of catalytic dehydropolymerisation of $PhSiH_3$ using 0.2 mol% of 2 in open system with solvent

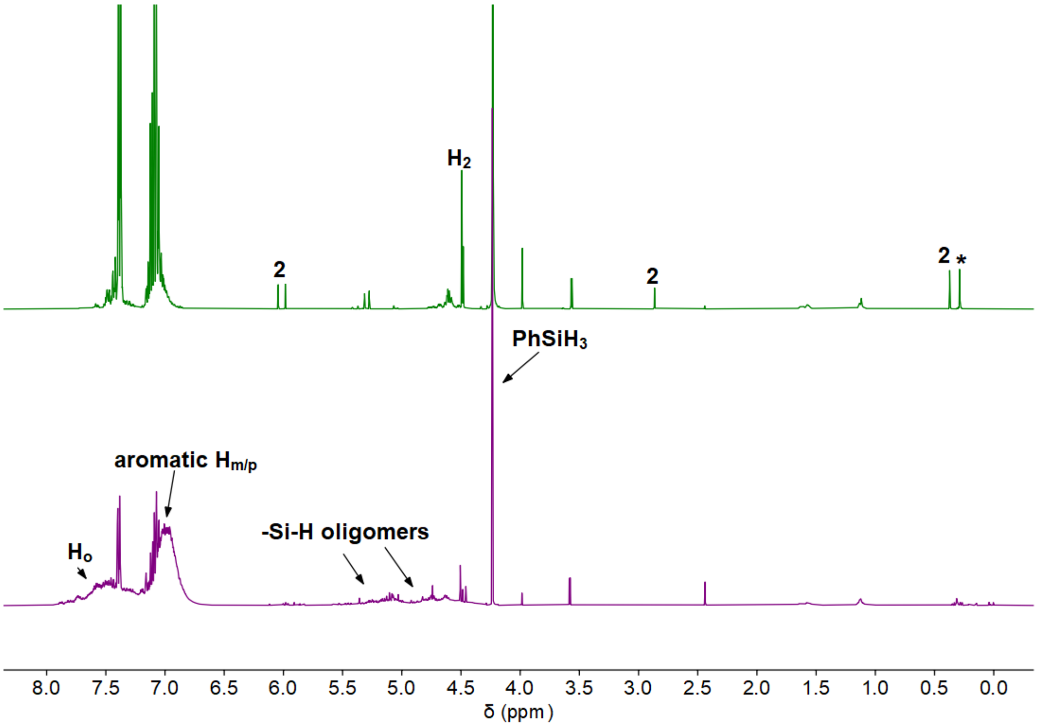

Figure S 34. ^{1}H NMR spectra of catalytic dehydropolymerisation of $PhSiH_3$.Conditions: room temperature, 1.85 mmol $PhSiH_3$, 0.2 mol% **2** (green: open NMR tube stored overnight, purple: open system stored in a glove box for two weeks (25 °C, benzene-d_6, 400.13 MHz).

2.15. NMR spectra of catalytic dehydropolymerisation of $PhSiH_3$ using 0.4 mol% of *rac*-3

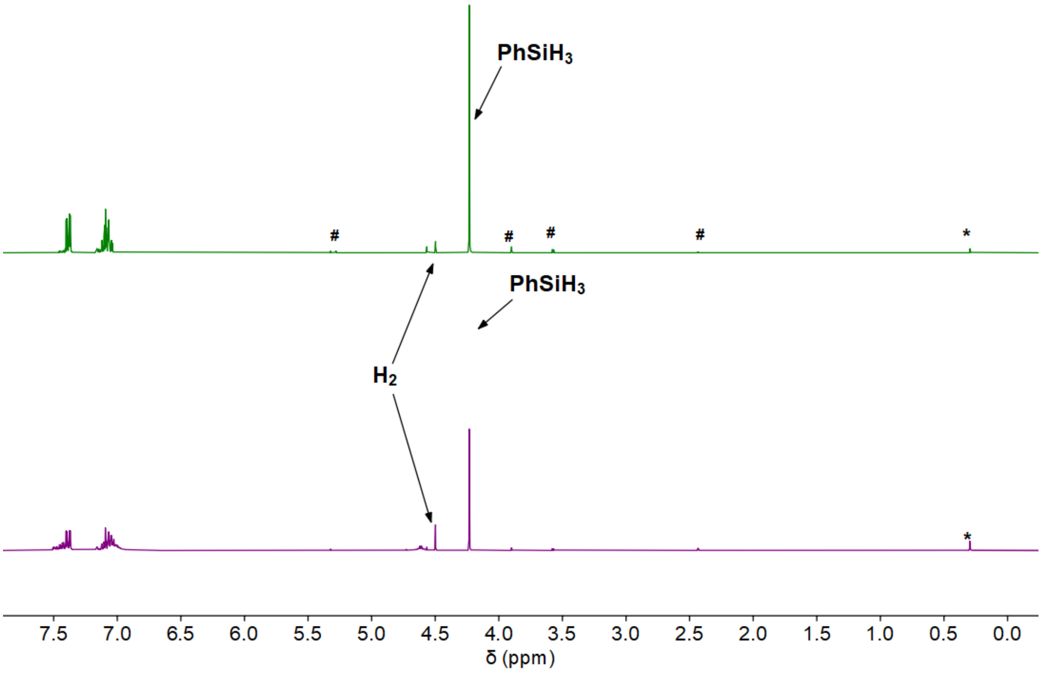

Figure S 35. ^{1}H NMR spectra of catalytic dehydropolymerisation of $PhSiH_3$. Asterisk marks silicon grease, hash marks unknown species. Conditions: 1.85 mmol $PhSiH_3$, 0.4 mol% ***rac*-3** (green: open system with pressure compensation, room temperature, purple: open system with pressure compensation, stirred 3 days at 60 °C) (25 °C, benzene-d_6, 300.20 MHz).

2.16. NMR spectra of catalytic dehydropolymerisation of $PhSiH_3$ using 0.8 mol% of *rac*-3

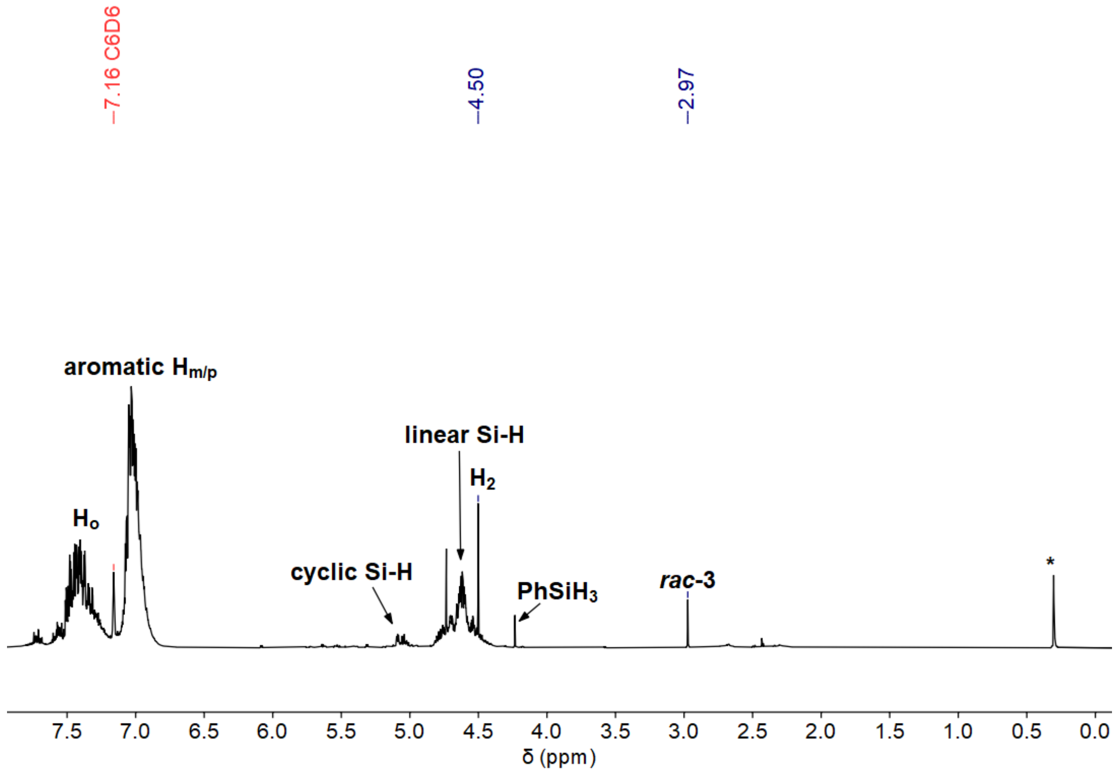

Figure S 36. ^{1}H NMR spectra of catalytic dehydropolymerisation of $PhSiH_3$. Asterisk marks silicon grease. Conditions: open system with pressure compensation, 1.85 mmol $PhSiH_3$, 0.8 mol% ***rac*-3**, 60 °C, 5 days (25 °C, benzene-*d*$_6$, 300.20 MHz).

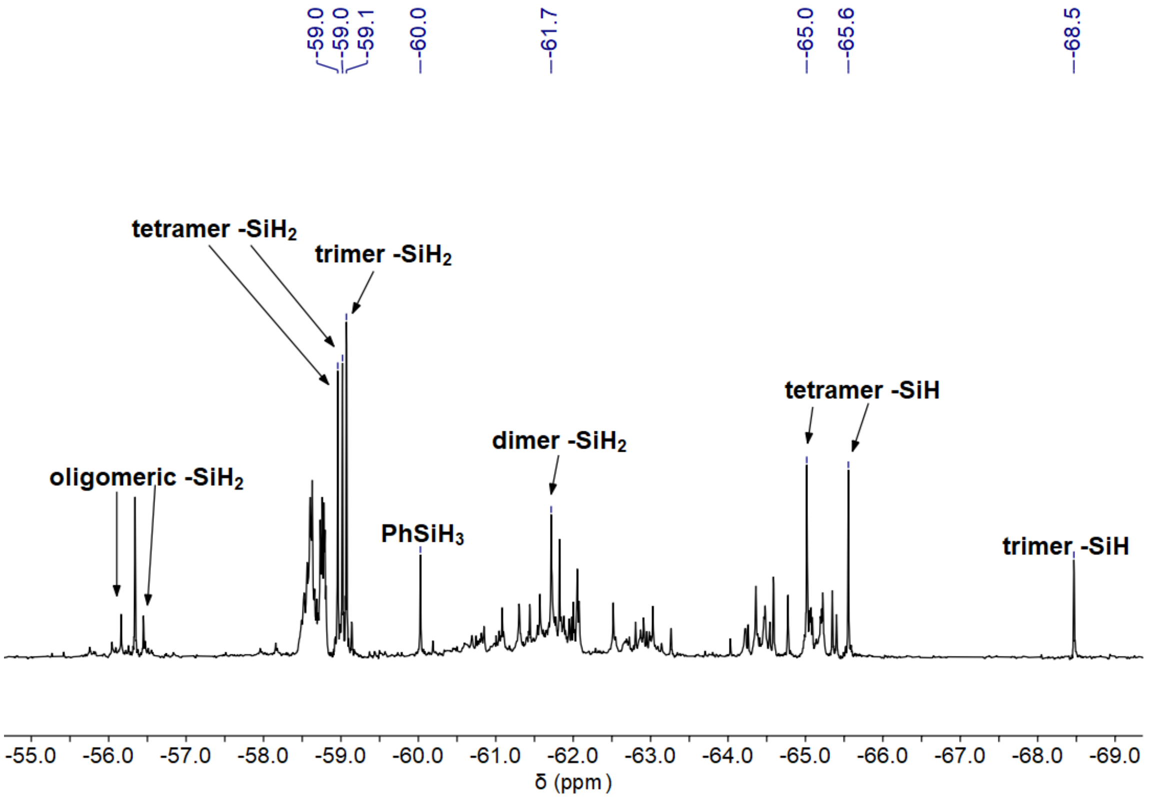

Figure S 37. ^{29}Si DEPT45 NMR spectrum of catalytic dehydropolymerisation of $PhSiH_3$. Conditions: open system with pressure compensation, 1.85 mmol $PhSiH_3$, 0.8 mol% ***rac*-3**, 60 °C, 5 days (25 °C, benzene-*d*$_6$, 79.49 MHz).

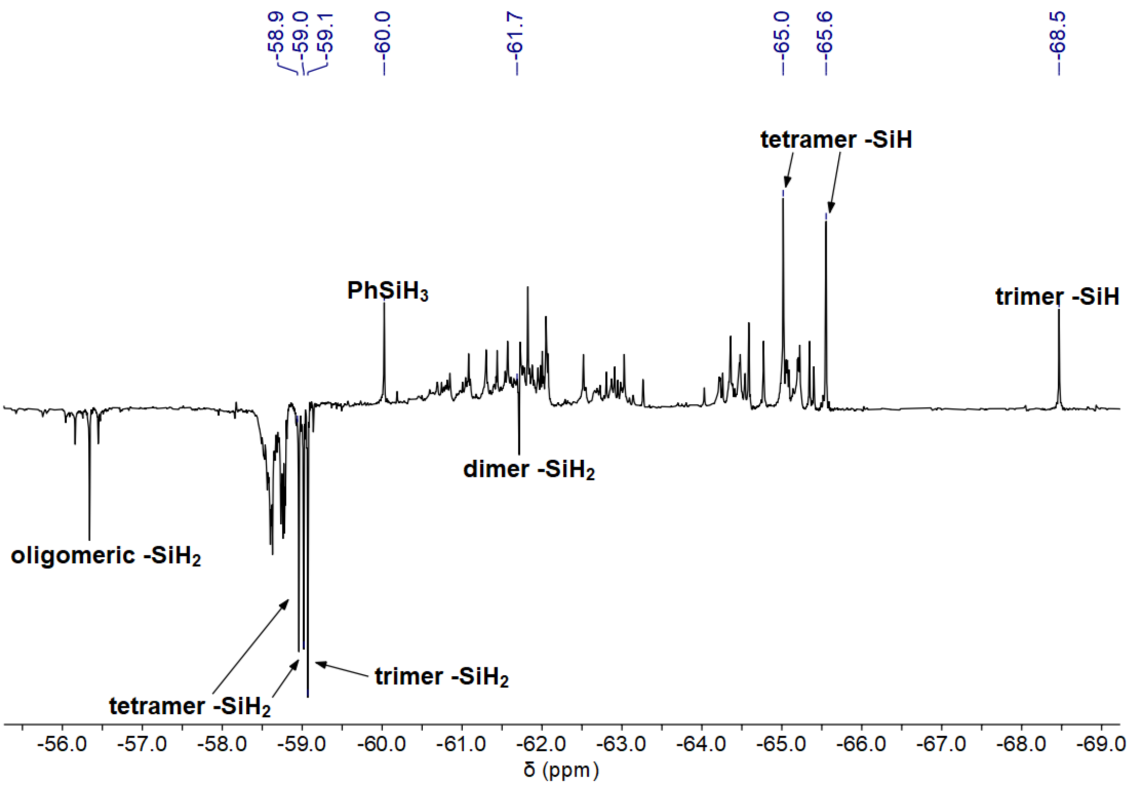

Figure S 38. ^{29}Si DEPT135 NMR spectrum of catalytic dehydropolymerisation of $PhSiH_3$. Conditions: open system with pressure compensation, 1.85 mmol $PhSiH_3$, 0.8 mol% ***rac*-3**, 60 °C, 5 days (25 °C, benzene-d_6, 79.49 MHz).

2.17. NMR spectrum of catalytic dehydropolymerisation of $PhSiH_3$ using 0.4 mol% of *meso*-4

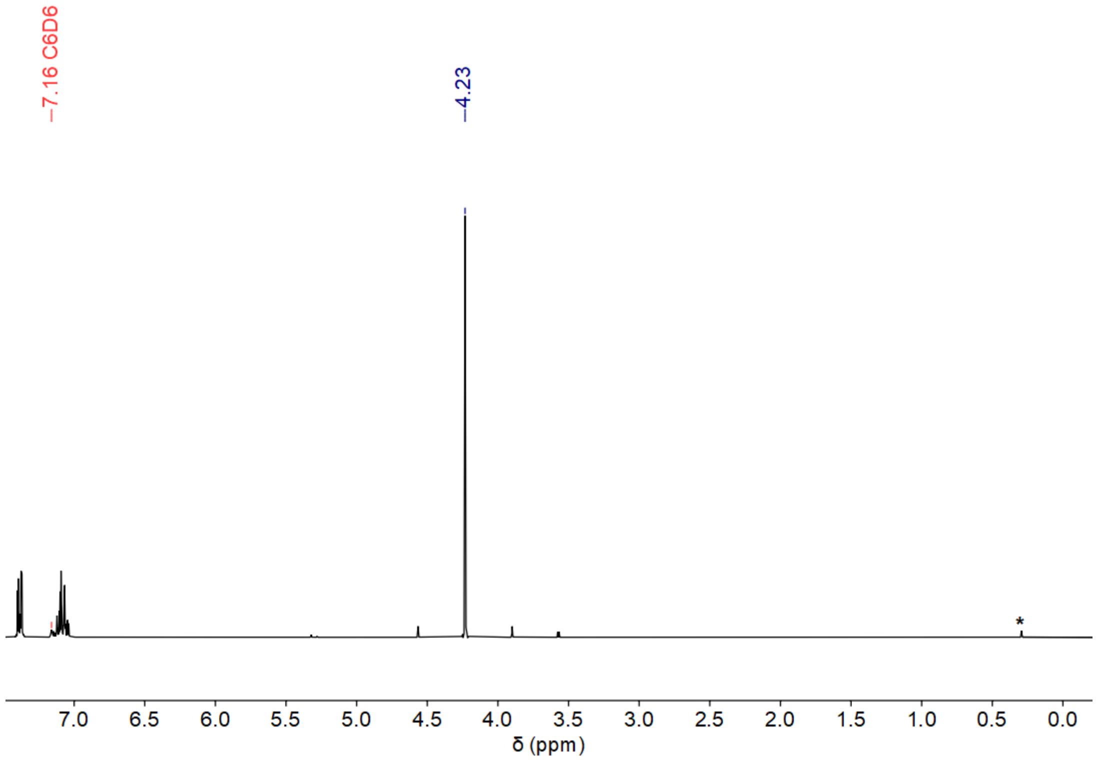

Figure S 39. ^{1}H NMR spectrum of catalytic dehydropolymerisation of $PhSiH_3$. Asterisk marks silicon grease. Conditions: room temperature, open system with pressure compensation, 1.85 mmol $PhSiH_3$, 0.8 mol% ***meso*-4** (25 °C, benzene-d_6, 300.20 MHz).

2.18. NMR spectrum of catalytic dehydropolymerisation of $PhSiH_3$ using 0.4 mol% of *rac*-4

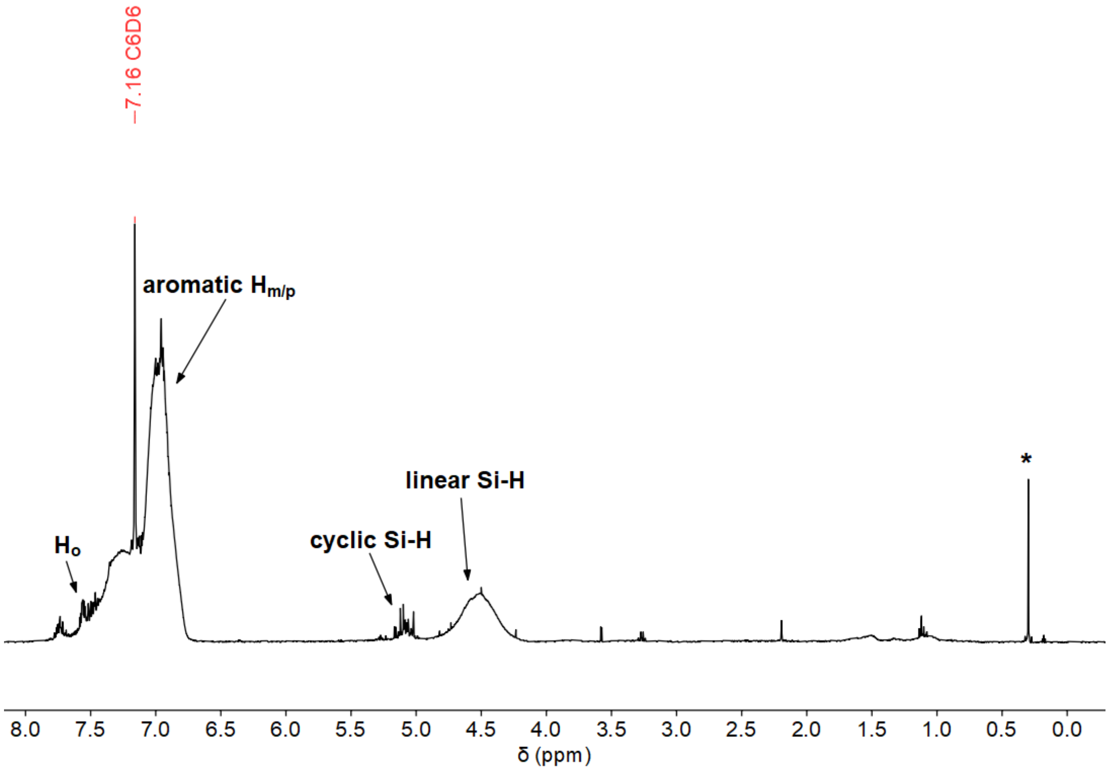

Figure S 40. ^{1}H NMR spectra of catalytic dehydropolymerisation of $PhSiH_3$. Asterisk marks silicon grease. Conditions: room temperature, open system with pressure compensation, 1.85 mmol $PhSiH_3$**,** 0.4 mol% ***rac*-4** (25 °C, benzene-d_6, 300.20 MHz).

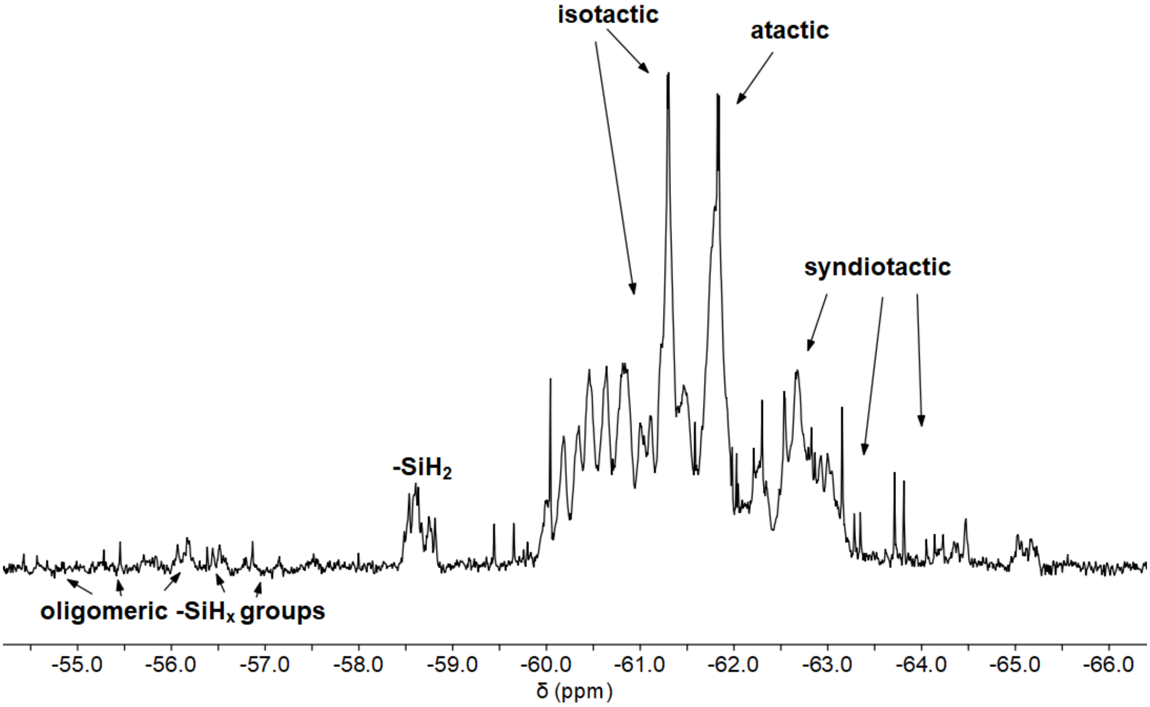

Figure S 41. ^{29}Si DEPT45 NMR spectrum of catalytic dehydropolymerisation of $PhSiH_3$. Conditions: room temperature, open system with pressure compensation, 1.85 mmol $PhSiH_3$**,** 0.4 mol% ***rac*-4** (25 °C, benzene-d_6, 79.49 MHz).

2.19. NMR spectrum of stoichiometric reaction of 2 with 4 eq $PhSiH_3$

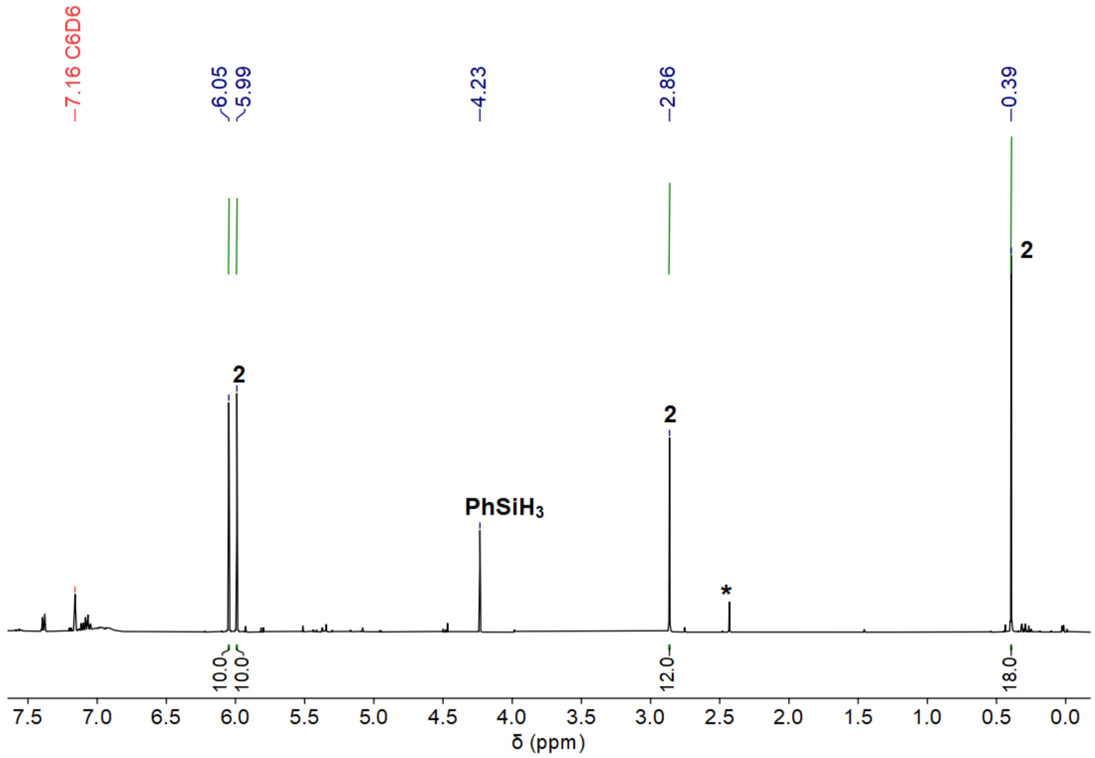

Figure S42. ^1H NMR spectrum of stoichiometric reaction, asterisk marks unknown species. Conditions: room temperature, 0.02mmol $PhSiH_3$, 0.006 mmol **2** (25 °C, benzene-d_6, 400.13 MHz).

2.20. NMR spectra of the catalytic reaction of $PhSiH_3$ with $LiNMe_2$

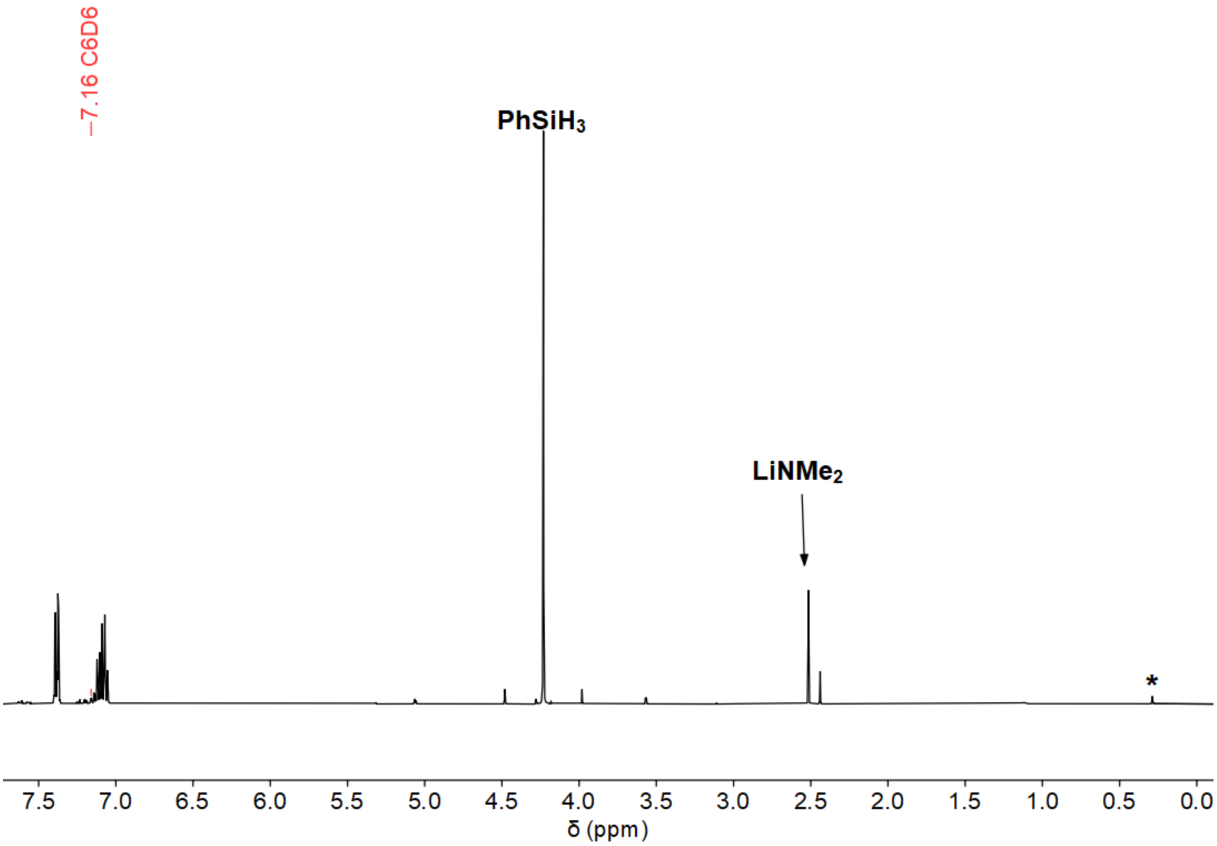

Figure S43. ^1H NMR spectrum of the catalytic reaction stirred overnight, asterisk marks silicon grease. Conditions: room temperature, 1.39 mmol $PhSiH_3$, 0.06 mmol $LiNMe_2$ (25 °C, benzene-d_6, 400.13 MHz).

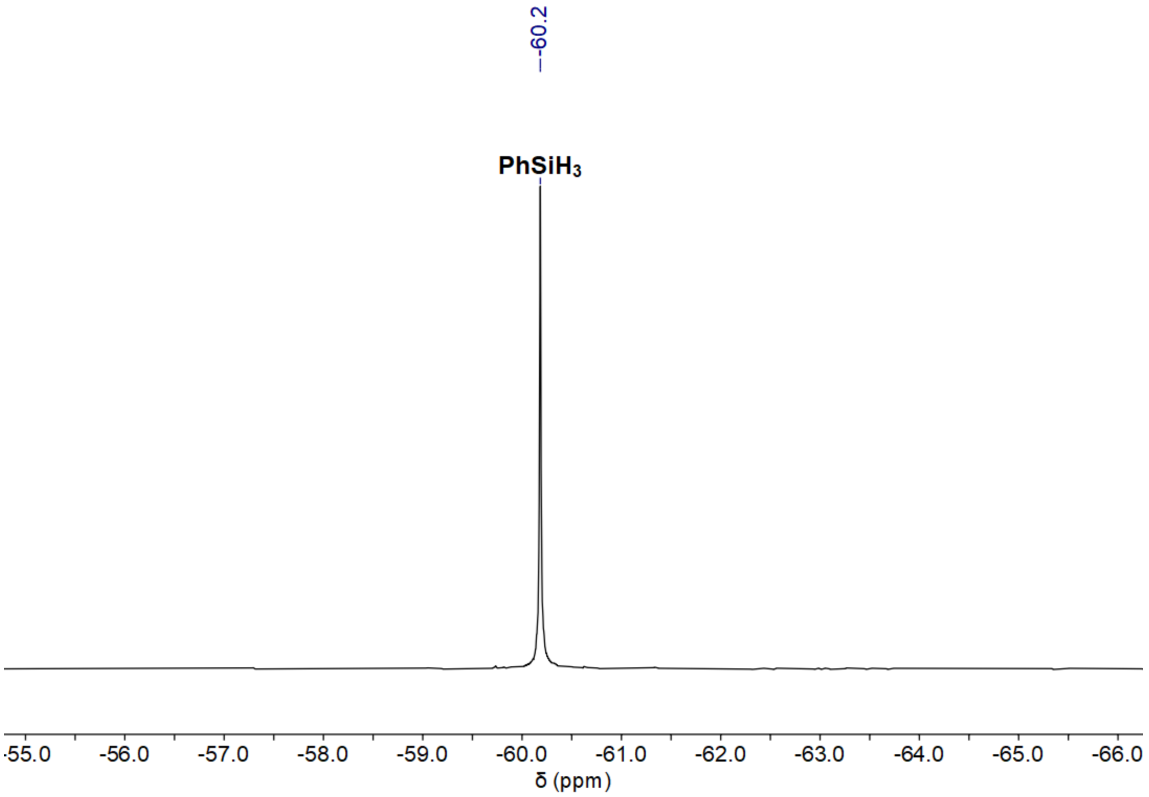

Figure S 44. ^{29}Si NMR spectrum of the catalytic reaction. Conditions: room temperature, 1.39 mmol $PhSiH_3$, 0.06 mmol $LiNMe_2$ (25 °C, benzene-d_6, 79.49 MHz).

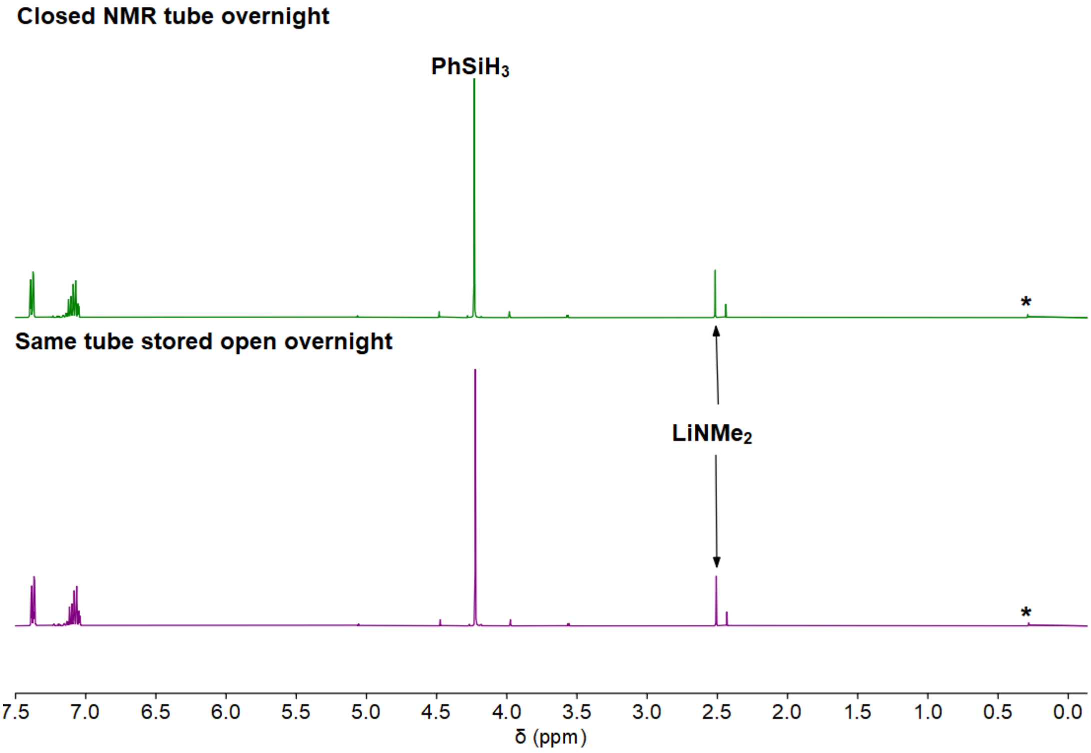

Figure S 45. ^{1}H NMR spectra of the catalytic reaction, asterisk marks silicon grease. Conditions: room temperature, 1.39 mmol $PhSiH_3$, 0.06 mmol $LiNMe_2$ (25 °C, benzene-d_6, 400.13 MHz).

2.21. NMR spectra of the reaction of $PhSiH_3$ with tetramethylethylenediamine (TMEDA)

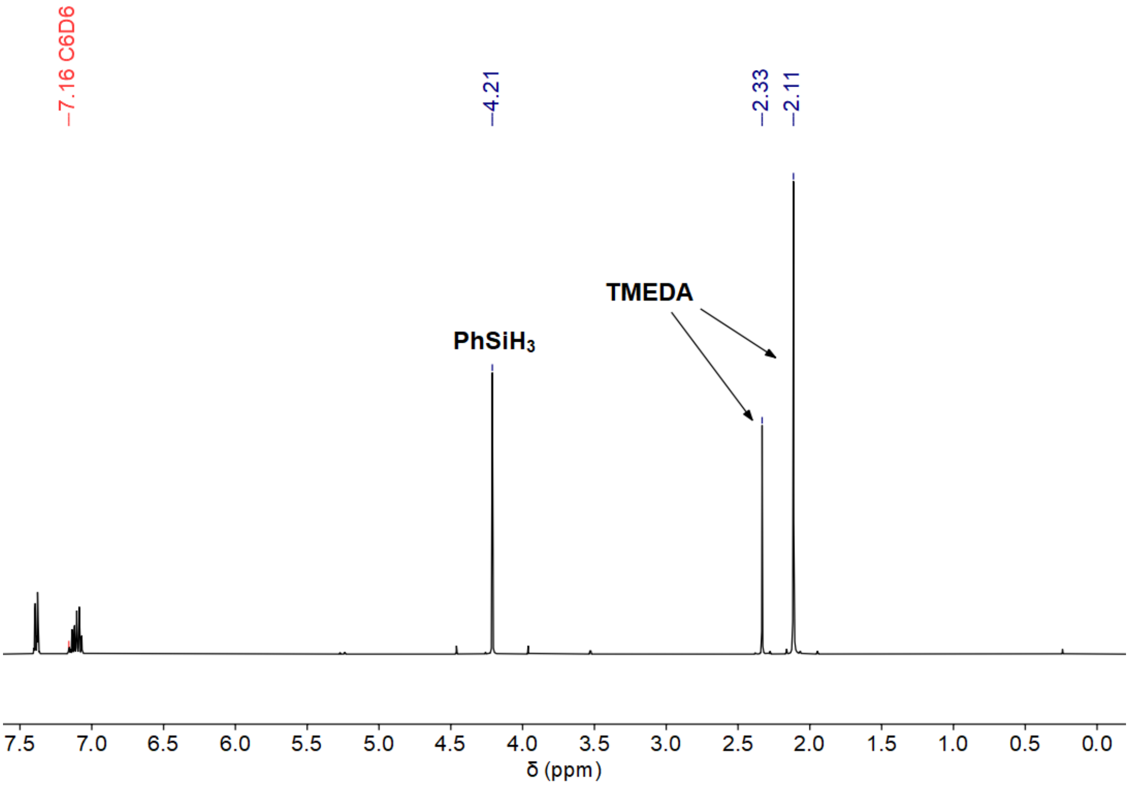

Figure S 46. ^1H NMR spectrum of the reaction. Conditions: room temperature, 1.85 mmol $PhSiH_3$, 0.37 mmol tetramethylethylenediamine (25 °C, benzene-d_6, 400.13 MHz).

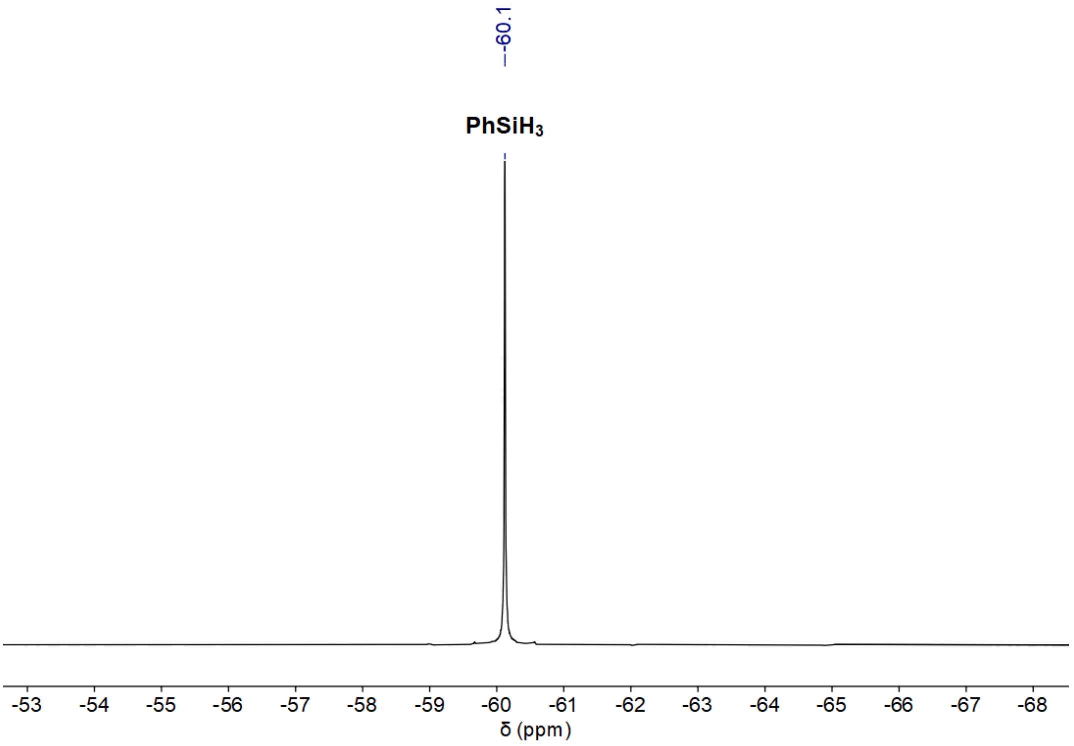

Figure S 47. ^{29}Si NMR spectrum of the reaction. Conditions: room temperature, 1.85 mmol $PhSiH_3$, 0.37 mmol tetramethylethylenediamine (25 °C, benzene-d_6, 79.49 MHz).

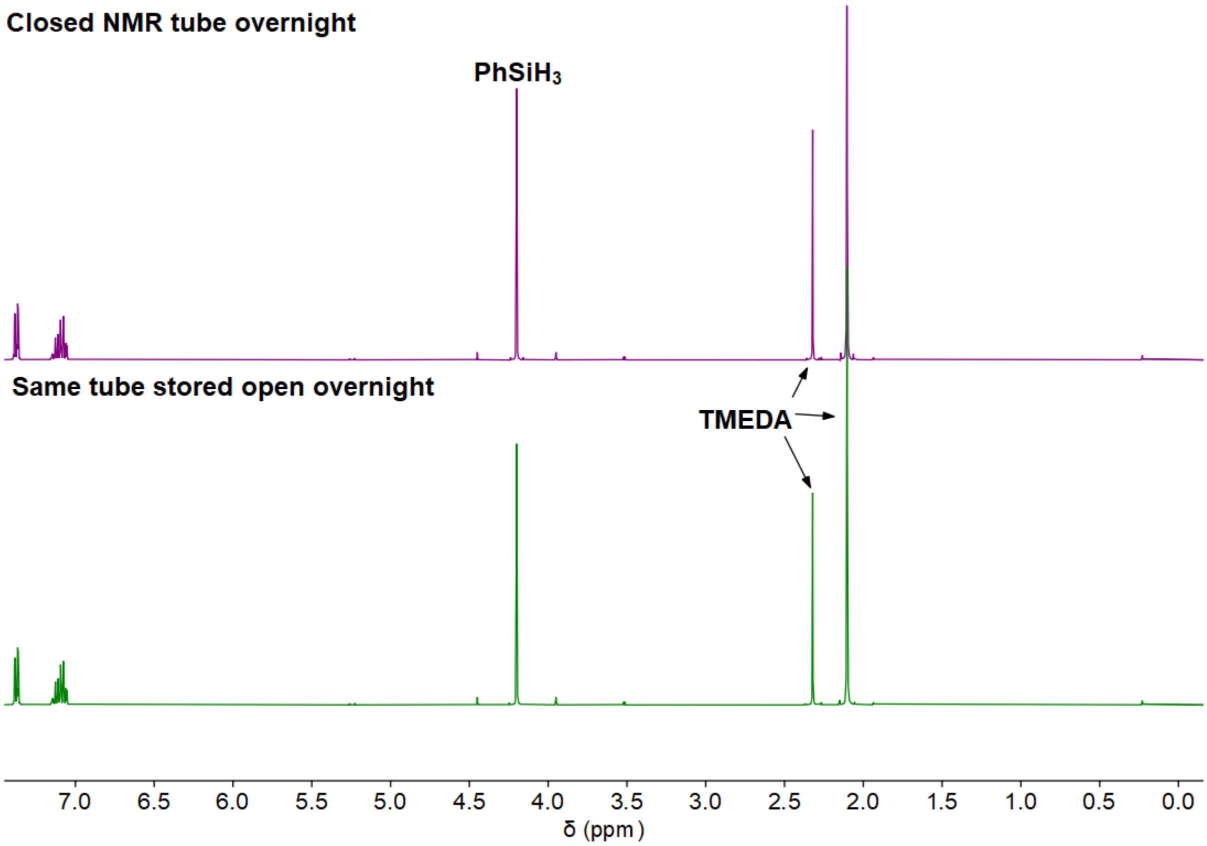

Figure S 48. ^{1}H NMR spectra of the reaction. Conditions: room temperature, 1.85 mmol $PhSiH_3$, 0.37 mmol tetramethylethylenediamine (25 °C, benzene-d_6, 400.13 MHz).

2.22. NMR spectrum of catalytic reaction of Ph_2SiH_2 using 2

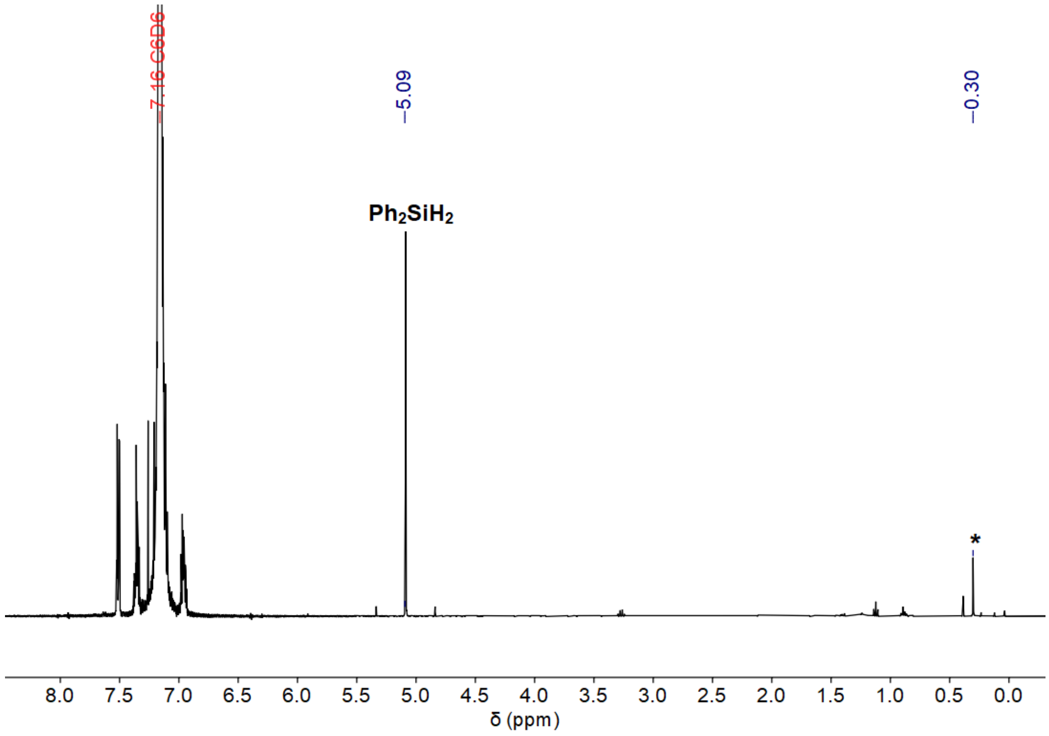

Figure S 49. ^{1}H NMR spectrum of dehydrocoupling experiment using Ph_2SiH_2, asterisk marks silicon grease. Conditions: 40 °C, open system with pressure compensation, benzene, 5.0 mol% **2** (25 °C, benzene-d_6, 400.13 MHz).

2.23. NMR spectrum of catalytic reaction of Et_2SiH_2 using 2

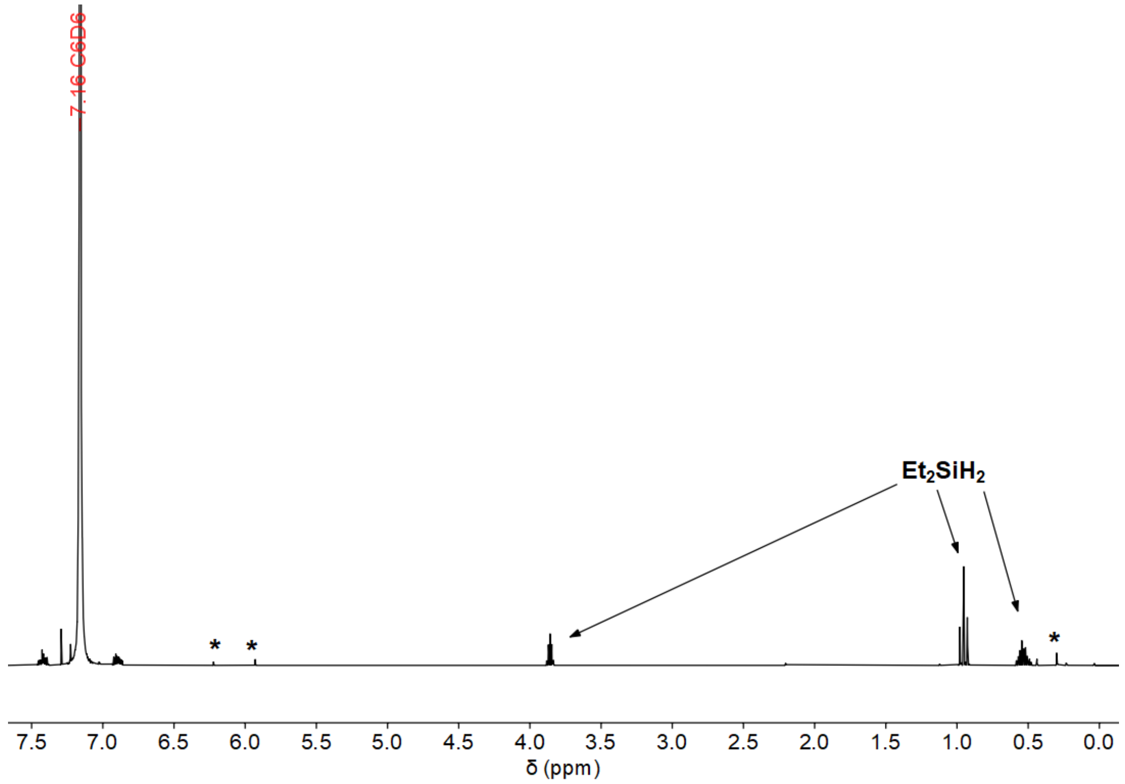

Figure S 50. ^{1}H NMR spectrum of dehydrocoupling experiment using Et_2SiH_2, asterisks mark decomposition products. Conditions: 40 °C, open system with pressure compensation, benzene, 5.0 mol% **2** (25 °C, benzene-d_6, 300.20 MHz).

2.24. NMR spectrum of catalytic reaction of *n*-$BuSiH_3$ using 2

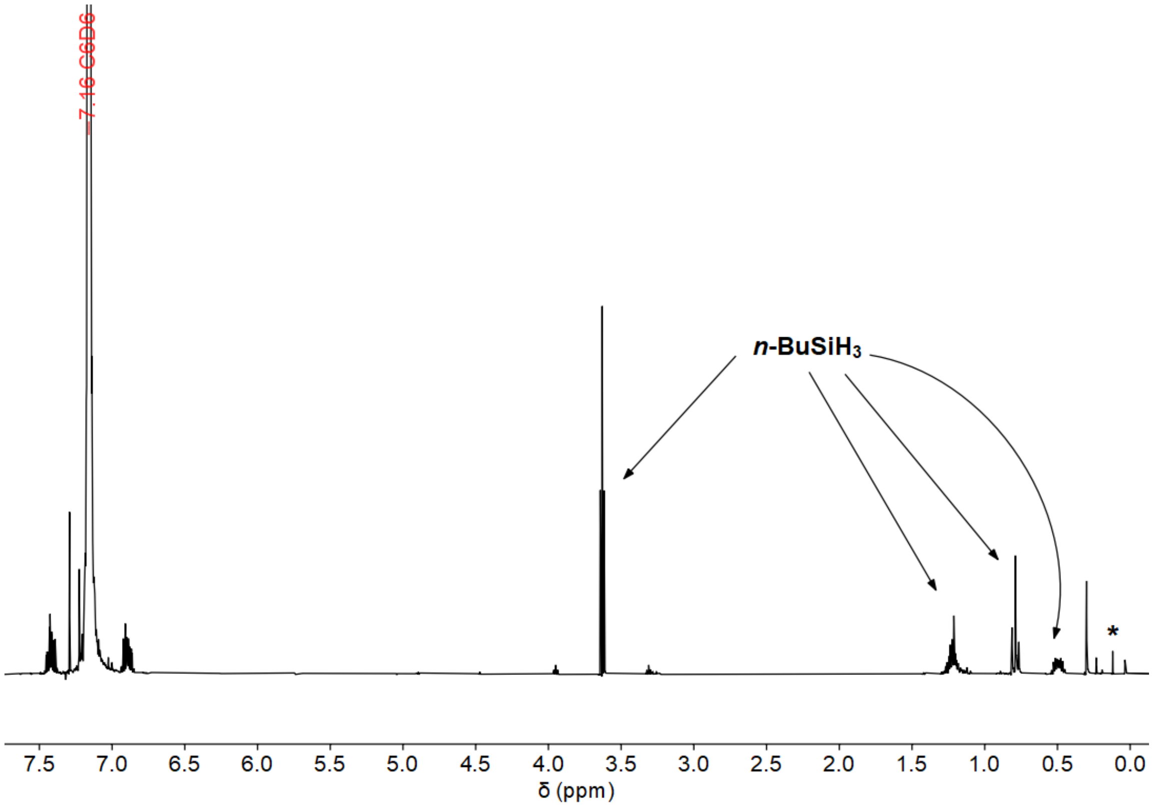

Figure S 51. ^{1}H NMR spectrum of dehydrocoupling experiment using *n*-$BuSiH_3$, asterisk marks decomposition products. Conditions: 40 °C, open system with pressure compensation, benzene, 5.0 mol% **2** (25 °C, benzene-d_6, 300.20 MHz).

3. Volumetric studies

3.1. Dehydropolymerisation of $PhSiH_3$ using different concentrations of 1

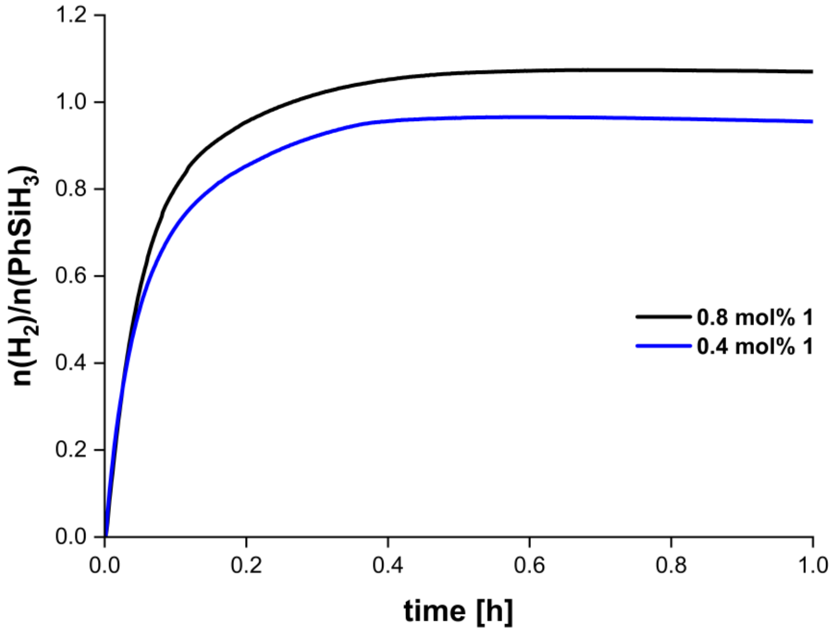

Figure S 52. Volumetric curves of dehydropolymerisation of $PhSiH_3$ with **1**. Conditions: room temperature, 1.85 mmol $PhSiH_3$.

3.2. Dehydropolymerisation of $PhSiH_3$ using *rac*-4

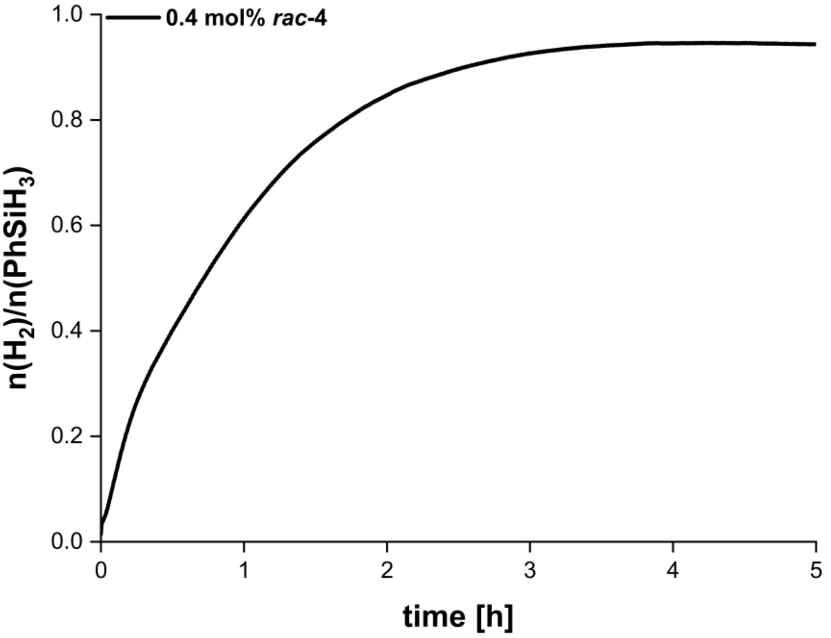

Figure S 53. Volumetric curve of dehydropolymerisation of $PhSiH_3$ with ***rac*-4**. Conditions: room temperature, 1.85 mmol $PhSiH_3$.

4. Crystallographic details

Table S 1. Crystallographic details of ***rac*-3** and ***meso*-4**

	***rac*-3**	***meso*-4**
Chem. Formula	$C_{24}H_{36}N_2Zr$	$C_{22}H_{30}ClNZr$
Form. Wght [g mol^{-1}]	443.77	435.14
Colour	yellow	yellow
Cryst. system	orthorhombic	orthorhombic
Space group	*Pbca*	$P2_12_12_1$
a [Å]	9.8199(3)	11.0777(3)
b [Å]	13.2654(4)	12.1942(4)
c [Å]	32.3207(9)	44.1788(14)
α [°]	90	90
β [°]	90	90
γ [°]	90	90
V [Å^3]	4210.3(2)	5967.8(3)
Z	8	12
$\rho_{calc.}$ [g cm^{-3}]	1.400	1.453
μ [mm^{-1}]	0.533	0.692
T [K]	150(2)	200(2)
radiation type	Mo Kα	Mo Kα
reflections measured	36367	113014
independent reflections	5484	15672
observed reflections with $I > 2\sigma(I)$	4575	15088
$R_{int.}$	0.0309	0.0314
F(000)	1872	2712
R_1 ($I > 2\sigma(I)$)	0.0276	0.0283
wR_2 (all data)	0.0707	0.0661
GOF on F^2	1.049	1.095
Parameters	248	720
CCDC number	2181386	2181387

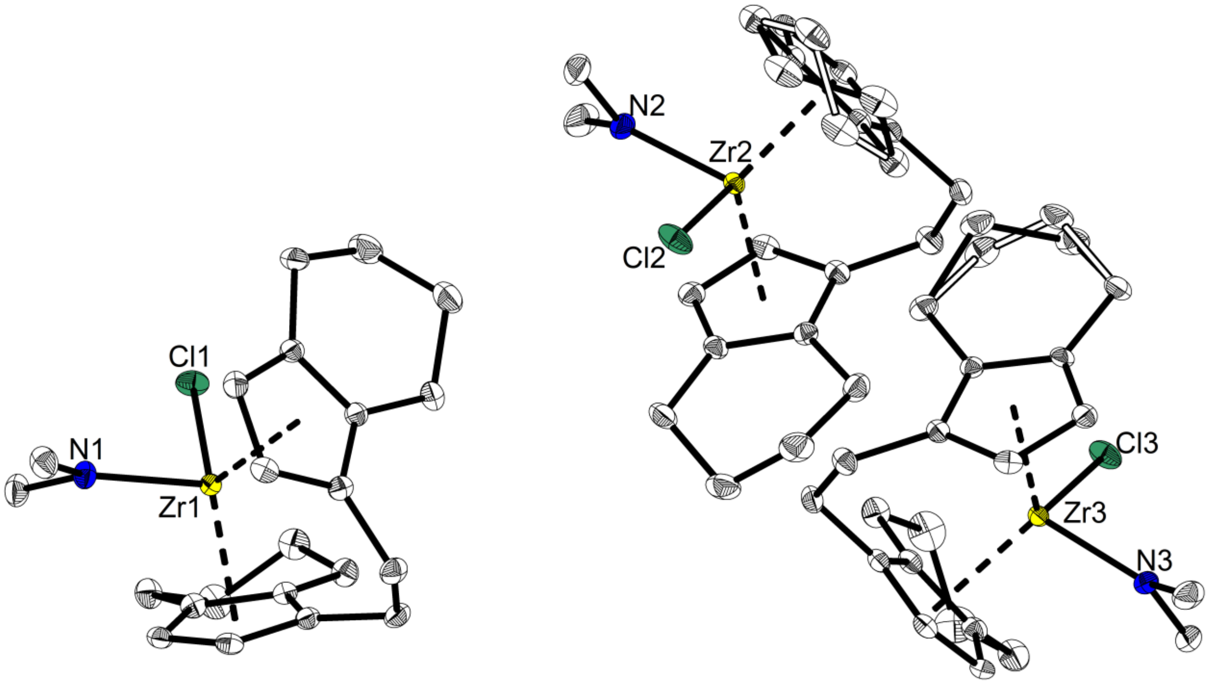

Figure S 54. Molecular structure of ***meso*-4**. Illustrated was the asymmetric unit with three isomers and disorders. The lower occupied parts of the disorder are shown with unfilled bonds. Thermal ellipsoids correspond to 30% probability. Hydrogen atoms are omitted for clarity.

5. Vibrational spectroscopy

In the following chapter the experimental infrared and Raman spectra are reported. Furthermore, selected significant bands were assigned to molecule-specific vibrations. The assignment of the vibrations was done with the help of the uncorrected calculated vibration spectra at the B3LYP/GD3BJ/def2svpp level of the theory (further details see in Chapter 7).

Table S 2. Assignment of selected infrared vibrations values are reported in wavenumbers [cm^{-1}].

complex	exp.	calc. δ $H_3\mathit{CNC}H_3$	exp.	calc. δ CH (ebthi)	exp.	calc. ν $H_3\mathit{CNC}H_3$	exp.	calc. CH_3 wagging $(N(CH_3)_2)$[a]
***rac*-3**	524 (m)	531	771 (s)	806	938 (s)	992	1130 (m)	1197
***rac*-4**	530 (m)	539	783 (s)	820	940 (s)	981	1134 (m)	1198
***meso*-4**	532 (m)	537	791 (s)	809	942 (s)	982	1134 (m)	1199

[a] highly coupled with strong $H_3\mathit{CNC}H_3$ ν_{as} vibration.

Table S 3. Assignment of selected infrared vibrations values are reported in wavenumbers [cm^{-1}].

complex	exp.	calc. CH_3 wagging $(N(CH_3)_2)$[b]	exp.	calc. δ CH_2 (ebthi)	exp.	calc. ν CH_3 $(N(CH_3)_2)$	exp.	calc. ν CH (ebthi)
***rac*-3**	1233 (w)	1273-1278	1418 (w)-1439 (w)	1461-1477	2756 (m)	2906	3065 (m)	3240
***rac*-4**	1237 (m)	1280	1435 (m)	1454-1477	2762 (m)	2923	3065 (m)	3221
***meso*-4**	1235 (m)	1280	1424 (m)-1437 (m)	1455-1473	2766 (m)	2930	3075 (w)	3241

[b] highly coupled with strong $H_3\mathit{CNC}H_3$ ν_s vibration.

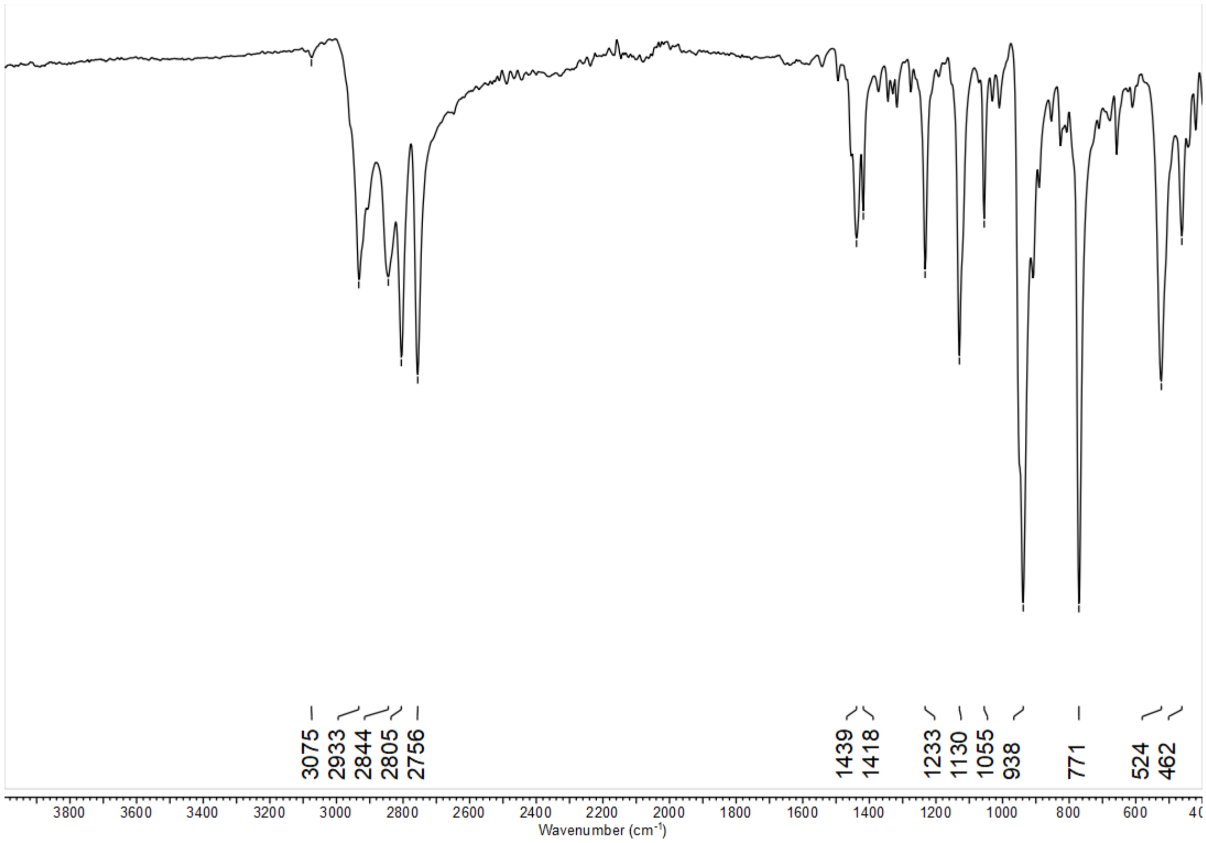

Figure S 55. IR spectrum of ***rac*-3**.

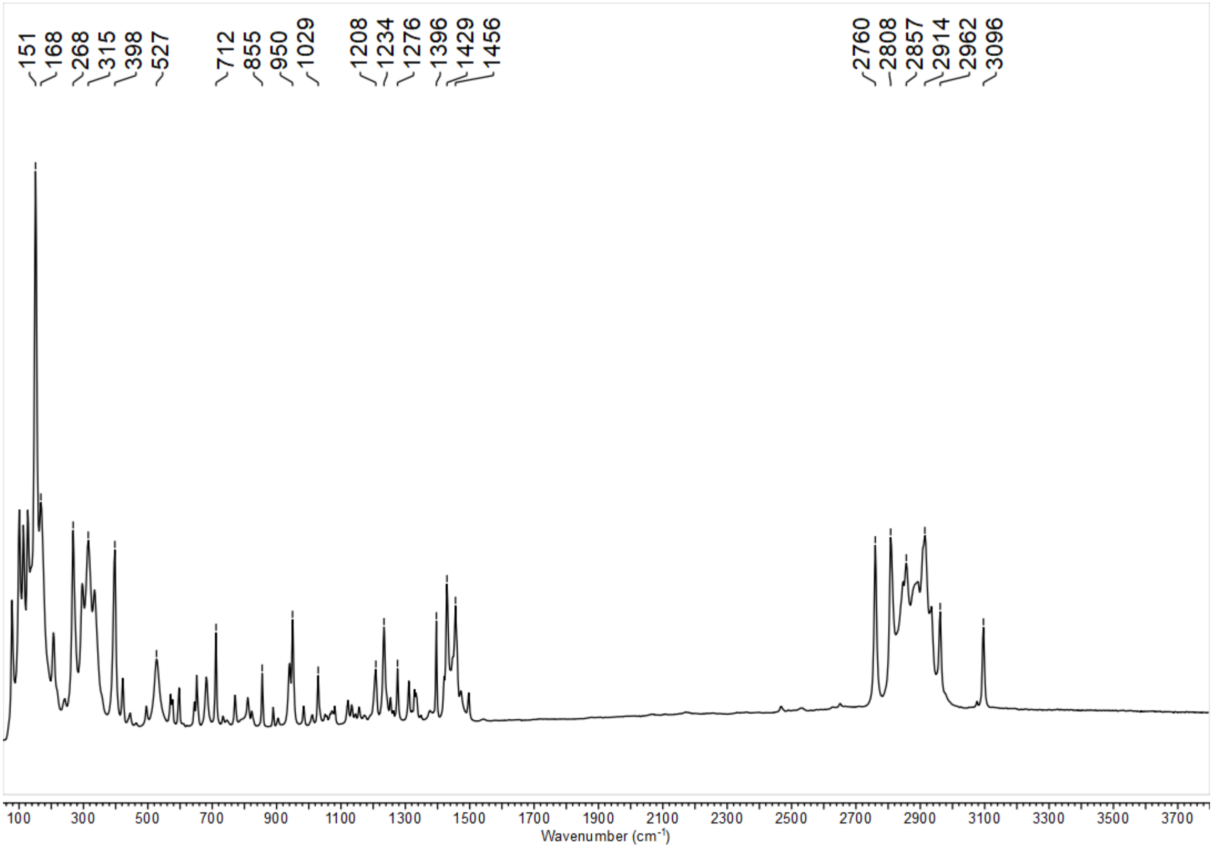

Figure S 56. Raman spectrum of ***rac*-3**.

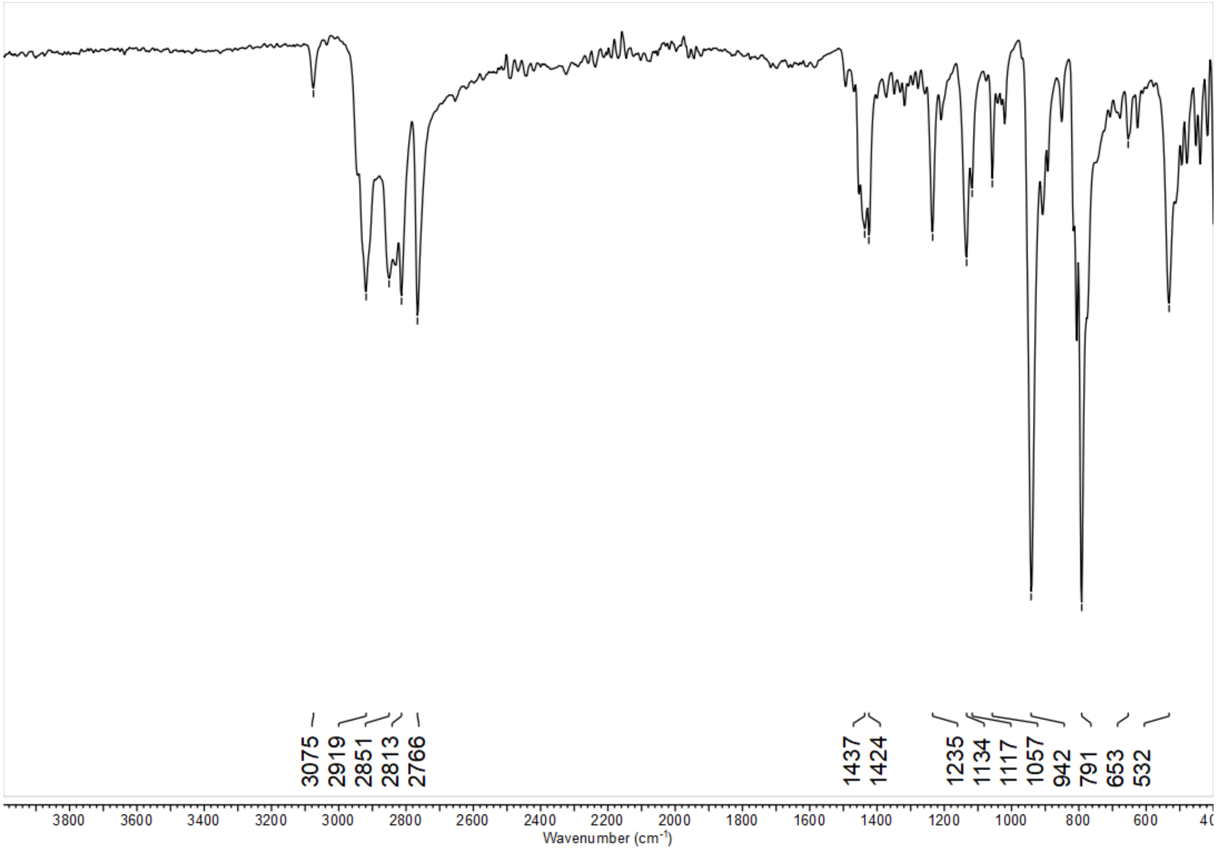

Figure S 57. IR spectrum of ***meso*-4**.

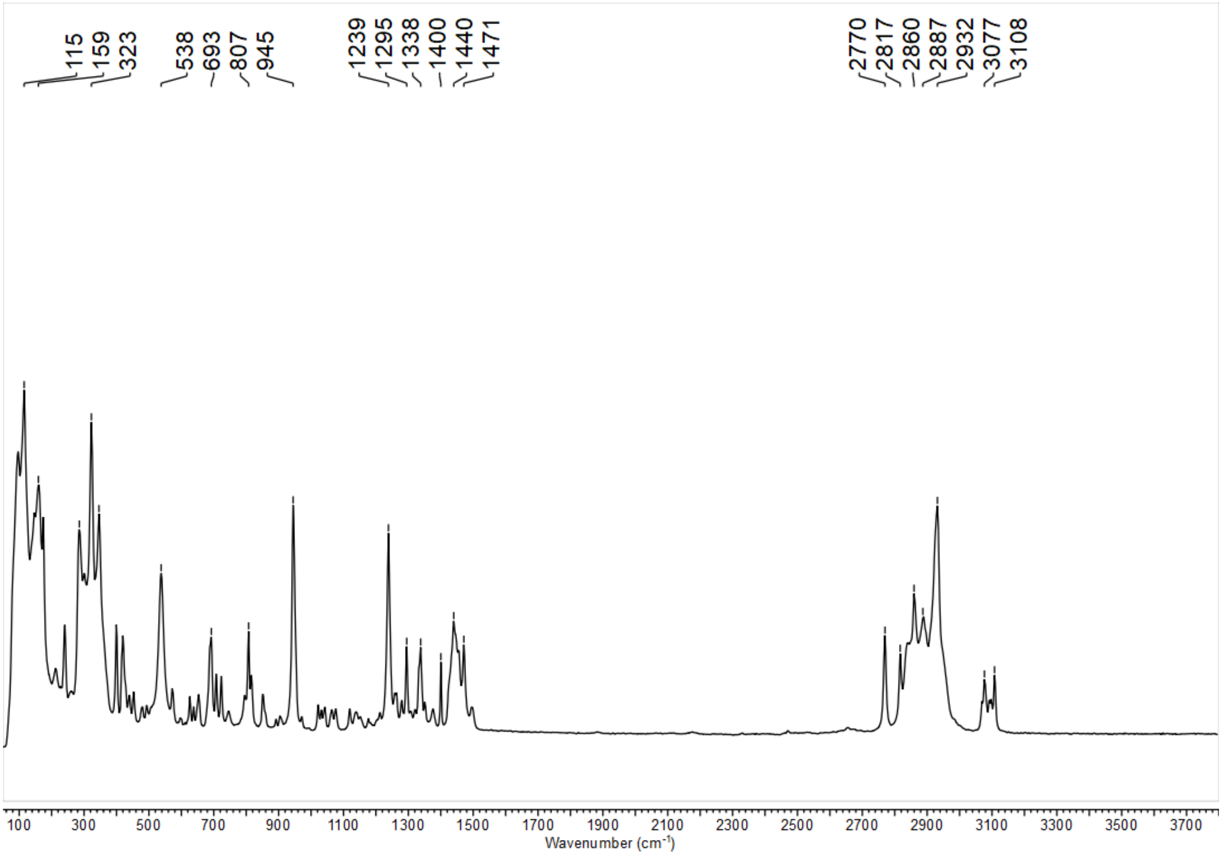

Figure S 58. RAMAN spectrum of ***meso*-4**.

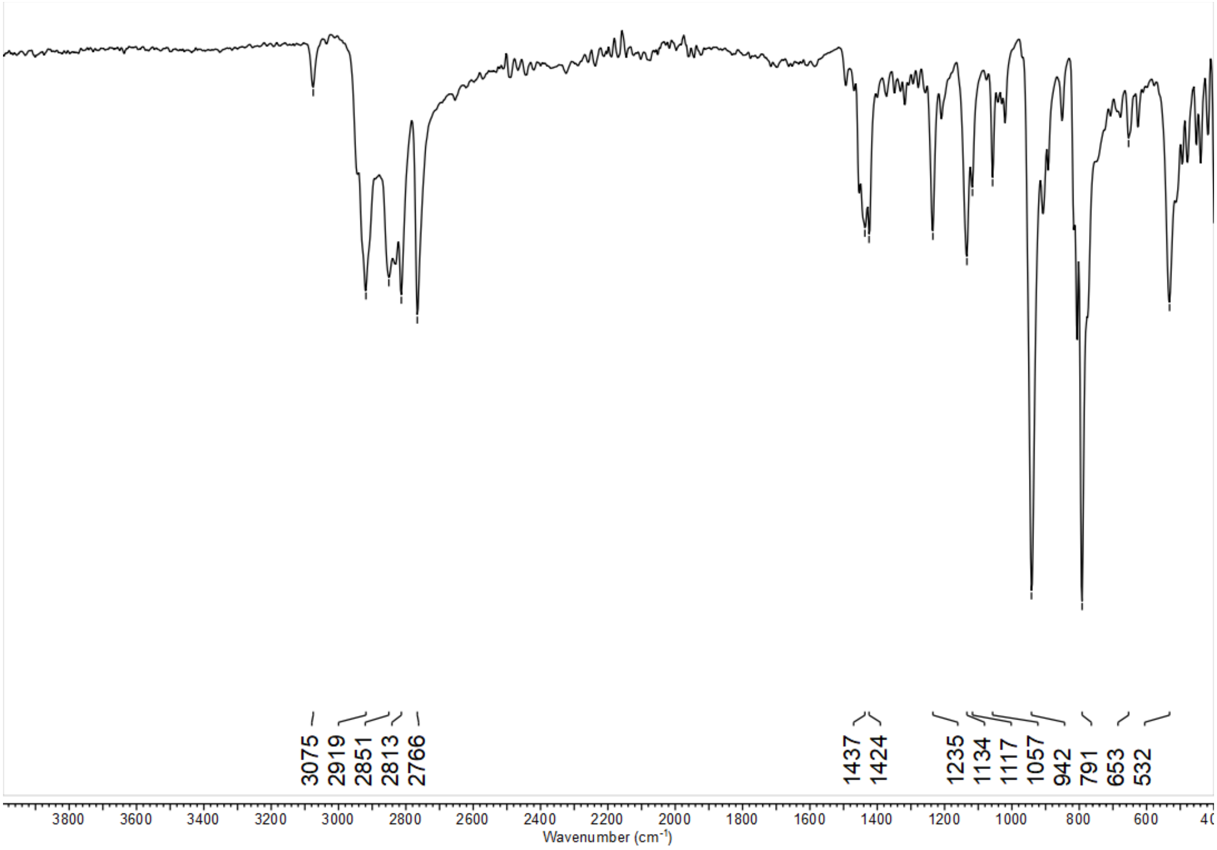

Figure S 59. IR spectrum of ***rac*-4**.

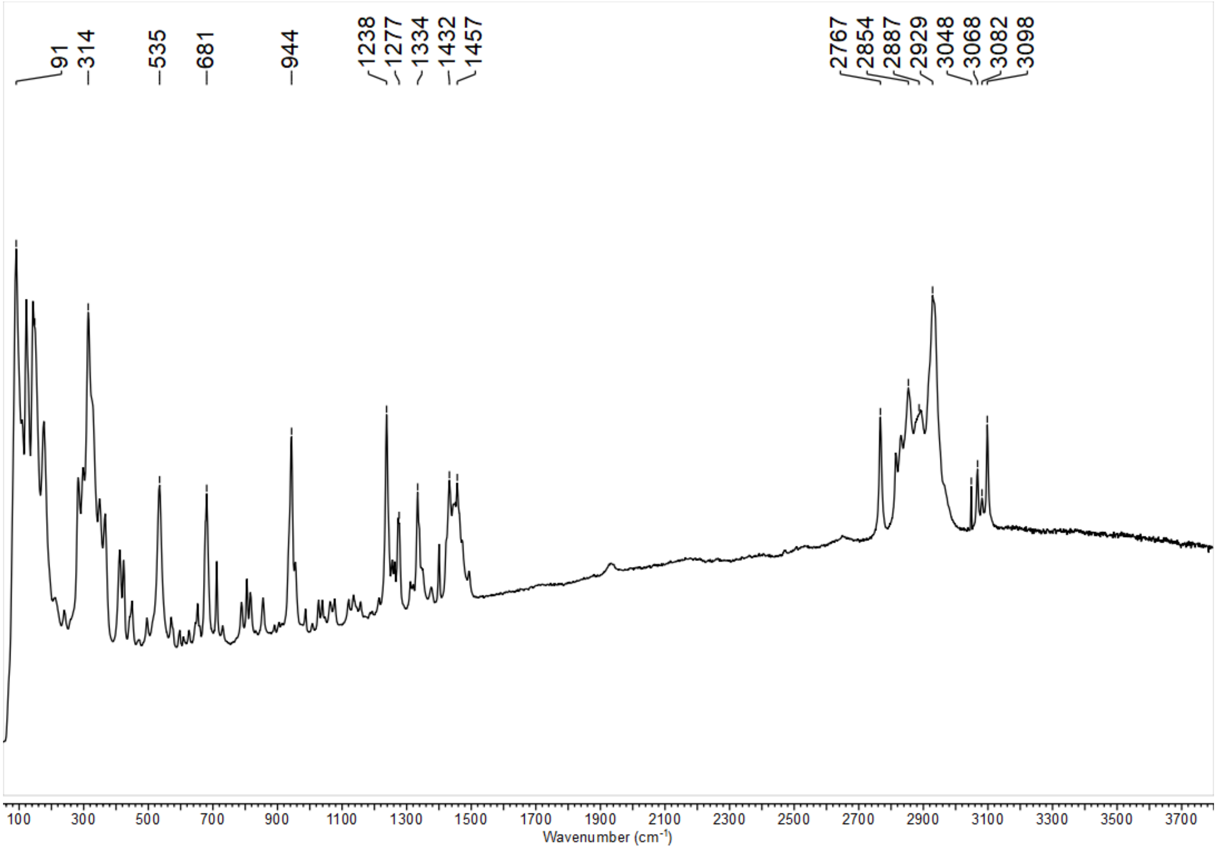

Figure S 60. RAMAN spectrum of ***rac*-4**.

6. SEC analysis

6.1. SEC analysis of polymer from dehydropolymerisation of $PhSiH_3$ using 1

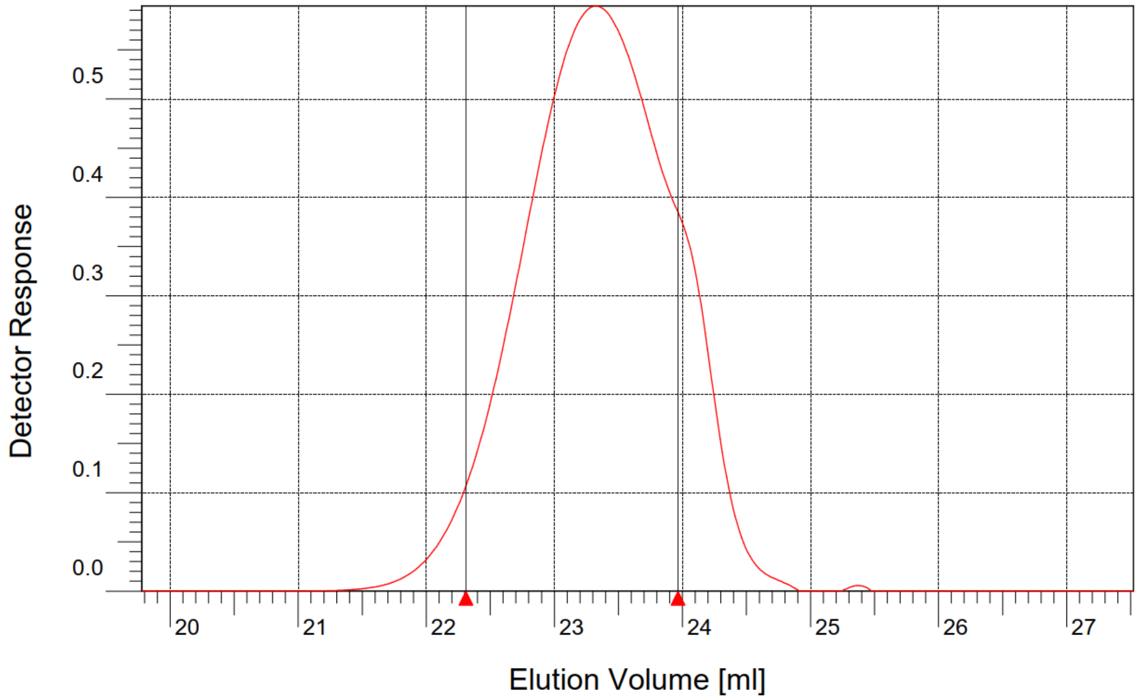

Figure S 61. Size-exclusion chromatogram of isolated polymer from dehydropolymerisation of $PhSiH_3$. Conditions: room temperature, 1.85 mmol $PhSiH_3$, 0.4 mol% **1**.

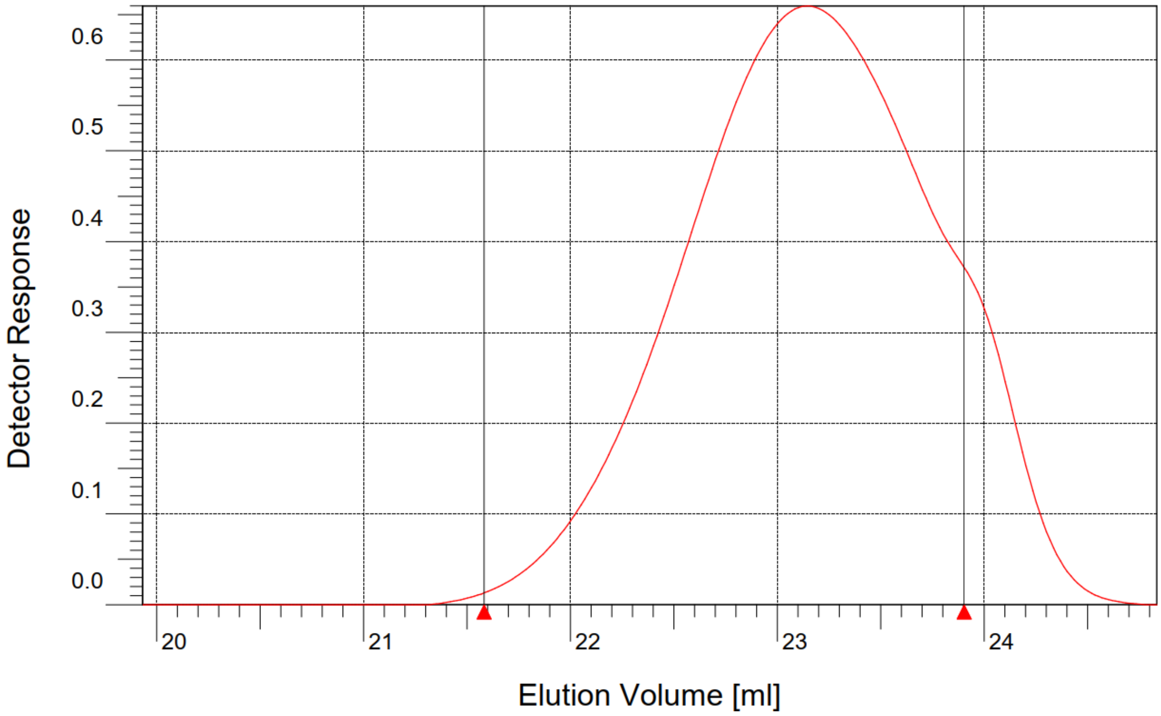

Figure S 62. Size-exclusion chromatogram of isolated polymer from dehydropolymerisation of $PhSiH_3$. Conditions: room temperature, 1.85 mmol $PhSiH_3$, 0.8 mol% **1**.

6.2. SEC analysis of polymer from dehydropolymerisation of $PhSiH_3$ using 2

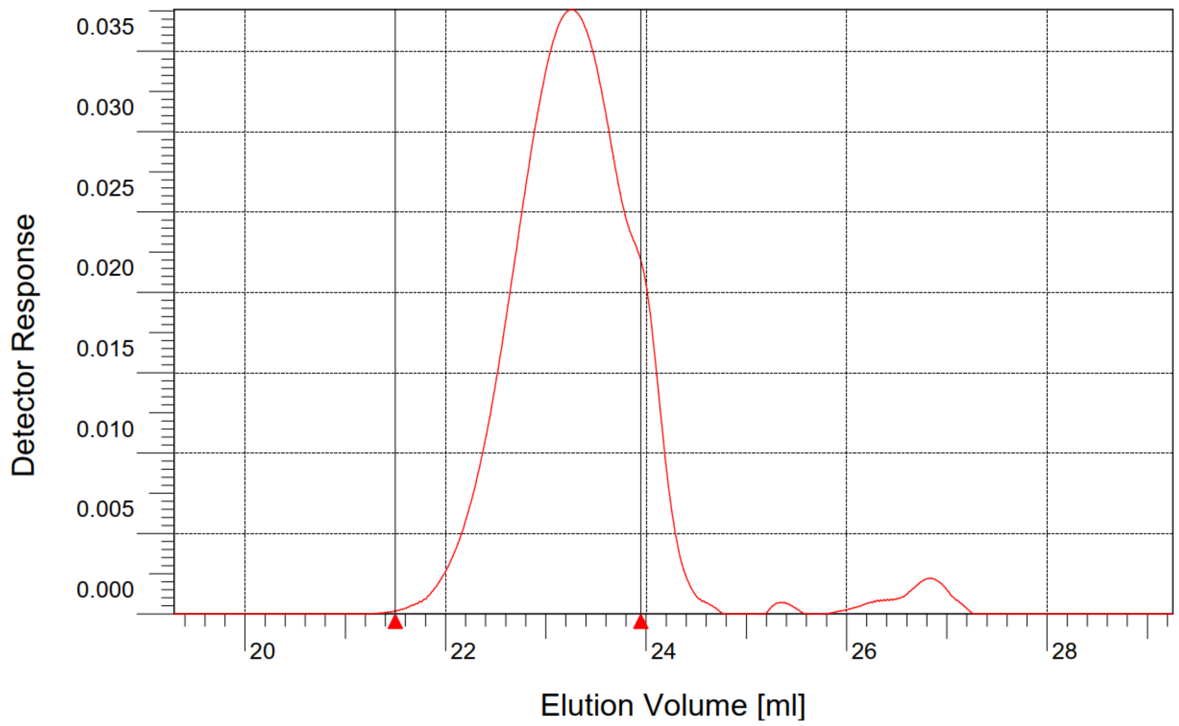

Figure S 63. Size-exclusion chromatogram of isolated polymer from dehydropolymerisation of $PhSiH_3$. Conditions: room temperature, 6.47 mmol $PhSiH_3$**,** 0.1 mol% **2**.

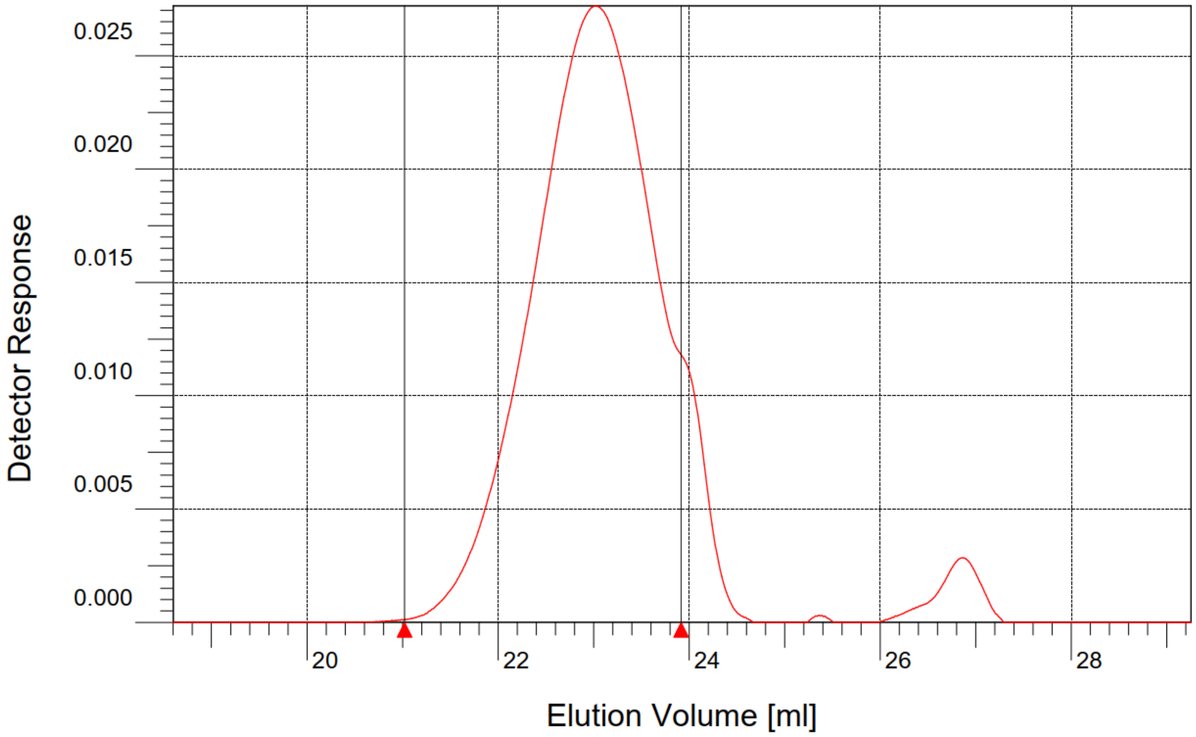

Figure S 64. Size-exclusion chromatogram of isolated polymer from dehydropolymerisation of $PhSiH_3$. Conditions: room temperature, 6.47 mmol $PhSiH_3$, 0.2 mol% **2**.

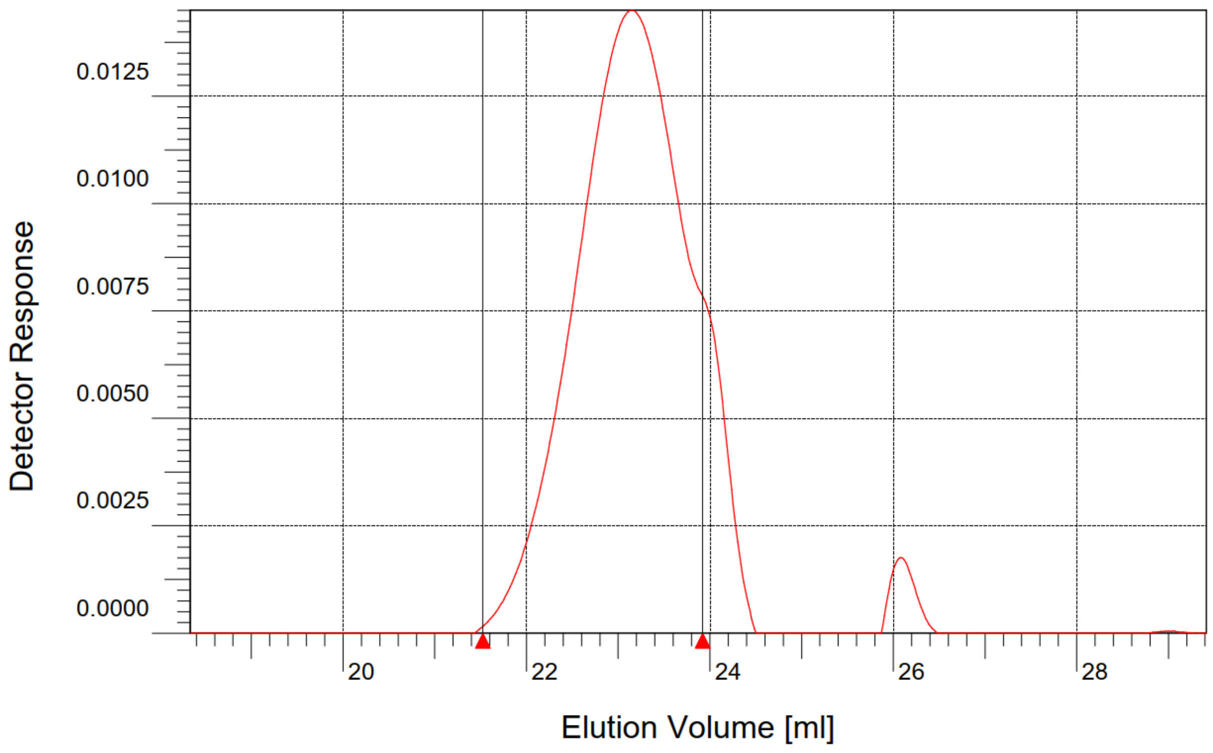

Figure S 65. Size-exclusion chromatogram of isolated polymer from dehydropolymerisation of $PhSiH_3$. Conditions: room temperature, 6.47 mmol $PhSiH_3$, 0.4 mol% **2**.

6.3. SEC analysis of isolated polymer from dehydropolymerisation experiments of $PhSiH_3$ using 2, with addition of a second portion of $PhSiH_3$

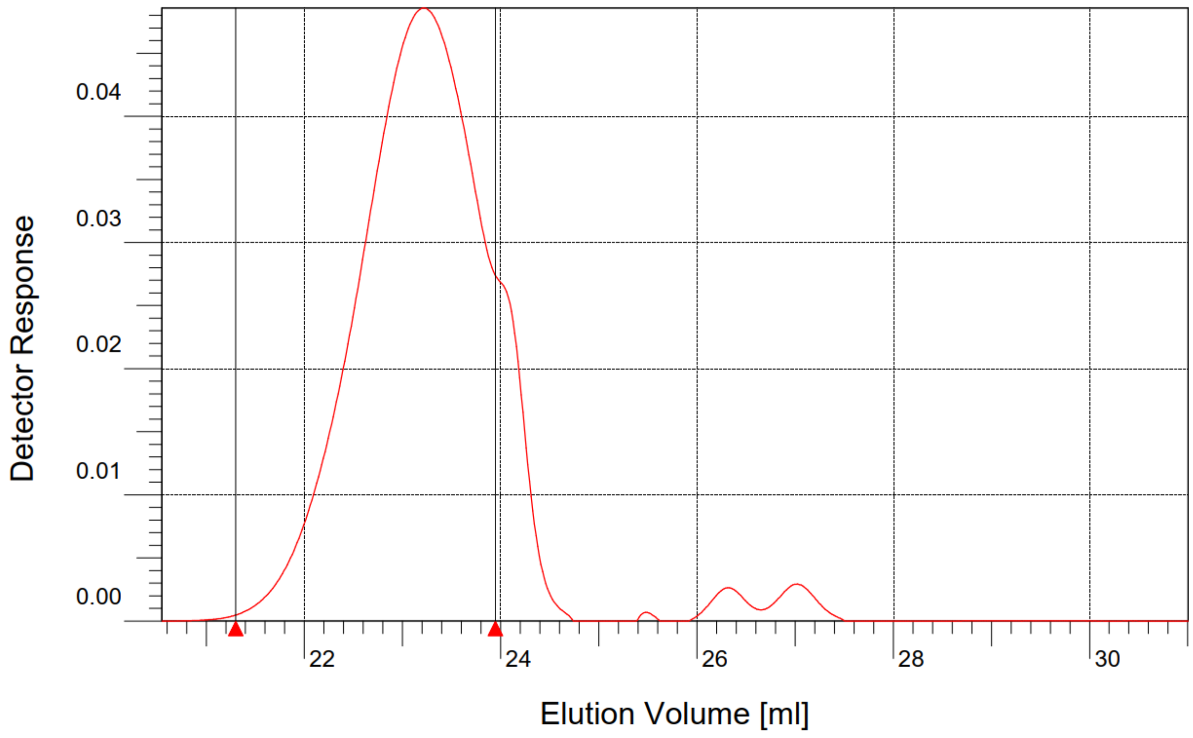

Figure S 66. Size-exclusion chromatogram of isolated polymer from catalytic dehydropolymerisation of $PhSiH_3$. Conditions: room temperature, 1.85 mmol $PhSiH_3$, 0.4 mol% **2**.

6.4. SEC analysis of isolated polymer from variable-time dehydropolymerisation experiments

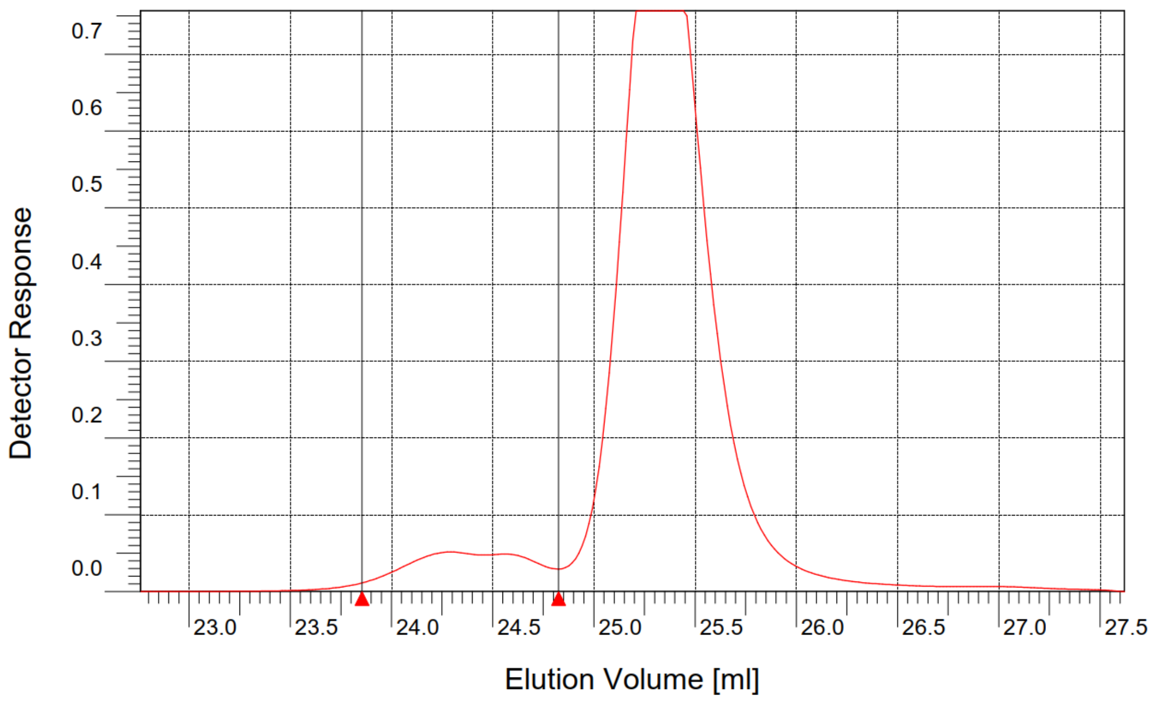

Figure S 67. Size-exclusion chromatogram of isolated polymer from an experiment stopped after 60 minutes. Conditions: room temperature, 1.85 mmol $PhSiH_3$, 0.2 mol% **2**.

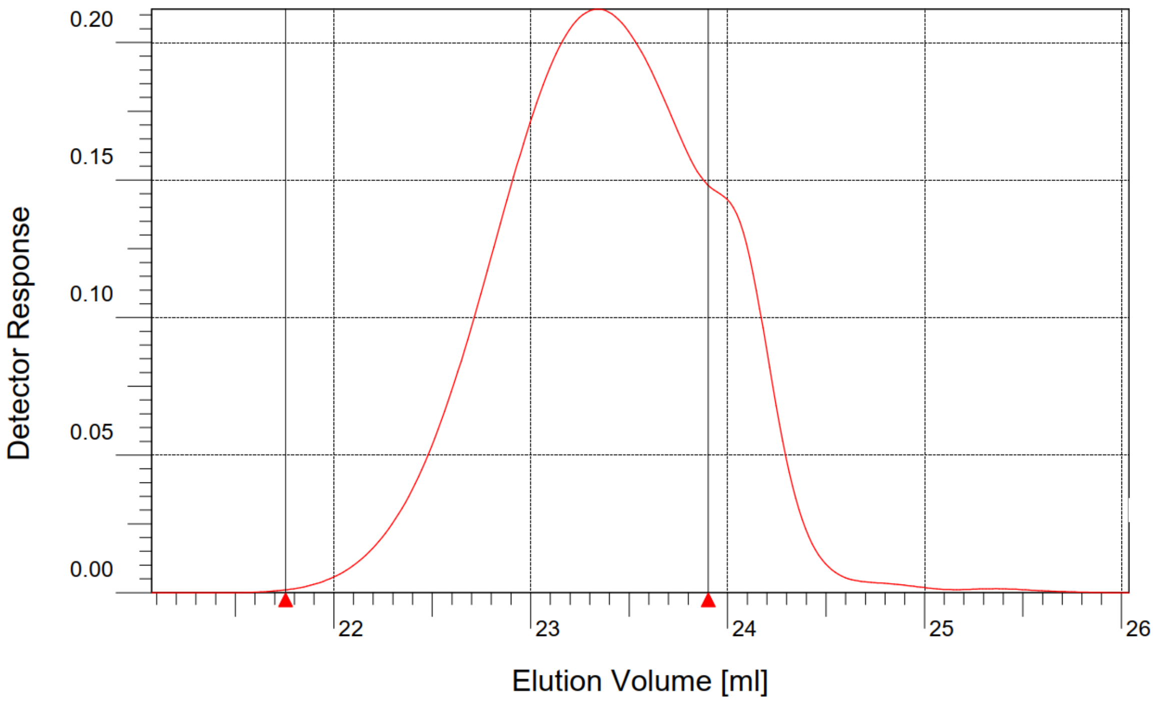

Figure S 68. Size-exclusion chromatogram of isolated polymer from an experiment stopped after 90 minutes. Conditions: room temperature, 1.85 mmol $PhSiH_3$, 0.2 mol% **2**.

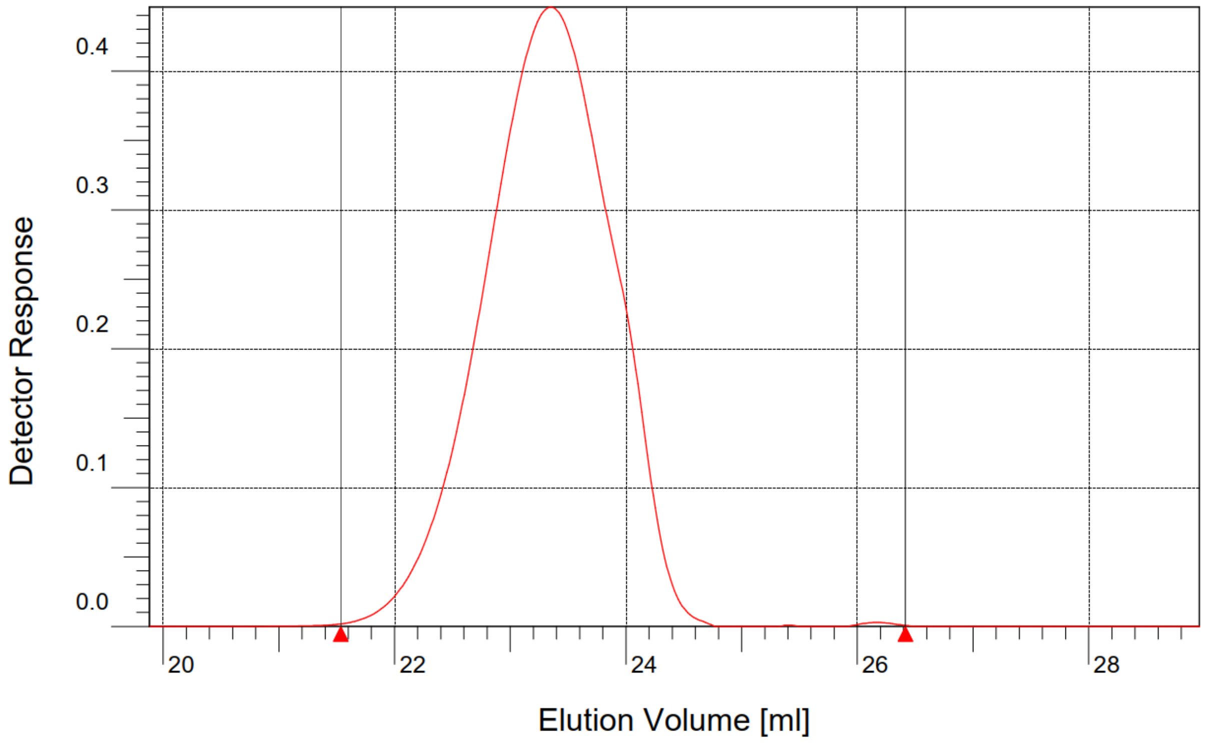

Figure S 69. Size-exclusion chromatogram of isolated polymer from an experiment at full conversion. Conditions: room temperature, 1.85 mmol $PhSiH_3$, 0.2 mol% **2**.

6.5. SEC analysis of isolated polymer from dehydropolymerisation of $PhSiH_3$ using *rac*-3

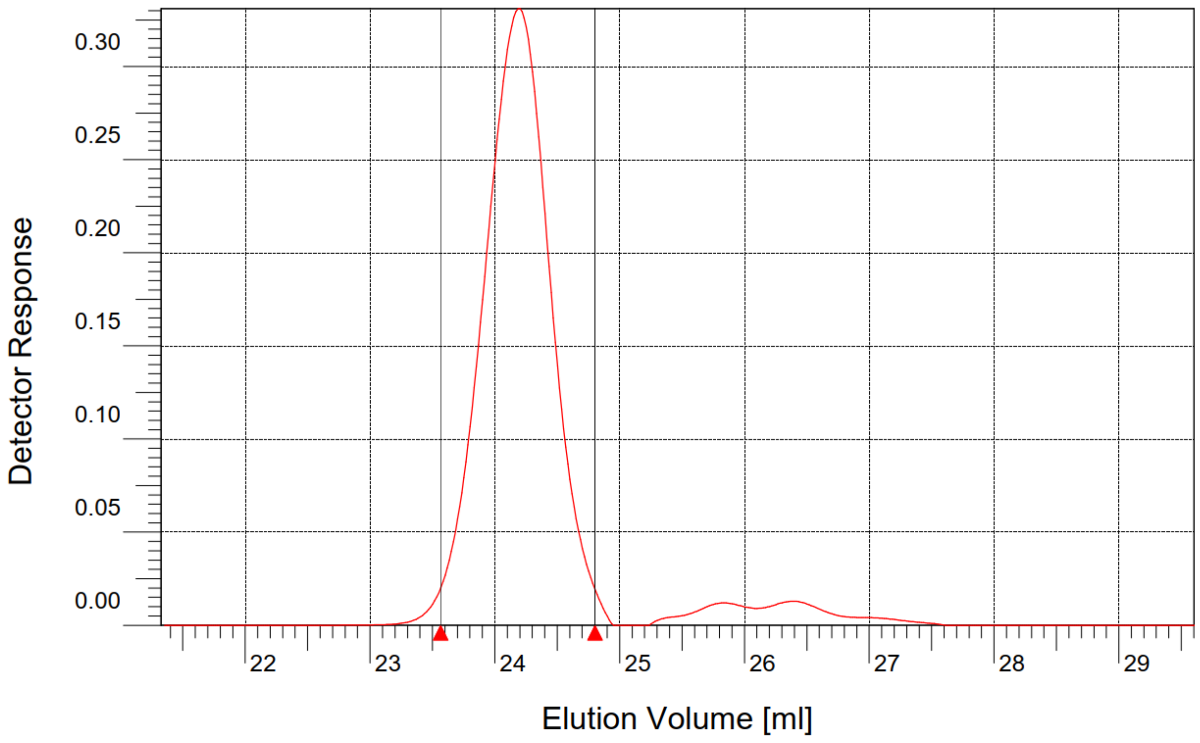

Figure S 70. Size-exclusion chromatogram of isolated polymer from dehydropolymerisation of $PhSiH_3$. Conditions: 60 °C, 1.85 mmol $PhSiH_3$, 0.8 mol% ***rac*-3**.

6.6. SEC analysis of isolated polymer from the dehydropolymerisation of $PhSiH_3$ using *rac*-4

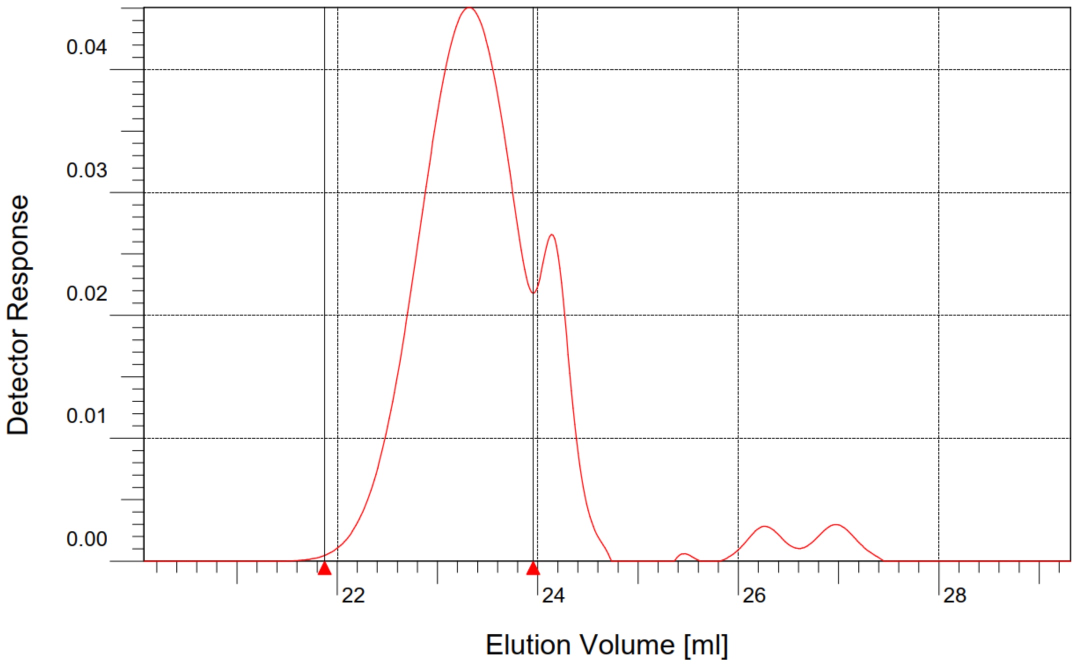

Figure S 71. Size-exclusion chromatogram of isolated polymer from dehydropolymerisation of $PhSiH_3$. Conditions: room temperature, 1.85 mmol $PhSiH_3$, 0.4 mol% ***rac*-4**.

7. Computational details

In this chapter, we give the summary of a detailed analysis of the steric demand of the ebthi amide and halide complexes. For this analysis, we used the Web Tool SambVca 2.0.[10] Since not all complexes could be analysed by SC-XRD, all structures were optimised using Gaussian 16.[11] We optimised the real-size molecules using the hybrid density functional method B3LYP,[12,13] in combination with the basis set def2svpp,[14] and the empirical dispersion correction GD3BJ[15] (notation: B3LYP/GD3BJ/def2svpp). Vibrational frequencies were computed, to include zero-point vibrational energies in thermodynamic parameters and to characterise all structures as minima on the potential energy surface. To be able to generate a coordinate system/axis system that is as uniform as possible during the subsequent buried volume analysis, a "dummy" hydrogen atom was inserted into the optimised structures with the aid of GaussView. This dummy atom was placed exactly in the middle between the hetero atoms N and Cl in each of the seven complexes studied and was deleted again during the analysis. Generation of the respective coordinate system with the help of this dummy atom is documented for each molecule. For the calculation of thermodynamic relative energies of the different isomers we furthermore performed single point calculations on triple-ζ basis (def2tzvp) to obtain more accurate values.

In addition to the electronic supporting information, we provide a multi-structure xyz-file including all calculated molecules.

Please note that all computations were carried out for single, isolated molecules in the gas phase (ideal gas approximation). There may well be significant differences between gas phase and condensed phase.

7.1. Thermodynamic considerations of the isomerism between *rac*-3 and *meso*-3 as well as two different isomers of *meso*-4

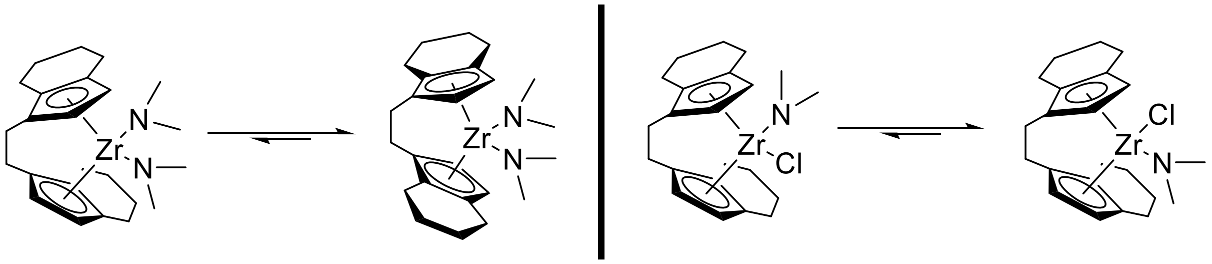

***meso*-4** ***rac*-3** ***meso*-4** iso2 ***meso*-4** iso1

Investigated isomerism	$\Delta_R H$ [kJ/mol]	$\Delta_R G$ [kJ/mol]	$\Delta_R H$ [kcal/mol]	$\Delta_R G$ [kcal/mol]
rac- vs. *meso*-(ebthi)Zr(NMe_2)$_2$	-18.5	-14.8	-4.4	-3.5
meso-(ebthi)ZrClNMe_2 iso1 vs. iso2	-33.8	-32.9	-8.1	-7.9

7.2. Summary of calculated thermodynamic data

File-Name	Nimag	HF	ZPE [kcal/mo	Th. Corr. H	Th. Corr. G	H_{tot} [a.u.]	G_{tot} [a.u.]	Method	Basisset	Cacl
HCl	0	-460.6619105	4.06516	0.009783	-0.011433	-460.652128	-460.673343	B3LYP/GD3BJ	def2svpp	opt/freq
HNMe2	0	-135.0547443	57.67045	0.097218	0.066479	-134.957526	-134.988265	B3LYP/GD3BJ	def2svpp	opt/freq
racEBTHIZrCl2	0	-1743.554177	247.764	0.416944	0.346303	-1743.137233	-1743.207874	B3LYP/GD3BJ	def2svpp	opt/freq
mesoEBTHIZrCl2	0	-1743.554184	247.78518	0.417015	0.346224	-1743.13717	-1743.20796	B3LYP/GD3BJ	def2svpp	opt/freq
racEBTHIZrClNMe2	0	-1417.896568	298.22807	0.500396	0.424444	-1417.396172	-1417.472124	B3LYP/GD3BJ	def2svpp	opt/freq
mesoEBTHIZrClNMe2 iso1	0	-1417.898098	298.00044	0.500233	0.423697	-1417.397865	-1417.474401	B3LYP/GD3BJ	def2svpp	opt/freq
mesoEBTHIZrClNMe2 iso2	0	-1417.884589	298.09164	0.500415	0.423562	-1417.384174	-1417.461027	B3LYP/GD3BJ	def2svpp	opt/freq
racEBTHIZrNMe2_2_opt_freq_svp	0	-1092.233684	348.58168	0.58361	0.502551	-1091.650074	-1091.731132	B3LYP/GD3BJ	def2svpp	opt/freq
mesoEBTHIZrNMe2_2_opt_freq_svp	0	-1092.225566	348.19766	0.583331	0.500858	-1091.642235	-1091.724708	B3LYP/GD3BJ	def2svpp	opt/freq
HCl	-	-460.835443	data tken from def2svpp opt freq			-460.82566	-460.846876	B3LYP/GD3BJ	def2tzvp	sp
HNMe2	-	-135.2293904	data tken from def2svpp opt freq			-135.1321724	-135.1629114	B3LYP/GD3BJ	def2tzvp	sp
racEBTHIZrCl2	-	-1744.764653	data tken from def2svpp opt freq			-1744.347709	-1744.41835	B3LYP/GD3BJ	def2tzvp	sp
mesoEBTHIZrCl2	-	-1744.764219	data tken from def2svpp opt freq			-1744.347204	-1744.417995	B3LYP/GD3BJ	def2tzvp	sp
racEBTHIZrClNMe2	-	-1419.112688	data tken from def2svpp opt freq			-1418.612292	-1418.688244	B3LYP/GD3BJ	def2tzvp	sp
mesoEBTHIZrClNMe2 iso1	-	-1419.114194	data tken from def2svpp opt freq			-1418.613961	-1418.690497	B3LYP/GD3BJ	def2tzvp	sp
mesoEBTHIZrClNMe2 iso2	-	-1419.101511	data tken from def2svpp opt freq			-1418.601096	-1418.677949	B3LYP/GD3BJ	def2tzvp	sp
racEBTHIZrNMe2_2	-	-1093.456256	data tken from def2svpp opt freq			-1092.872646	-1092.953705	B3LYP/GD3BJ	def2tzvp	sp
mesoEBTHIZrNMe2_2	-	-1093.448942	data tken from def2svpp opt freq			-1092.865611	-1092.948084	B3LYP/GD3BJ	def2tzvp	sp

7.3. Buried Volume Analysis of *rac*-(ebthi)ZrCl$_2$

Axis-System	Buried Volume Analysis
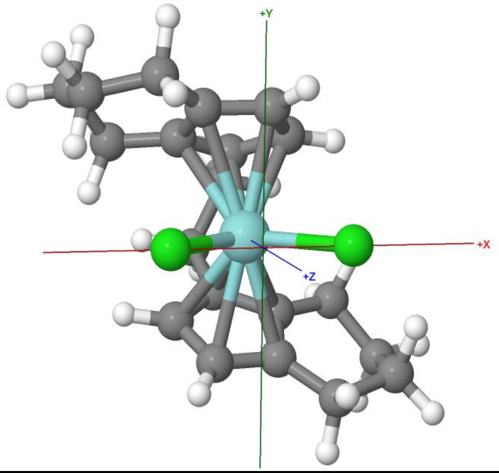	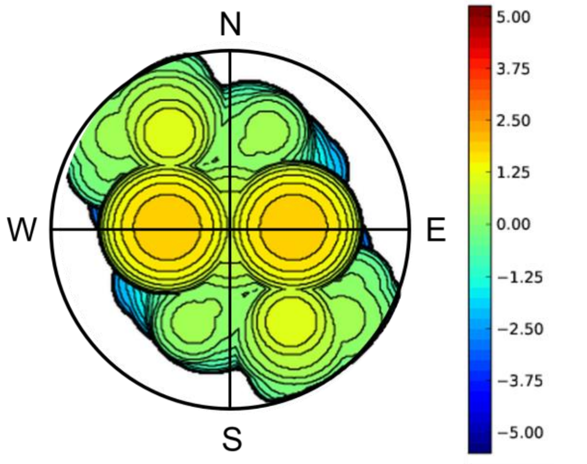

Settings:
Coordinating Atoms: Cl1, Dummy, Cl2
Z-axis defined by Zr atom z-negative
XZ-Plane defined by Cl1, Zr1, Dummy
Atomic radii scaled by 1.17
Sphere radius 5.5
Distance of coord. Point from centre 0.0
Mesh spacings for int. 0.05
H's includes

%V_{free}	%V_{bur}	%V_{tot}
55.2	44.8	100.0

Quadrant	V_{free}	V_{bur}	V_{total}	%V_{free}	%V_{bur}
SW	110.8	63.4	174.2	63.6	36.4
NW	81.4	92.8	174.2	46.7	53.3
NE	110.8	63.4	174.2	63.6	36.4
SE	81.4	92.8	174.2	46.7	53.3

7.4. Buried Volume Analysis of *meso*-(ebthi)ZrCl$_2$

Axis-System	Buried Volume Analysis
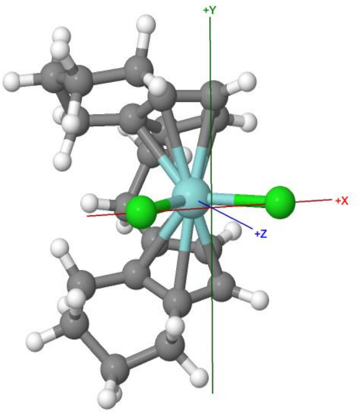	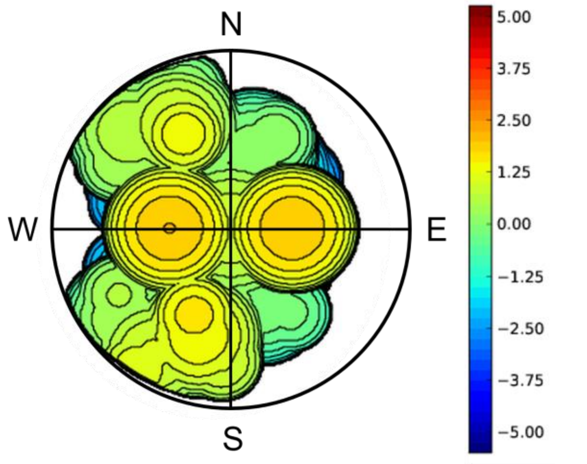

Settings:
Coordinating Atoms: Cl1, Dummy, Cl2
Z-axis defined by Zr atom z-negative
XZ-Plane defined by Cl1, Zr1, Dummy
Atomic radii scaled by 1.17
Sphere radius 5.5
Distance of coord. Point from centre 0.0
Mesh spacings for int. 0.05
H's includes

%V_{free}	%V_{bur}	%V_{tot}
53.7	46.3	100.0

Quadrant	V_{free}	V_{bur}	V_{total}	%V_{free}	%V_{bur}
SW	73.0	101.2	174.2	41.9	58.1
NW	77.5	96.7	174.2	44.5	55.5
NE	113.1	61.1	174.2	64.9	35.1
SE	110.4	63.8	174.2	63.4	36.6

7.5. Buried Volume Analysis of *rac*-3

Axis-System	Buried Volume Analysis
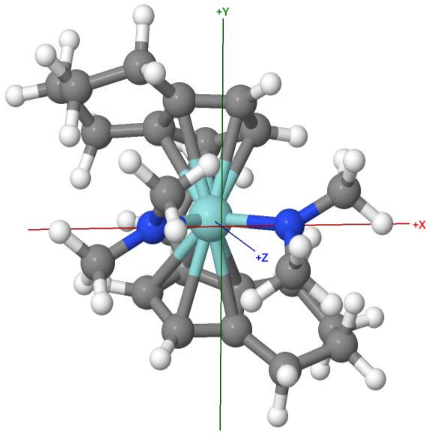	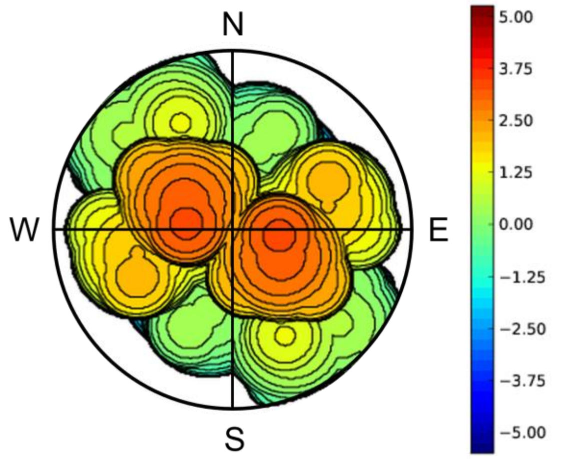

Settings:
Coordinating Atoms: N1, Dummy, N2
Z-axis defined by Zr atom z-negative
XZ-Plane defined by N1, Dummy, N2
Atomic radii scaled by 1.17
Sphere radius 5.5
Distance of coord. Point from centre 0.0
Mesh spacings for int. 0.05
H‘s includes

**%V$_{free}$**	**%V$_{bur}$**	**%V$_{tot}$**
45.0	55.0	100.0

**Quadrant**	**V$_{free}$**	**V$_{bur}$**	**V$_{total}$**	**%V$_{free}$**	**%V$_{bur}$**
SW	92.2	82.1	174.2	52.9	47.1
NW	64.5	109.7	174.2	37.1	62.9
NE	92.1	82.1	174.2	52.9	47.1
SE	64.5	109.7	174.2	37.1	62.9

7.6. Buried Volume Analysis of *meso*-3

Axis-System	Buried Volume Analysis
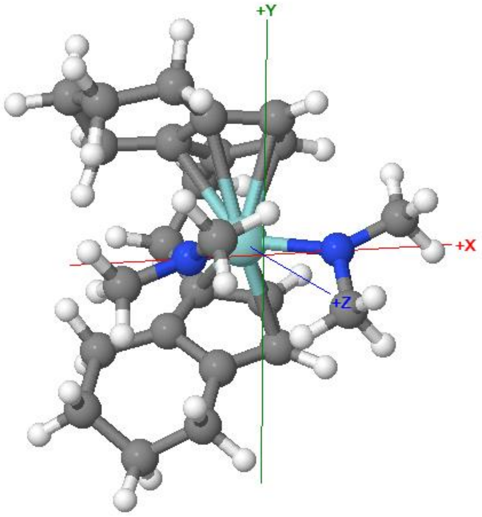	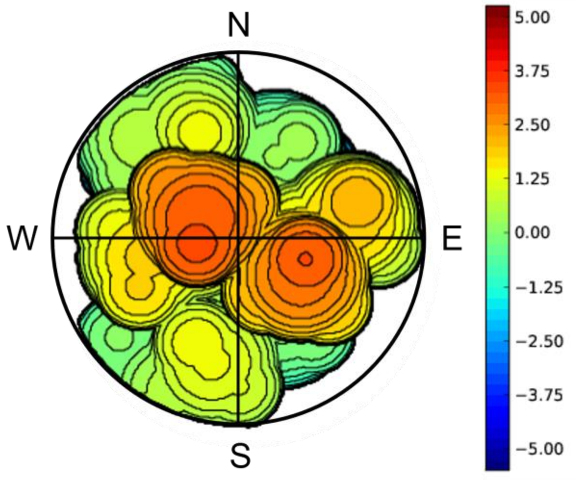

Settings:
Coordinating Atoms: N1,Dummy, N2
Z-axis defined by Zr atom z-negative
XZ-Plane defined by N1, Dummy, Zr
Dummy deleted
Atomic radii scaled by 1.17
Sphere radius 5.5
Distance of coord. Point from centre 0.0
Mesh spacings for int. 0.05
H‘s includes

**%V$_{free}$**	**%V$_{bur}$**	**%V$_{tot}$**
45.1	54.9	100.0

**Quadrant**	**V$_{free}$**	**V$_{bur}$**	**V$_{total}$**	**%V$_{free}$**	**%V$_{bur}$**
SW	64.5	109.7	174.2	37.0	63.0
NW	62.6	111.6	174.2	35.9	64.1
NE	95.5	78.7	174.2	54.8	45.2
SE	91.6	82.6	174.2	52.6	47.4

7.7. Buried Volume Analysis of N*in* Cl*out* isomer of *meso*-4

Axis-System	Buried Volume Analysis
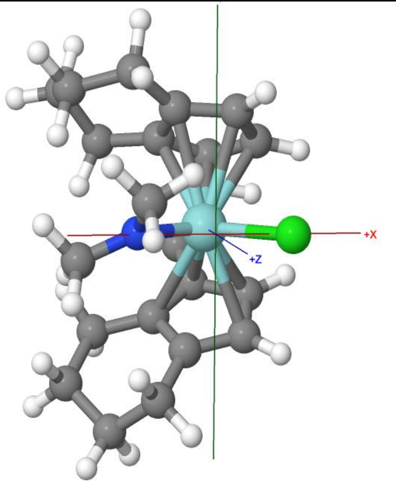	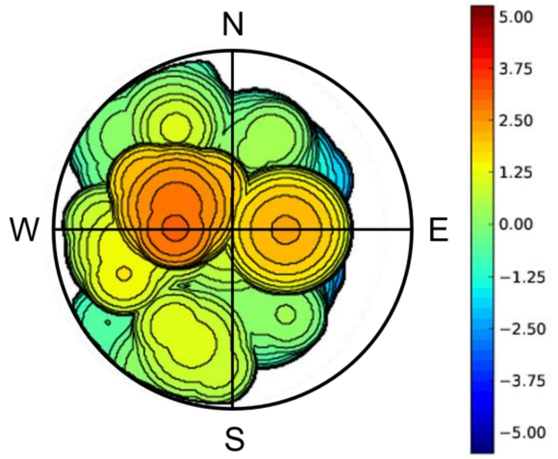

Settings:
Coordinating Atoms: N1, Dummy, Cl1
Z-axis defined by Zr atom z-negative
XZ-Plane defined by N1, Dummy, Cl1
Atomic radii scaled by 1.17
Sphere radius 5.5
Distance of coord. Point from centre 0.0
Mesh spacings for int. 0.05
H‘s includes

%V_{free}	%V_{bur}	%V_{tot}
50.9	49.1	100.0

Quadrant	V_{free}	V_{bur}	V_{total}	%V_{free}	%V_{bur}
SW	71.1	103.1	174.2	40.8	59.2
NW	68.5	105.7	174.2	39.3	60.7
NE	107.6	66.6	174.2	61.8	38.2
SE	107.2	67.0	174.2	61.6	38.4

7.8. Buried Volume Analysis of N*out* Cl*in* isomer of *meso*-4

Axis-System	Buried Volume Analysis
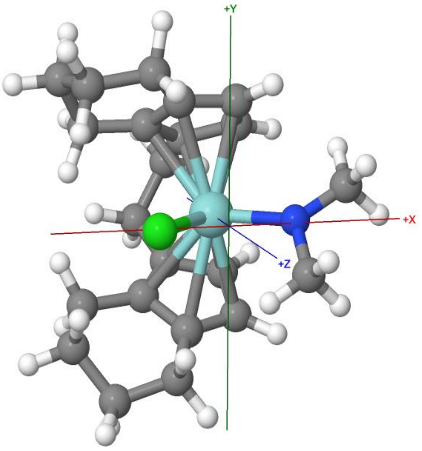	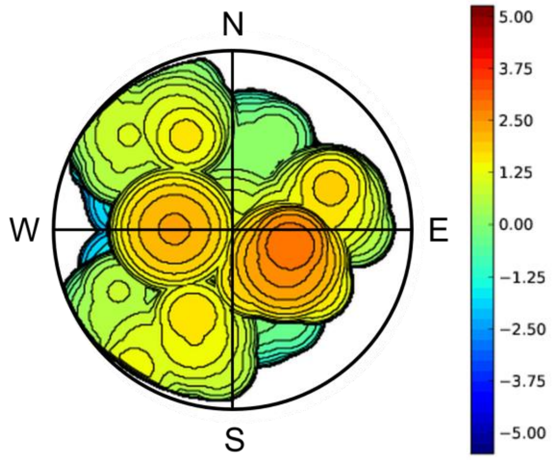

Settings:
Coordinating Atoms: N1, Dummy, Cl1
Z-axis defined by Zr atom z-negative
XZ-Plane defined by Cl1, Dummy, N1
Atomic radii scaled by 1.17
Sphere radius 5.5
Distance of coord. Point from centre 0.0
Mesh spacings for int. 0.05
H‘s includes

%V_{free}	%V_{bur}	%V_{tot}
48.4	51.6	100.0

Quadrant	V_{free}	V_{bur}	V_{total}	%V_{free}	%V_{bur}
SW	68.3	105.9	174.2	39.2	60.8
NW	73.0	101.2	174.2	41.9	58.1
NE	100.0	74.2	174.2	57.4	42.6
SE	96.1	78.1	174.2	55.2	44.8

7.9. Buried Volume Analysis of *rac*-4

Axis-System

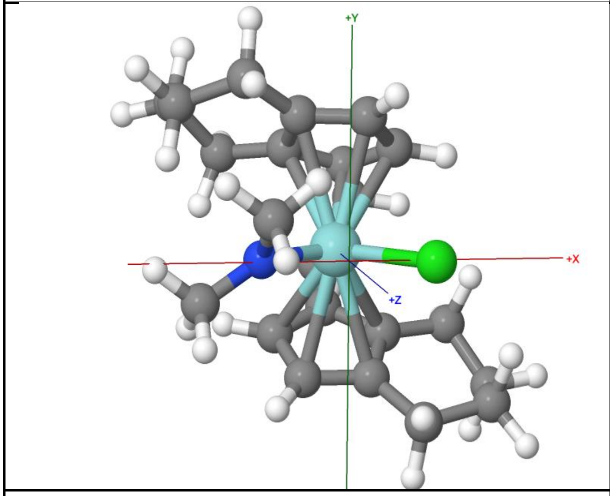

Buried Volume Analysis

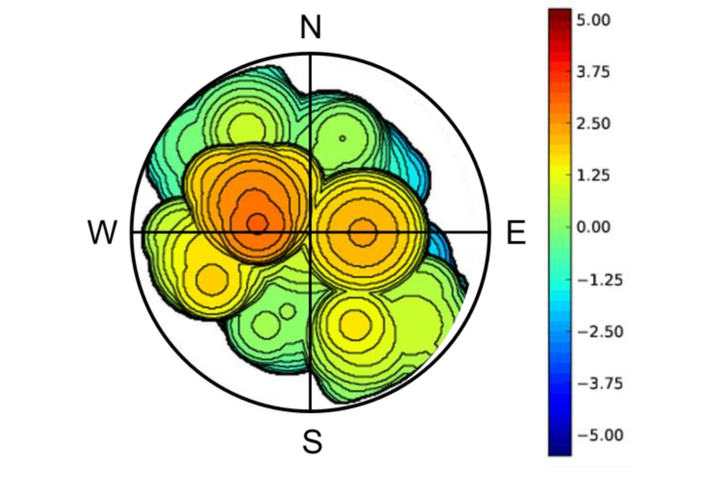

Settings:

Coordinating Atoms: N1, Dummy, Cl1
Z-axis defined by Zr atom z-negative
XZ-Plane defined by N1. Dummy, Cl1
Atomic radii scaled by 1.17
Sphere radius 5.5
Distance of coord. Point from centre 0.0
Mesh spacings for int. 0.05
H's includes

**%V$_{free}$**		**%V$_{bur}$**		**%V$_{tot}$**	
50.1		49.9		100.0	
**Quadrant**	**V$_{free}$**	**V$_{bur}$**	**V$_{total}$**	**%V$_{free}$**	**%V$_{bur}$**
SW	96.8	77.4	174.2	55.6	44.4
NW	71.7	102.5	174.2	41.2	58.8
NE	106.7	67.4	174.2	61.3	38.7
SE	73.6	100.6	174.2	42.3	57.7

8. References

[1] K. A. Erickson, M. P. Cibuzar, N. T. Mucha, R. Waterman, *Dalton Trans.* **2018**, *47*, 2138-2142.

[2] C. Chen, H. Wang, Y. Sun, J. Cui, J. Xie, Y. Shi, S. Yu, X. Hong, Z. Lu, *iScience* **2020**, *23*, 100985.

[3] Fulmer, G. R.; Miller, A. J. M.; Sherden, N. H.; Gottlieb, H. E.; Nudelman, A.; Stoltz, B. M.; Bercaw, J. E.; Goldberg, K. I., NMR Chemical Shifts of Trace Impurities: Common Laboratory Solvents, Organics, and Gases in Deuterated Solvents Relevant to the Organometallic Chemist. *Organometallics* **2010,** *29*, 2176-2179.

[4] Chen, S.; Wang, J.; Wang, H., Synthesis, characterization and pyrolysis of a high zirconium content zirconocene–polycarbosilane precursor without Zr–O bond. *Materials & Design* **2016,** *90*, 84-90.

[5] W. Schöniger, *Microchim. Acta*, 1955, **43**, 123-129.

[6] F. Mohr, Lehrbuch der chemisch-analytischen Titrirmethode*: Abtheilungen 1 und 2*, Vieweg, Braunschweig, 1856.

[7] Sheldrick, G. M. Crystal structure refinement with *SHELXL. Acta Cryst.*, 2008, **A64**, 112-122.

[8] Sheldrick, G. M. *SHELXT* - Integrated space-group and crystal-structure determination. *Acta Cryst.*, 2015, **C71**, 3-8.

[9] Diamond - Crystal and Molecular Structure Visualization, Crystal Impact - Dr. H. Putz & Dr. K. Brandenburg GbR, Kreuzherrenstr. 102, 53227 Bonn, Germany, http://www.crystalimpact.com/diamond.

[10] L. Falivene, R. Credendino, A. Poater, A. Petta, L. Serra, R. Oliva, V. Scarano, L. Cavallo, *Organometallics* **2016**, *35*, 2286-2293.

[11] *Gaussian 16, Revision C.01*, Frisch, M. J.; Trucks, G. W.; Schlegel, H. B.; Scuseria, G. E.; Robb, M. A.; Cheeseman, J. R.; Scalmani, G.; Barone, V.; Petersson, G. A.; Nakatsuji, H.; Li, X.; Caricato, M.; Marenich, A. V.; Bloino, J.; Janesko, B. G.; Gomperts, R.; Mennucci, B.; Hratchian, H. P.; Ortiz, J. V.; Izmaylov, A. F.; Sonnenberg, J. L.; Williams-Young, D.; Ding, F.; Lipparini, F.; Egidi, F.; Goings, J.; Peng, B.; Petrone, A.; Henderson, T.; Ranasinghe, D.; Zakrzewski, V. G.; Gao, J.; Rega, N.; Zheng, G.; Liang, W.; Hada, M.; Ehara, M.; Toyota, K.; Fukuda, R.; Hasegawa, J.; Ishida, M.; Nakajima, T.; Honda, Y.; Kitao, O.; Nakai, H.; Vreven, T.; Throssell, K.; Montgomery, J. A., Jr.; Peralta, J. E.; Ogliaro, F.; Bearpark, M. J.; Heyd, J. J.; Brothers, E. N.; Kudin, K. N.; Staroverov, V. N.; Keith, T. A.; Kobayashi, R.; Normand, J.; Raghavachari, K.; Rendell, A. P.; Burant, J. C.; Iyengar, S. S.; Tomasi, J.; Cossi, M.; Millam, J. M.; Klene, M.; Adamo, C.; Cammi, R.; Ochterski, J. W.; Martin, R. L.; Morokuma, K.; Farkas, O.; Foresman, J. B.; Fox, D. J. Gaussian, Inc., Wallingford CT, **2016**

[12] a) Becke, A. D. *Phys. Rev. A* **1988**, *38*, 3098–3100; b) Perdew, J. P. *Phys. Rev. B* **1986**, *33*, 8822–8824.

[13] a) Vosko, S. H.; Wilk, L.; Nusair, M. *Can. J. Phys.* **1980**, *58*, 1200–1211; b) Lee, C.; Yang, W.; Parr, R. G. *Phys. Rev. B* **1988**, *37*, 785–789; c) Miehlich, B.; Savin, A.; Stoll, H.; Preuss, H. *Chem. Phys. Lett.* **1989**, *157*, 200–206; d) Becke, A. D. *J. Chem. Phys.* **1993**, *98*, 5648–5652.

[14] Weigend, F.; Ahlrichs, R. *Phys. Chem. Chem. Phys.* **2005**, *7*, 3297–3305.

[15] a) Grimme, S.; Antony, J.; Ehrlich, S.; Krieg, H., *J. Chem. Phys.* **2010**, *132*, 154104; b) Grimme, S.; Ehrlich, S.; Goerigk, L., *J. Comput. Chem.* **2011**, *32*, 1456–1465.
